# Behavior of the E–E’ Bonds (E, E’ = S and Se) in Glutathione Disulfide and Derivatives Elucidated by Quantum Chemical Calculations with the Quantum Theory of Atoms-In-Molecules Approach

**DOI:** 10.3390/molecules23020443

**Published:** 2018-02-17

**Authors:** Satoko Hayashi, Yutaka Tsubomoto, Waro Nakanishi

**Affiliations:** Faculty of Systems Engineering, Wakayama University, 930 Sakaedani, Wakayama 640-8510, Japan; s143036@center.wakayama-u.ac.jp

**Keywords:** ab initio calculations, quantum theory of atoms-in-molecules (QTAIM), glutathione dichalcogenides, Monte-Carlo method

## Abstract

The nature of the E–E’ bonds (E, E’ = S and Se) in glutathione disulfide (**1**) and derivatives **2**–**3**, respectively, was elucidated by applying quantum theory of atoms-in-molecules (QTAIM) dual functional analysis (QTAIM-DFA), to clarify the basic contribution of E–E’ in the biological redox process, such as the glutathione peroxidase process. Five most stable conformers **a**–**e** were obtained, after applying the Monte-Carlo method then structural optimizations. In QTAIM-DFA, total electron energy densities *H*_b_(***r***_c_) are plotted versus *H*_b_(***r***_c_) − *V*_b_(***r***_c_)/2 at bond critical points (BCPs), where *V*_b_(***r***_c_) are potential energy densities at BCPs. Data from the fully optimized structures correspond to the static nature. Those containing perturbed structures around the fully optimized one in the plot represent the dynamic nature of interactions. The behavior of E–E’ was examined carefully. Whereas E–E’ in **1a**–**3e** were all predicted to have the weak covalent nature of the shared shell interactions, two different types of S–S were detected in **1**, depending on the conformational properties. Contributions from the intramolecular non-covalent interactions to stabilize the conformers were evaluated. An inverse relationship was observed between the stability of a conformer and the strength of E–E’ in the conformer, of which reason was discussed.

## 1. Introduction

**Scheme 1:**
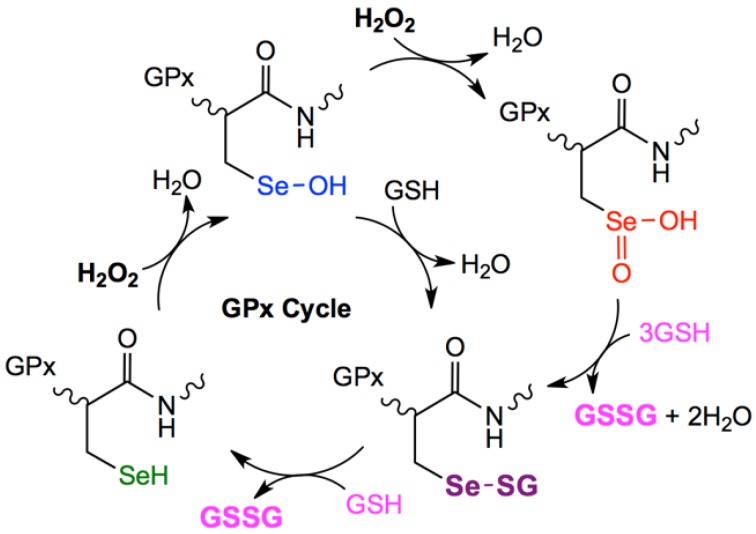
Catalytic mechanism, proposed for the antioxidant activity of GPx.

E–E’ bonds (E, E’ = S and Se) play a crucial role in biological redox processes [1]. High energy levels of HOMO and low energy levels of LUMO of the E–E’ bond must be the driving force for the high reactivity in the redox processes. The HOMO and LUMO of E–E’ would correspond to n_p_(E/E’) and σ* (E–E’), respectively, where n_p_(E/E’) denote the p-type lone pair orbitals of E and/or E’, while σ* (E–E’) corresponds to the σ*-orbital of E–E’. Glutathione disulfide (GSSG: **1**) has been widely used as a redox reagent in vitro. To facilitate the protein folding process, a mixture of **1** and glutathione (GSH) is often confirmed as the optimum condition if concentrations similar to those observed in vivo [2] are employed [3,4,5,6]. The reduced form of ribonuclease A will undergo disulfide-coupled folding and gain in structural stability in the presence of **1**, for example [7]. The detoxification of hydroperoxides in the glutathione peroxidase (GPx) process must be one of most important biological redox processes [8,9,10,11,12,13]. Scheme 1 summarizes a catalytic mechanism proposed for the antioxidant activity of GPx, which is a typical example of the intervention of E–E’ (E, E’ = S, Se) in biological reactions. According to this mechanism, two equivalents of GSH are oxidized to the corresponding oxidized disulfide in the overall process, while the hydroperoxide is reduced to water [14,15].

**Scheme 2:**
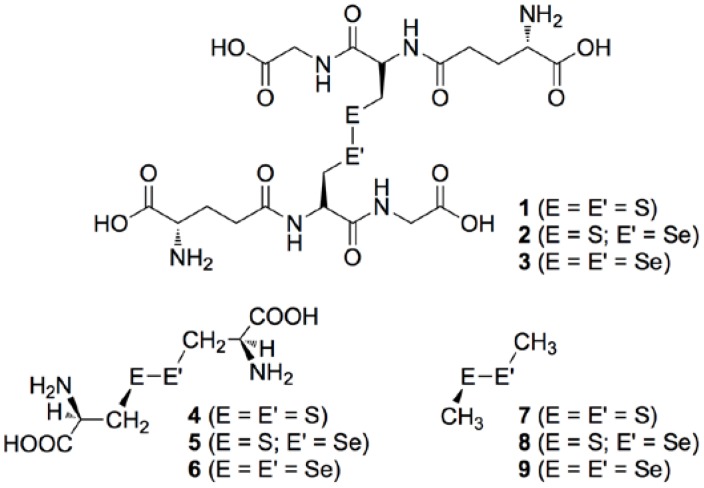
Structures of glutathione disulfide (**1**) and its derivatives (**2** and **3**) and *R*-cystine (**4**) and its derivatives (**5** and **6**), together with MeEE’Me (**7**–**9**).

The behavior of the S–S, S–Se and Se–Se bonds should be clarified, bearing in mind the role of these bonds in the antioxidant mechanism. It is highly important to elucidate the behavior of the S–S bond in **1** together with S–Se and Se–Se in two derivatives of **1** (compounds **2** and **3**, respectively). Scheme 2 illustrates the structures of **1**–**3**. There are many possibilities for the formation of intramolecular hydrogen bonds (HBs) in **1**–**3**, although the intermolecular HBs of the solute-solute and solute-solvent interactions must also be important in the real system. HBs in **1**–**3** must be considered in assessing the basic properties of **1**–**3** based on the calculated results, rather than performing calculations for a single molecule in vacuum. Scheme 2 also shows the structures of *R*-cystine and its derivatives **4**–**6** and MeEE’Me (compounds **7**–**9**).

**Figure 1:**
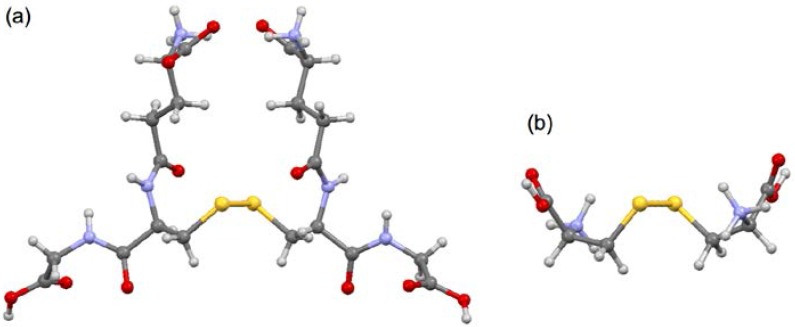
Structures of **1** (E = E’ = S) (**a**) and di-protonated form of **4** (E = E’ = S) (**b**), determined by X-ray analysis.

The structures of **1** and **4**, determined by X-ray crystallographic analysis, have been reported, although **4** is in the di-protonated form. Figure 1 shows these structures. The structure of **1** was observed as a half-extended form close to *C*_2_ symmetry with the formation of zwitterions [16]. The structure of **4** was reported as an extended form [17]. The extended form in the observed structure of **4** may be the result of the electrostatic repulsion of the positive charges developed on **4**^2+^. Many conformers must exist in such compounds, primarily due to the intramolecular HBs.

Reactions of **1** and/or **4** in vivo proceed under conditions with very large and highly complex species. However, the essence of the elementary processes is expected to be close to that of the typical chemical reactions. Therefore, it would be instructive to start with less complex species to clarify the behavior of the E–E’ bonds (E, E’ = S and Se). We reported the dynamic and static behavior of S–S in *R*-cystine (**4**) and S–Se and Se–Se in the derivatives of **4** (**5** and **6**, respectively), together with MeEE’Me **7**–**9** as references [18]. It is challenging to clarify the nature of the E–E’ bonds (E, E’ = S and Se) in glutathione disulfide and its derivatives (**1**–**3**), although the structures of **1**–**3** are considerably more complex relative to **4**–**6**, respectively. Structures **1**–**3** will have much more plausible HBs than **4**–**6**.

The QTAIM approach, introduced by Bader [19,20,21,22,23,24,25,26,27,28], enables us to analyze the nature of chemical bonds and interactions [11,12,13,14,15,16]. A bond critical point (BCP, ∗) is an important concept in QTAIM. BCP is a point where *ρ*(***r***) (charge density) reaches a minimum along the interatomic (bond) path, while it is a maximum on the interatomic surface separating the atomic basins. *ρ*(***r***) at BCP is denoted by *ρ*_b_(***r***_c_) and other QTAIM functions are denoted in a similar way. Interactions seem to be defined by the corresponding bond paths (BPs), but we must be careful to use the correct terminology with the concept. Interactions would be easily imaged by means of QTAIM if they can be defined as the corresponding BPs, especially for experimental chemists. However, it is demonstrated that the detection of the BPs between two atoms in a molecule emerging from natural alignment of the gradient vector held of the one-electron density of a molecule is neither necessary nor a sufficient condition for the presence of a chemical bond between those atoms [29,30,31,32,33,34]. In this connection, it is pointed out that the terms line paths (LPs) and line critical points (LCPs) should be used in place of BPs and BCPs, respectively [30]. Consequently, the dynamic and static nature in this work should be regarded as the investigation performed at LCPs on LPs corresponding to the E–E’ interactions. Nevertheless, the interactions expected for E–E’ are clearly detected by BPs with BCPs, which is another reason to use BPs and BCPs in this work. The structures of species can be described by molecular graphs, which are the sets of attractors (atoms) and BPs, together with BCPs, ring critical points (RCPs) and cage critical points (CCPs). We recently proposed QTAIM–DFA [35,36,37,38,39], as a tool for experimental chemists to analyze their own results concerning chemical bonds and interactions using their own images. QTAIM-DFA provides an excellent possibility to evaluate, classify, characterize and understand weak to strong interactions in a unified manner [40,41,42,43,44,45]. *H*_b_(***r***_c_) are plotted versus *H*_b_(***r***_c_) − *V*_b_(***r***_c_)/2 in QTAIM-DFA, where *H*_b_(***r***_c_) and *V*_b_(***r***_c_) are the total electron energy densities and potential energy densities, respectively, at BCPs. The QTAIM-DFA treatment can incorporate the classification of interactions based on the signs of *H*_b_(***r***_c_) and ∇^2^*ρ*_b_(***r***_c_) (Laplacian *ρ*), as shown in Scheme S1 of the Supplementary Materials, since the signs of *H*_b_(***r***_c_) − *V*_b_(***r***_c_)/2 must be equal to those of ∇^2^*ρ*_b_(***r***_c_), where (*ћ*^2^/8*m*) ∇^2^*ρ*_b_(***r***_c_) = *H*_b_(***r***_c_) − *V*_b_(***r***_c_)/2 (= *G*_b_(***r***_c_) + *V*_b_(***r***_c_)/2 while *H*_b_(***r***_c_) = *G*_b_(***r***_c_) + *V*_b_(***r***_c_), *G*_b_(***r***_c_): kinetic energy densities at BCPs) (see Equations (S1), (S2) and (S2’) of the Supplementary Materials). In our treatment, data for the perturbed structures around fully optimized structures are employed for the plots in addition to data for the fully optimized structures [35,36,37,38,39]. Data from the fully optimized structures are analyzed by the polar coordinate (*R*, *θ*) representation, which corresponds to the static nature of interactions. Data from the perturbed structures and a fully optimized structure are used to construct a curve. Each curve is analyzed in terms of the (*θ*_p_, *κ*_p_) parameters: *θ*_p_ corresponds to the tangent line of the plot and *κ*_p_ is the curvature. We proposed the concept of the “dynamic nature of interactions” based on (*θ*_p_, *κ*_p_) [35,36,37,38,39]. QTAIM-DFA is applied to typical chemical bonds and interactions. Rough criteria have been established that distinguish the chemical bonds and interactions in question from others. QTAIM-DFA and the criteria are explained in the Supplementary Materials, employing Schemes S1 and S2, Figure S1 and Equations (S1)–(S7). The basic concept of the QTAIM approach is also described in the Supplementary Materials.

The behavior of E–E’ (E, E’ = S and Se) in **1**–**3** is expected to be related to that in the glutathione peroxidase (GPx) process. The dynamic and static nature of E–E’ in **1**–**3** is elucidated by applying QTAIM-DFA. The same method is applied to E–E’ in **4**–**6** and **7**–**9** to reexamine the nature of these bonds. We present the results of the theoretical elucidation of the nature of the E–E’ bonds in **1**–**6** with QTAIM-DFA to better understand the role of E–E’ in the antioxidant activity of GPx. Quantum chemical (QC) calculations are also applied to examine the structural features of **1**–**6**. The E–E’ bonds in **1**–**6** are classified and characterized by employing the criteria and the behavior of the bonds in **7**–**9** as references.

## 2. Methodological Details in Calculations

The structures were optimized employing the Gaussian 09 programs [46] unless otherwise noted. For each species **1**–**6**, five conformers were optimized with the 6-311+G(3d) basis sets for S and Se, and with the 6-311++G(d, p) basis sets for O, N, C and H [47,48,49,50]. The basis set system is called BSS-A in this paper. The DFT level of M06-2X [51] is applied to the calculations. Before the final optimizations, the full conformer search with the Monte-Carlo method in Spartan 02 [52] was applied to each of **1**–**6**. At least six thousand and five hundred conformers were generated for each of **1**–**3** with the Merck Molecular Force Field (MMFF) method [53]. The most stable thirty independent conformers from the Monte-Carlo method were optimized using the 3–21G basis sets at the B3LYP level [54,55] for each of **1**–**3**. Next, the most stable fifteen conformers were optimized with M06-2X/6-31G(d) for each conformer, as predicted with B3LYP/3-21G of the Gaussian09 program. The most stable five conformers from M06-2X/6-31G(d) were further optimized with BSS-A at the M06-2X level (M06-2X/BSS-A). The final five optimized conformers were confirmed by the frequency analysis for each of **1**–**3**. These five conformers are called **a**, **b**, **c**, **d** and **e**, where conformer **a** is the most stable among the five, followed by **b**, then **c**, then **d** and then **e**. In the case of **4**–**6**, seven hundred and twenty conformers were generated for each species with the PM3 method [56]. Similar to the case for **1**–**3**, the five most stable conformers (**a**–**e**) were determined for each of **4**–**6**. The structures of **7** and **9** are optimized retaining the *C*_2_ symmetry, while that of **8** is retaining the *C*_1_ symmetry. The population analysis has also been performed by the natural bond orbital method [57] at M06-2X/BSS-A level of theory using natural bond orbital (NBO) program [58].

QTAIM functions were calculated using the Gaussian 09 program package at the same level of DFT theory (M06-2X/BSS-A), and the data were analyzed with the AIM2000 program [19,59]. Normal coordinates of internal vibrations (NIV) obtained by the frequency analysis were employed to generate the perturbed structures [38,39]. This method is called NIV and explained in Equation (1). The *k*-th perturbed structure in question (**S***_kw_*) was generated by the addition of the normal coordinates of the *k*-th internal vibration (**N***_k_*) to the standard orientation of a fully optimized structure (**S**_o_) in the matrix representation. The coefficient *f_kw_* in Equation (1) controls the difference in structures between **S***_kw_* and **S**_o_: *f_kw_* is determined to satisfy Equation (2) for an interaction in question, where *r* and *r*_o_ show the interaction distances in question in the perturbed and fully optimized structures, respectively, with *a*_o_ representing the Bohr radius (0.52918 Å) [12,13]. The perturbed structures with NIV correspond to those where *r* has been elongated or shortened by 0.05*a*_o_ or 0.1*a*_o_, relative to *r*_o_, in the fully optimized structures [60]. The selected motion must be most effectively localized on the interaction in question among the zero-point internal vibrations. **N***_k_* of five digits are used to predict **S***_kw_*:

(1) $$S_{kw}=S_{o}+f_{kw}•N_{k}$$

(2) $$r=r_{o}+wa_{o}\;\left(w=\left(0\right),\pm 0.05\;\mathrm{and}\;\pm 0.1;\;a_{o}=0.52918\;Å\right)$$

(3) $$y=c_{o}+c_{1}x+c_{2}x^{2}+c_{3}x^{3}\;\left(R_{c^{2}}:\;\mathrm{square}\;\mathrm{of}\;\mathrm{correlation}\;\mathrm{coefficient}\right)$$

*H*_b_(***r***_c_) are plotted versus *H*_b_(***r***_c_) − *V*_b_(***r***_c_)/2 for data from the five points of *w* = 0, ±0.05 and ±0.1 in Equation (2) in QTAIM-DFA. Each plot is analyzed using a regression curve of the cubic function as shown in Equation (3), where (*x*, *y*) = (*H*_b_(***r***_c_) − *V*_b_(***r***_c_)/2, *H*_b_(***r***_c_)) (*R*_c_^2^ (square of correlation coefficient) > 0.99999, usually) [61].

## 3. Results and Discussion

### 3.1. Optimized Structures for Conformers of ***1**–**6*** with M06-2X/BSS-A, Together with ***7**–**9***

The five conformers (**a**–**e**) for each of **1**–**6** are optimized with M06-2X/BSS-A, which are called **1a**–**1e**, **2a**–**2e**, **3a**–**3e**, **4a**–**4e**, **5a**–**5e** and **6a**–**6e**, respectively. The whole set of species is also described by **1a**–**6e**, if necessary. Each conformer is optimized as a non-extended form. The total energies evaluated for ***nx*** (***n*** = **1**–**6**; ***x*** = **a**–**e**) (*E*(***nx***)) are defined to satisfy Equation (4). The relative energies for the conformers of **1a**–**1e** [*E*_rel_(**1*x***: ***x*** = **a**–**e**)] are evaluated from **1a** with M06-2X/BSS-A, so are *E*_rel_(***nx***: ***n*** = **2**–**6**; ***x*** = **a**–**e**). The structures of zwitterions are confirmed between the amino and carboxyl groups at the terminal positions of the main chains in **1a**–**3e**, except for **1b** and **1e**. Only one conformer was optimized for each of **7**–**9** with M06-2X/BSS-A, as expected. Table 1 collects the structural parameters of the *r*(E, E’) distances and the torsional angles of *φ*(CEE’C) (= *φ*_A_) for **1a**–**6a** and **7**–**9**, optimized with M06-2X/BSS-A, together with the relative energies *E*_rel_ (= *E*(***nx***) – *E*(***n*a**) (***n*** = **1**–**6**; ***x*** = **a**–**e**)):

(4) E(na)≤E(nb)≤E(nc)≤E(nd)≤E(ne) (n=1–6)

The *r*(S, S) values for **1a**–**1e** are predicted to be larger than those of **7**. The differences in *r*(S, S) for **1a**–**1e** (Δ*r*(S, S: **1*x***) = *r*(S, S: **1*x***) − *r*(S, S: **7**), where ***x*** = **a**–**e**) are 0.02 Å < Δ*r*(S, S: **1*x***) < 0.03 Å for **1a**–**1c**, Δ*r*(S, S: **1*x***) < 0.01 Å for **1d** and Δ*r*(S, S: **1*x***) ≈ 0.20 Å for **1e**. Similarly, the Δ*r*(E, E’) values are less than or very close to 0.01 Å for ***n*a**–***n*e** (***n*** = **2**–**6**), except for Δ*r*(E, E’) ≈ 0.015 Å for **2e**, **4a**, **5d** and **5e** with Δ*r*(E, E’) ≈ 0.03 Å for **3d**. There must be a specific reason for the unexpectedly large value of 0.20 Å for Δ*r*(E, E’: **1e**). Three S, S and O atoms align linearly in **1e**, which is explained by assuming the formation of hypervalent interactions of S_2_O σ(3c–4e) of the n_p_(O)→σ*(S–S) type (see Figure 4). In this interaction, σ*(S–S) accepts electrons from n_p_(O). As a result, the S–S bond must be unexpectedly elongated relative to the usual length, and the O---S distance will be substantially shortened relative to the sum of the vdW radii. The O---S distance is predicted to be 2.7714 Å, which is shorter than the sum of the vdW radii by 0.55 Å. The S_2_O σ(3c–4e) model explains the predicted result for **1e**, reasonably well.

**Figure 2:**
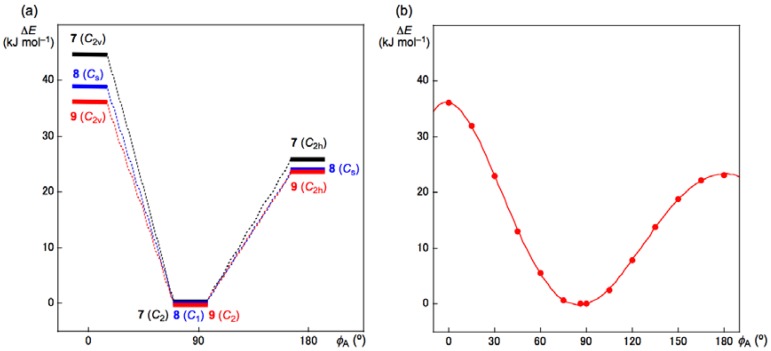
Plots of deformation energy (Δ*E*) versus *φ*_A_. For **7**–**9** (**a**) and for **9** (**b**).

In the case of *φ*(CEE’C) (= *φ*_A_), the values for **1a**–**6e** from the corresponding values of **7**–**9** are given by Δ*φ*_A_(E, E’: ***nx***) = *φ*_A_(E, E’: ***nx***) − *φ*_A_(E, E’: MeEE’Me), where ***n*** = **1**–**6**; ***x*** = **a**–**e**; and E, E’ = S and Se. The absolute values of *φ*_A_ will be used to estimate Δ*φ*_A_. The magnitudes of the values are Δ*φ*_A_(E, E’: ***nx***) ≈ 58° for **3d**, 31° < Δ*φ*_A_(E, E’: ***nx***) < 35° for **1a**–**1c** and **1e**, Δ*φ*_A_(E, E’: ***nx***) ≈ 25° for **2b**, and 10° ≤ Δ*φ*_A_(E, E’: ***nx***) ≤ 20° for **1d**, **3e**, **4e** and **6d**. The magnitudes of Δ*φ*_A_(E, E’: ***nx***) are less than 10° (−10° ≤ Δ*φ*_A_(E, E’: ***nx***) ≤ 10°) for others, except for Δ*φ*_A_(E, E’: ***nx***) ≈ –12° for **5d** and **6d** and Δ*φ*_A_(E, E’: ***nx***) ≈ −20° for **2e**, **4a** and **5e**. The results must be the reflection from the easy deformation in *φ*_A_. To evaluate the energy for the deformation in *φ*_A_, **7**–**9** were optimized assuming *φ*_A_ = 0° and 180°, in addition to the fully optimized structures (85° ≤ *φ*_A_ ≤ 86°). They were optimized to be **7** (*C*_2v_), **8** (*C*_s_) and **9** (*C*_2v_) at *φ*_A_ = 0° and **7** (*C*_2h_), **8** (*C*_s_) and **9** (*C*_2h_) at *φ*_A_ = 180°. In the case of **9**, the structures were further optimized with *φ*_A_ fixed every 15° for 0° ≤ *φ*_A_ ≤ 180°. The results are summarized in Table S1 of the Supplementary Materials. Figure 2 shows the plot of the energies for the optimized structures versus *φ*_A_. The energy seems less than 15 kJ mol^−1^ for 45° ≤ *φ*_A_ ≤ 135° in **9**. The energy for the deformation of *φ*_A_ in **7** and **8** seems comparable to that in **9**. The very easy deformation in *φ*_A_ is well demonstrated, exemplified by **7**–**9**, which supports the results shown in Table 1. Such easy deformation in *φ*_A_ is also reported for some dichalcogenides [62].

### 3.2. Structural Feature of ***1a**–**6a*** and ***7**–**9***

**Figure 3:**
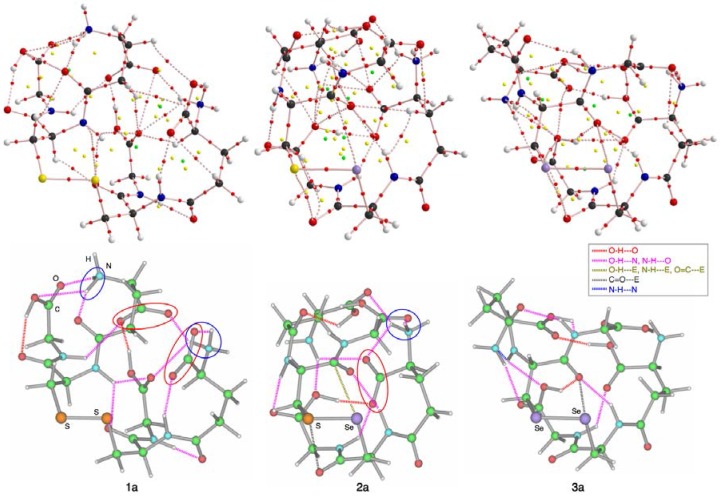
Molecular graphs of **1a**, **2a** and **3a**, drawn on the optimized structures (top) and the intramolecular non-covalent interactions, corresponding to BPs in the molecular graphs, drawn on the optimized structures. The red and blue circles show the zwitter ionic –NH_3_^+^ and –COO^−^ moieties, respectively (bottom). The energies of **1a**, **2a** and **3a** are employed as the standards for **1a**–**1e**, **2a**–**2e** and **3a**–**3e**, respectively.

**Figure 4:**
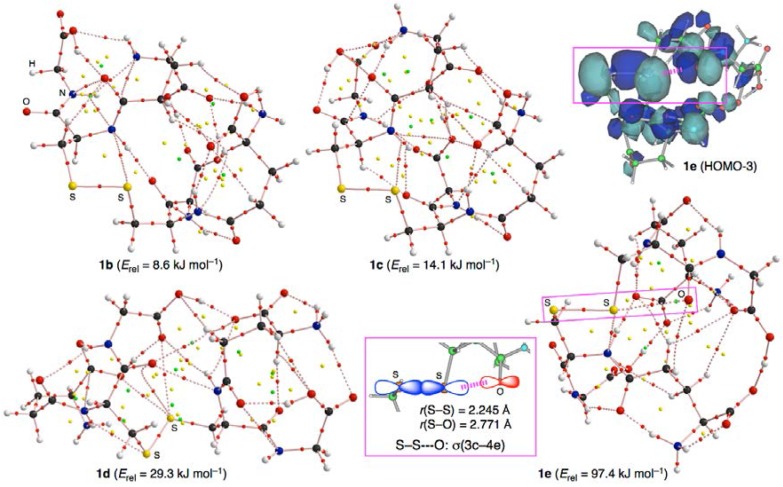
Molecular graphs of **1b**–**1e**, drawn on the optimized structures and the molecular orbital of HOMO-3 for **1e** with the orbital interaction map explaining the HOMO-3.

**Figure 5:**
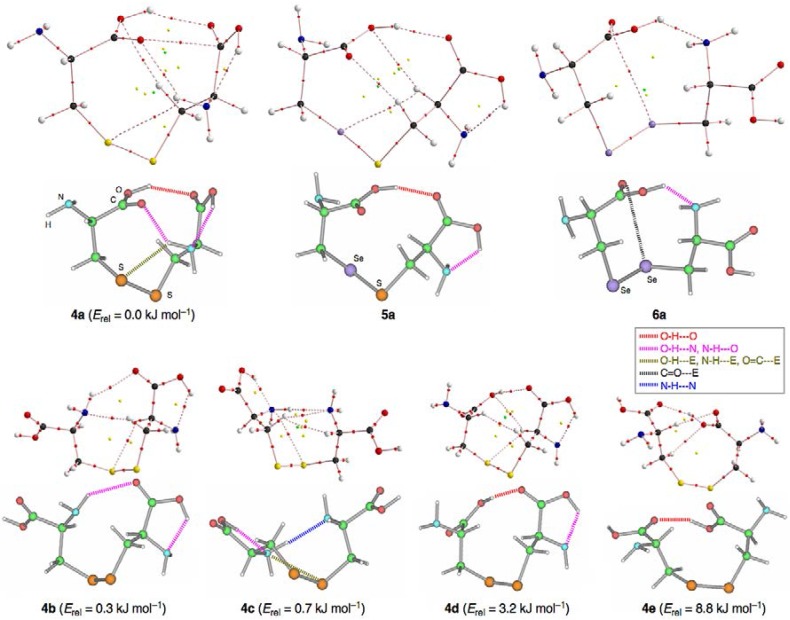
Molecular graphs of **4a**–**6a**, drawn on the optimized structures and the intramolecular non-covalent interactions, corresponding to BPs in the molecular graphs, drawn on the optimized structures (top two). Molecular graphs of **4b**–**4e**, drawn on the optimized structures and the intramolecular non-covalent interactions, corresponding to BPs in the molecular graphs, drawn on the optimized structures (bottom two).

Figure 3 illustrates the molecular graphs of **1a**–**3a**, drawn on the optimized structures, together with the optimized structures containing the non-covalent interactions. Figure 4 shows the molecular graphs of **1b**–**1e**, drawn on the optimized structures. Molecular graphs of **2b**–**2e** and **3b**–**3e** are drawn in Figures S2 and S3 of the Supplementary Materials, respectively. Figure 5 illustrates the molecular graphs of **4a**, **5a**, **6a** and **4b**–**4e**, drawn on the optimized structures and the optimized structure. Molecular graphs of **5b**–**5e**, **6b**–**6e** and **7**–**9** are drawn in Figures S4–S6 of the Supplementary Materials, respectively.

The structural features of **7**–**9** are described, first. Only classical chemical bonds are detected in the molecular graphs of **7**–**9**, as shown in Figure S6 of the Supplementary Materials. Namely, no interactions other than the classical chemical bonds contribute to the interactions in **7**–**9**. The structural features of **4**–**6** are examined next. Various types of intramolecular non-covalent interactions are detected in **4a**–**6e**, which are the HB type of O–H---O, O–H---N, N–H---O, N–H---N, O–H---E(E’) and N–H---E(E’), where E, E’ = S and Se. The E---π type of C = O---E(E’) and O = C---E(E’) are also detected. The conformers must be stabilized through the energy lowering effect by the formation of the intramolecular attractive interactions. The HB and E---π type non-covalent interactions contribute to stabilize the conformers. The HB and E---π type interactions in the molecular graphs are drawn on the optimized structures with different colors for the different interaction types to aid in visualization. The interactions are drawn for O–H---O in red, O–H---N and N–H---O in pink, N–H---N in blue, O–H---E(E’), N–H---E(E’) and O = C---E(E’) in olive and C = O---E(E’) in grey (see, Figure 5). The numbers of the intramolecular non-covalent interactions are counted separately by the interaction types, which are differentiated by the colors. The results are collected in Table S2 of the Supplementary Materials. Indeed, the stability of the conformers is expected to relate to the numbers, but they must be stabilized by the total energy of the intramolecular non-covalent interactions. The C–H---X interactions are also detected, however, they are neglected, since they would not make a significant contribution to stabilizing the conformers.

The molecular graphs for **1a**–**3e** are very complex, with many intramolecular non-covalent interactions. An effort is made to classify the interactions in **1a**–**3e**, as in **4a**–**6e**. The non-covalent interactions appearing in the molecular graphs of **1a**–**3e** are similarly drawn on the optimized structures, as shown in Figure 3. The numbers of the intramolecular non-covalent interactions in **1a**–**3e** are also counted separately based on the type of interaction. The results are collected in Table S2 of the Supplementary Materials. The numbers seem to be correlated to the stability of the conformers. However, it must be difficult to estimate numerically the stability of the conformers based on the numbers. The stability must be controlled by the total energy of the intramolecular interactions.

Nevertheless, it is very important to understand how *E*_rel_ for the conformers are determined by the intramolecular non-covalent interactions in the conformers of **1a**–**3e**, as a whole. How can *E*_rel_ be evaluated based on the contributions from the non-covalent interactions? We searched for a method to evaluate the stability of the conformers based on the overall intramolecular non-covalent interactions. Then, we devised a method to evaluate the contributions, which is discussed next.

### 3.3. Factors Determining the Relative Energies of ***1a**–**6a***

**Scheme 3:**
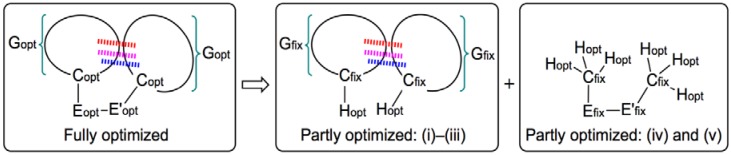
Proposed method to evaluate the contributions from the G---G intramolecular non-covalent interactions in GEE’G. The processes illustrated in the scheme are explained in the text.

The proposed method is explained in Scheme 3 with Equations (5)–(7). The evaluation process is as follows: (i) GEE’G is fully optimized; (ii) E and E’ in the optimized GEE’G are replaced by H and H; (iii) The structural parameters for the two replaced H atoms are (partially) optimized, with other atoms fixed at the fully optimized geometry; (iv) The structural parameters of CEE’C are fixed at the fully optimized positions, and H atoms are added on each side of CEE’C in place of the organic ligands to give H_3_CEE’CH_3_; (v) The structural parameters of the six H atoms are optimized:

(5) $$\begin{array}{ll} E{\left(\mathrm{GEE}\prime G\right)}_{\mathrm{opt}}= & 2E\left(G_{\mathrm{fix}}C_{\mathrm{fix}}-H_{\mathrm{opt}}\right)\\ & +E\left[{\left(H_{\mathrm{opt}}\right)}_{3}C_{\mathrm{fix}}E_{\mathrm{fix}}-{E\prime }_{\mathrm{fix}}C_{\mathrm{fix}}{\left(H_{\mathrm{opt}}\right)}_{3}\right]-2E{\left({\mathrm{CH}}_{4}\right)}_{\mathrm{opt}}\\ & +\alpha \;\left(\alpha :\;\mathrm{almost}\;\mathrm{constant}\right) \end{array}$$

(6) $$E_{\mathrm{rel}}{\left(\mathrm{GEE}\prime G\right)}_{\mathrm{opt}}=2E_{\mathrm{rel}}\left(G_{\mathrm{fix}}-H_{\mathrm{opt}}\right)+E_{\mathrm{rel}}[{\left(H_{\mathrm{opt}}\right)}_{3}C_{\mathrm{fix}}E_{\mathrm{fix}}-{E\prime }_{\mathrm{fix}}C_{\mathrm{fix}}{\left(H_{\mathrm{opt}}\right)}_{3}]+\alpha_{\mathrm{rel}}$$

(7) $$E_{\mathrm{rel}}{\left(\mathrm{GEE}\prime G\right)}_{\mathrm{opt}}\approx 2E_{\mathrm{rel}}\left(G_{\mathrm{fix}}-H_{\mathrm{opt}}\right)+E_{\mathrm{rel}}\left[{\left(H_{\mathrm{opt}}\right)}_{3}C_{\mathrm{fix}}E_{\mathrm{fix}}-{E\prime }_{\mathrm{fix}}C_{\mathrm{fix}}{\left(H_{\mathrm{opt}}\right)}_{3}\right]$$

The method shown in Scheme 3 will evaluate the intramolecular G---G interactions and the deformation energies around CEE’C but not the steric factor around the E–E’ moiety. Equation (5) shows the relationship between *E*(GEE’G) for the fully optimized structure and the energies evaluated by the proposed method, where α shows the errors in energy between *E*(GEE’G) and the components, which contains the steric factor around the E–E’ moiety. The relationship for *E*_rel_(GEE’G) is shown in Equation (6), where *E*(CH_4_) disappears. As shown in Equation (7), *E*_rel_(GEE’G)_opt_ can be approximated as 2*E*_rel_(G_fix_–H_opt_) + *E*_rel_[(H_opt_)_3_C_fix_E_fix_–E’_fix_C_fix_(H_opt_)_3_] if α is almost constant. The *E*_rel_ values are given as the values from the most stable conformers in **1a**–**6a**, if applied to **1a**–**6e**, respectively. The results of the calculations for **1a**–**6e** are collected in Table S3 of the Supplementary Materials, where 2*E*_rel_(G_fix_–H_opt_) and *E*_rel_[(H_opt_)_3_C_fix_E_fix_–E’_fix_C_fix_(H_opt_)_3_] are abbreviated as *E*_rel_(2GH)_p-opt_ and *E*_rel_(MeSSMe)_p-opt_, respectively (p-opt: partially optimizations).

**Figure 6:**
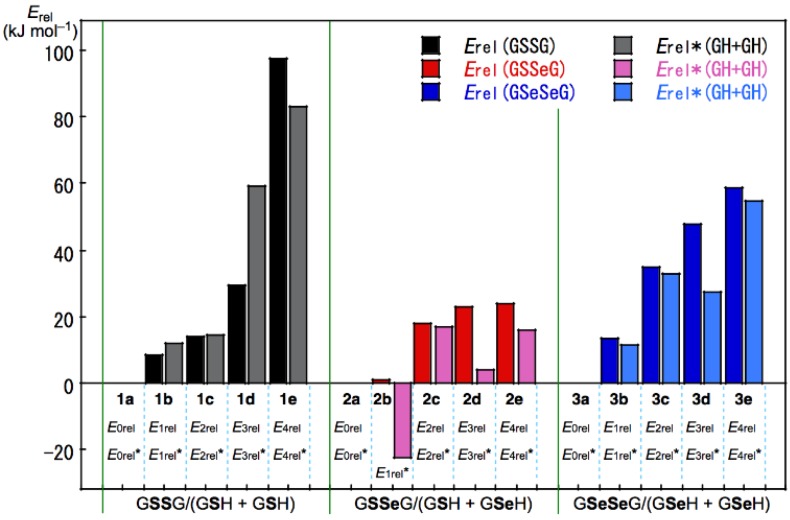
Plots of E_rel_ of GEE’G and (2G − H + MeEE’Me) for **1a**–**e** (GSSG), **2a**–**e** (GSSeG) and **3a**–**e** (GSeSeG), evaluated with M06-2X/BSS-A.

Figure 6 shows the plot of *E*_rel_(2GH + MeEE’Me)_p-opt_ for **1a**–**3e**, together with *E*_rel_(GEE’G)_opt_. The *E*_rel_(2GH + MeEE’Me)_p-opt_ values seem to match the *E*_rel_(GEE’G)_opt_ values for **1a**–**3e**, except for **2b** and **2d**. Indeed, the relative stabilities of the conformers for the S–S and Se–Se species are explained well by the treatment, but they are not explained well by treatment for the S–Se species, especially for **2b** and moderately for **2d**. Other factors, such as the steric factor around the S–Se moiety, could be important in this case. The very large magnitude of *φ*_A_ in **2b** (110.1°) relative to other species (65.0–85.6°) is responsible for this result. The deviation in **2b**, due to *E*_rel_(2GH + MeSSeMe)_p-opt_ (−18.4 kJ mol^−1^) versus *E*_rel_(GSSeG)_opt_ (1.0 kJ mol^−1^), is due to the high stability of *E*_rel_(2GH)_p-opt_ (–22.7 kJ mol^−1^), which is due to the reflection of the C–H optimizations in 2GH from the unstable position of C–H by *φ*_A_ for **2b** (110.1°). The smaller magnitudes in *E*_rel_ for **2a**–**2e** may lead to greater deviations (see Table S3 of the Supplementary Materials). A similar plot for **4a**–**6e** is shown in Figure S7 of the Supplementary Materials. The relationship between *E*_rel_(2CysH + MeEE’Me)_p-opt_ and *E*_rel_(CysEE’Cys)_opt_ seems unclear for **4a**–**6e**. After clarification of the structural features of **1a**–**6e**, the contour plots and negative Laplacians are examined next.

### 3.4. Contour Plots and Negative Laplacian around the E-∗-E’ Bonds in ***1a**–**6e***

**Figure 7:**
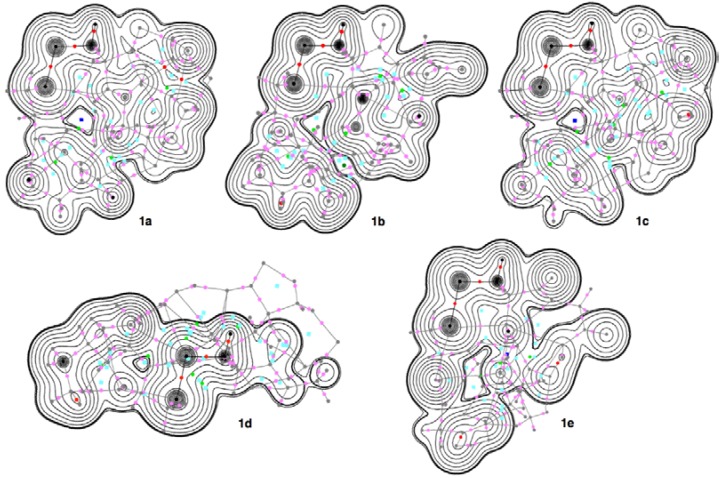
Contour plots of *ρ*_b_(***r***_c_) drawn on the SSC planes of **1a**–**1e**, together with BCPs (red solid dots on the plane and pink solid dots out of the plane), RCPs (ring critical points: deep green solid squares on the plane and green solid squares out of the plane), CCPs (cage critical points: blue solid dots on the plane and cyan solid dots out of the plane) and bond paths (black solid lines). The contours (*ea*_o_^−3^) are at 2*^l^* (*l* = ±8, ±7, …., 0) with the heavy line of 0.0047 for the molecular surface.

**Figure 8:**
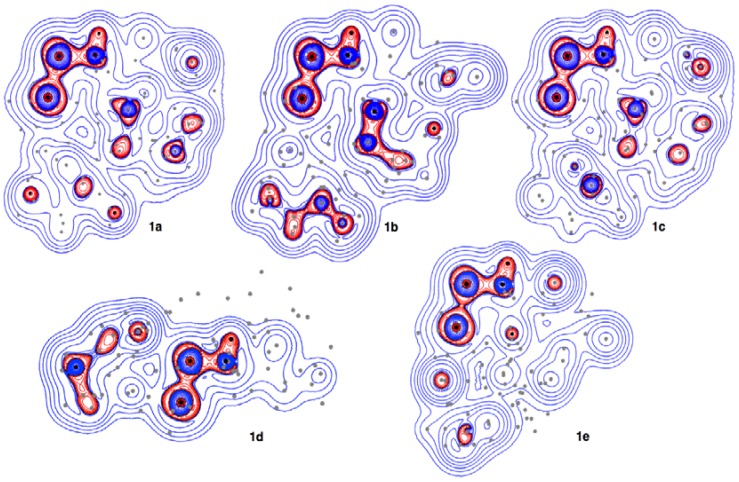
Negative Laplacians drawn on the SSC planes of **1a**–**1e**. The negative areas are shown in red and the positive areas are in blue.

Figure 7 shows the contour plots of *ρ*(***r***), exemplified by **1a**–**1e**, which are drawn on an SSC plane of **1a**–**e**. The plots show that each BCP on E-∗-E’ exists at the three-dimensional saddle point of *ρ*(***r***). Figure 8 illustrates the Negative Laplacian, exemplified by **1a**–**1e**, similarly drawn on an SSC plane. All BCP on E-∗-E’ of **1a**–**1e** exist in the red area of the plots, which means that the BCPs are all in the range of ∇^2^*ρ*_b_(***r***_c_) < 0. Therefore, E-∗-E’ of **1a**–**1e** are classified by SS (shared shell) interactions. (See also Figure S8 of the Supplementary Materials for the trajectory plots for **1a**–**1e**.)

### 3.5. Application of QTAIM-DFA to the E–E’ Bonds in ***1a**–**6e***

**Figure 9:**
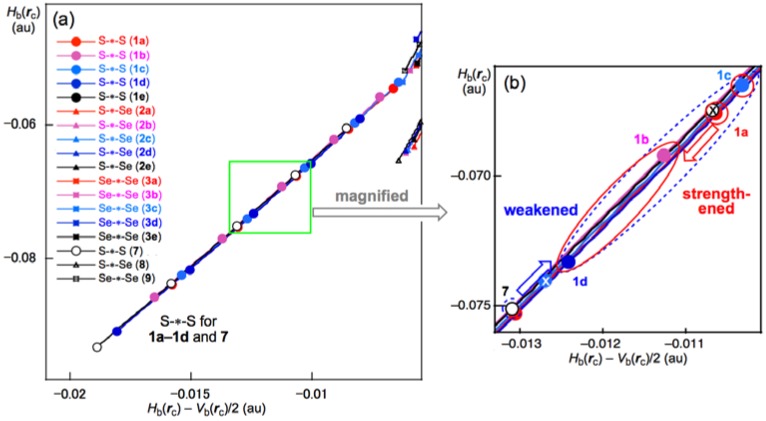
Plots of *H*_b_(***r***_c_) versus *H*_b_(***r***_c_) − *V*_b_(***r***_c_)/2 for **1a**–**1e** and **7**. (**a**) Whole plot and (**b**) magnified view of the data around the fully optimized structures.

**Figure 10:**
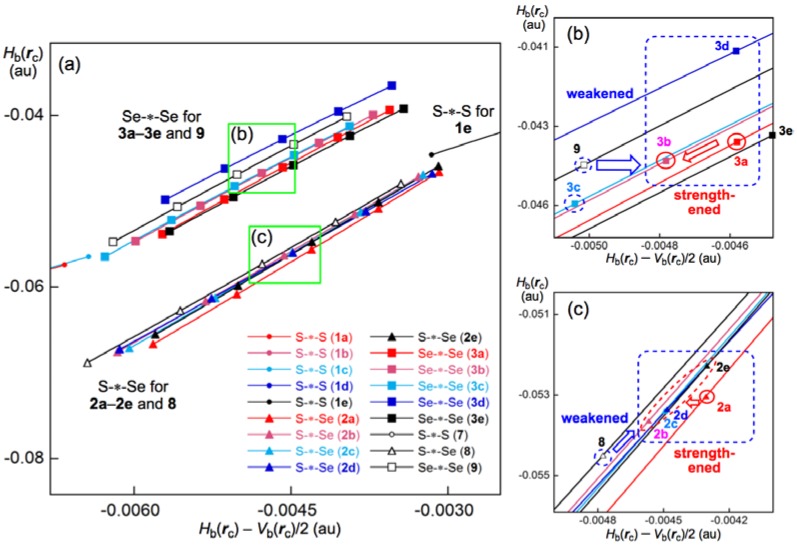
Plots of *H*_b_(***r***_c_) versus *H*_b_(***r***_c_) − *V*_b_(***r***_c_)/2 for **2a**–**3e**, **8** and **9**. (**a**) Whole plot; (**b**) magnified view of the data around the fully optimized structures of **3a**–**3e** and **9**; and (**c**) magnified view of the data around the fully optimized structures of **2a**–**2e** and **8**.

QTAIM functions are calculated for **1a**–**6e** and **7**–**9**. Table 2 collects the results for **1a**–**3e** and **7**–**9**. Figure 9 shows the plots of *H*_b_(***r***_c_) versus *H*_b_(***r***_c_) − *V*_b_(***r***_c_)/2 for **1a**–**1d** and **7**, where the data for **1e** do not appear in the plotted area. Figure 10 displays the plots of *H*_b_(***r***_c_) versus *H*_b_(***r***_c_) − *V*_b_(***r***_c_)/2 for **2a**–**3e**, **8** and **9**. Figure 9 and Figure 10 also contain magnified representations of the data around the fully optimized structures. 

All data in Table 2 and the perturbed structures of **1a**–**3e** and **7**–**9** are plotted in Figure S9 of the Supplementary Materials. All data for the fully optimized structures of **1a**–**6e** and **7**–**9** appear in the range of *H*_b_(***r***_c_) < 0 and *H*_b_(***r***_c_) − *V*_b_(***r***_c_)/2 < 0. Therefore, the E–E’ interactions of **1a**–**6e** and **7**–**9** are all classified by SS (shared shell) interactions, irrespective of the substantial elongation of the S-∗-S bond length by the perturbation that occurred in the conformers, such as S–S in **1e**. The plots are analyzed according to Equations (S3)–(S6) of the Supplementary Materials, by applying QTAIM-DFA.

The QTAIM-DFA parameters of (*R*, *θ*) and (*θ*_p_, *κ*_p_) are also collected in Table 2, together with the frequencies (*ν*) and force constants (*k*_f_) corresponding to the E-∗-E′ bonds in question. While the data for **4a**–**4e** are plotted in Figure S10, those for **5a**–**6e** are in Figure S11 of the Supplementary Materials, together with those for **7**–**9**. Similarly, the plots are analyzed to give the QTAIM-DFA parameters of (*R*, *θ*) and (*θ*_p_, *κ*_p_). The parameters are collected in Table S4 of the Supplementary Materials, together with the frequencies (*ν_n_*) and force constants (*k*_f_) corresponding to the E-∗-E′ bonds in question.

### 3.6. Nature of the E–E’ Bonds in ***1a**–**6e***

The E-∗-E′ bonds in **1a**–**6e** are classified and characterized based on *R*, *θ* and *θ*_p_ values, employing those of the standard interactions given in Scheme S2 of the Supplementary Materials as a reference. Before discussing the nature of the E-∗-E’ bonds in **1a**–**6e** and **7**–**9**, it would be instructive to survey the related criteria. Interactions will be classified as SS or CS interactions for *θ* > 180° or *θ* < 180°, respectively, which correspond to *H*_b_(***r***_c_) − *V*_b_(***r***_c_)/2 < 0 and *H*_b_(***r***_c_) − *V*_b_(***r***_c_)/2 > 0, respectively. The *θ*_p_ values play an important role in characterizing the interactions. For SS interactions with *θ* > 180°, *θ*_p_ > 190° is tentatively assigned, where *θ*_p_ = 190° corresponds to *θ* = 180° for typical interactions. The covalent interactions will be sub-divided depending on the values of *R*. The (classical) covalent interactions will be called strong (Cov_strong_) if *R* > 0.15 au; therefore, they should be called weak (Cov_weak_) when *R* < 0.15 au.

The *R* value of 0.076 au (<0.15 au) is predicted for MeS-∗-SMe (**7**), and those for S-∗-S, S-∗-Se and Se-∗-Se in **1a**–**6e**, **8** and **9**, examined in this work, are all less than 0.076 au. Therefore, the Cov_strong_ interactions are not detected in this work. As shown in Table 2, the (*θ*, *θ*_p_) values for S-∗-S in **1a**–**1e** are (188.8–189.6°, 197.2–197.6°) for **1a**–**1d** with (181.9°, 193.8°) for **1e**. The value for **1e** is apparently smaller than those for **1a**–**1d** due to the elongation of S-∗-S by the formation of S_2_O σ(3c–4e) in **1e**. This means that S-∗-S in **1e** should be (much) weaker than those in **1a**–**1d**. Nevertheless, the S-∗-S interaction in **1e** is classified by the SS interaction and characterized as Cov_weak_ in nature (SS/Cov_weak_). All S-∗-S interactions in **1a**–**1d** are, of course, predicted to have the nature of (SS/Cov_weak_). The (*θ*, *θ*_p_) values for S-∗-Se in **2a**–**2e** are (184.7–184.9°, 188.1–188.7°). Therefore, the S-∗-Se interactions are also classified by the SS interactions and characterized as Cov_weak_ in nature (SS/Cov_weak_), although the *θ*_p_ values are slightly less than 190°. In the case of Se-∗-Se in **3a**–**3e**, the (*θ*, *θ*_p_) values are (185.9–186.4°, 189.0–189.8°). The Se-∗-Se interactions are predicted to have the nature of (SS/Cov_weak_), similar to the cases of **1a**–**2e**. Note that S-∗-S in **1e** is predicted to be weaker than S-∗-Se in **2a**–**2e** and Se-∗-Se in **3a**–**3e** by *R* and *θ*, although the trend is reversed for *θ*_p_. Indeed, Se-∗-Se in **3a**–**3e** is predicted to be stronger than S-∗-Se in **2a**–**2e** by *θ* and *θ*_p_, and the inverse trend is true for *R*. The E-∗-E′ bonds in **4a**–**6e** are all predicted to have the nature of (SS/Cov_weak_), as are the bonds in **7**–**9**, similar to the case for **1a**–**3e**.

### 3.7. Factors that Stabilize the E–E’ Bonds and the Conformers

The S–S bonds of **1a**–**1e** are predicted to be less stable than that of **7**. The S-∗-S bond in **1a**–**1e** is predicted to be weaker in the order shown in Equation (8), where **1e** is significantly destabilized due to the elongation by the formation of S_2_O σ(3c–4e). The order for the strength of S-∗-S seems to exhibit almost the reverse trend of stability compared with the conformers. A similar order is predicted for S-∗-Se of **2a**–**2e** with **8**. Equation (9) shows the order for S-∗-Se, where **2a** seems substantially destabilized. On the other hand, the trend is not so clear for Se-∗-Se in **3a**–**3e**. The predicted order for Se-∗-Se is given in Equation (10). The strength of E-∗-E’ seems to show a trend with almost inverse stability compared with the conformers containing E-∗-E’, as mentioned above. While the trend seems rather clear for **1a**–**1e** with **7** and **2a**–**2e** with **8**, the trend seems unclear for **3a**–**3e** with **9**. This trend would be clear if the data were plotted in a narrow range, whereas it would not be clear if they were plotted in a wider range, although the mechanism is not clear:

(8) S-∗-S in 7>1d>1b>1a≥1c>>1e

(9) S-∗-Se in 8>2b>2c≈2d>2a>2e

(10) Se-∗-Se in 3c>9>3b>3a>3e>3d

(11) S-∗-S in 7>4b>4c>4d>4e >> 4a

(12) S-∗-Se in 8≈5b>5a≥5c≥5d>5e

(13) Se-∗-Se in 9>6e>6c>6b>6d>6a

In the case of S–S in **4a**–**4e** with **7**, the S-∗-S bond becomes less stable in the order shown in Equation (11). The order for the strength of S-∗-S is almost the reverse of the stability of the conformers, with the species are divided into four groups of **7**, **4b**–**4d**, **4e** and **4a**. The order for the strength of Se-∗-Se is also almost inverse, with the species divided into four groups of **9**, **6b**–**6d**, **6e** and **6a**, as shown in Equation (13). However, the trend in S-∗-Se is not as clear for **5a**–**5e** with **8**, as predicted in Equation (12). As shown in Figures S10 and S11 of the Supplementary Materials, the data for **4a**–**4e** with **7** are plotted in a narrow range, as are those for **6a**–**6e** with **9**, which seems to exhibit a clear trend. However, the data for **5a**–**5e** with **8** are plotted over a wider range, and the trend seems unclear, similar to the cases of **1a**–**3e**, although the mechanism remains unclear.

The trends shown in Equations (8)–(13) are also confirmed through the analysis of *ρ*_b_(***r***_c_) and bond orders evaluated based on the natural atomic orbitals, for E–E′ in **1a**–**6e** and **7**–**9**. The plot of *ρ*_b_(***r***_c_) versus the bond orders is shown in Figure S12 of the Supplementary Materials. See also Table S5 of the Supplementary Materials for bond orders of **1a**–**6e** and **7**–**9**.

While the results could be explained in a variety of ways, our explanation is as follows: the intramolecular attractive interactions in **1a**–**6c** stabilize the species, but E–E’ would be destabilized through the distortion. This is because the E–E’ bonds operate to relax the excess deformation brought about by intramolecular attractive interactions such as HBs. This destabilization would increase the stability of the species. As a result, the E–E’ bonds will be predicted to be less stable if they exist in more stable species. The E–E’ bonds could be predicted to be rather stable, if the intramolecular attractive interactions do not affect the structures around the E–E’ bonds.

The nature of the E–E’ bonds in **1**–**9** is well-described by applying QTAIM-DFA based on the dynamic nature with (*θ*_p_, *κ*_p_) and the static nature with (*R*, *θ*).

## 4. Conclusions

The dynamic and static nature of E–E’ (E, E’ = S and Se) in glutathione disulfide (**1**) and derivatives **2**–**3**, respectively, is elucidated by applying QTAIM-DFA together with *R*-cystine and its derivatives (**4**–**6**) and MeEE’Me (**7**–**9**). Five conformers **a**–**e** for each of **1**–**6** are optimized with M06-2X/BSS-A. The conformers are called **1a**–**1e**, which are defined to satisfy *E*(**1a**) < *E*(**1b**) < *E*(**1c**) < *E*(**1d**) < *E*(**1e**), for example. No intramolecular non-covalent interactions are detected in **7**–**9**, while many such interactions operate to stabilize **1a**–**3e**. Among such interactions, the formation of S_2_O σ(3c–4e) of the n_p_(O)→σ*(S–S) type detected in **1e** elongates *r*(S, S) by 0.20 Å. The contribution from the intramolecular non-covalent interactions to the stability of the conformers of GEE’G is estimated by separately calculating the G---G part as 2G–H and the E–E’ part (as MeE–E’Me), under suitable conditions. The *E*_rel_(2GH + MeEE’Me) values explain the *E*_rel_ values well for **1a**–**1e** and **3a**–**3e** but not so for **2a**–**2e**.

QTAIM-DFA is applied to E-∗-E’ in **1a**–**6e** and **7**–**9** by plotting *H*_b_(***r***_c_) versus *H*_b_(***r***_c_) − *V*_b_(***r***_c_)/2 for the data from the fully optimized structures and the perturbed structures at BCPs. The QTAIM-DFA parameters of (*R*, *θ*) and (*θ*_p_, *κ*_p_) are obtained by analyzing the plots. The (*θ*, *θ*_p_, *R*) values for S-∗-S in **1a**–**1e** are (188.8–189.6°, 197.2–197.6°, 0.0673–0.0744 au) for **1a**–**1d** with (181.9°, 193.8°, 0.0343 au) for **1e**. The values for **1e** are apparently smaller than those for **1a**–**1d**, due to the elongation of S-∗-S by the formation of S_2_O σ(3c–4e) in **1e**. Nevertheless, the S-∗-S interactions in **1a**–**1e** are all predicted to have the (SS/Cov_weak_) nature. Similarly, the E–E’ interactions in **2a**–**6e** and **7**–**9** are all predicted to have the (SS/Cov_weak_) nature. The S–S bonds of **1a**–**1e** are predicted to be less stable than those of **7**, and the S-∗-S bond in **1a** becomes weaker than those in **1b**, **1d** and **7**, although **1a** is the most stable among **1a**–**1e**. This inverse trend between the stability of the conformers and the strength of E-∗-E’ is widely observed. The intramolecular non-covalent attractive interactions in **1a**–**6e** stabilize the species but destabilize the E–E’ bonds through distortion, where the E–E’ bonds act to relax the excess deformation caused by the attractive interactions. These predictions of bond behavior help to understand the reactivity of E–E’ in chemical and biological processes.

## Figures and Tables

**Table 1:** Optimized *r*(E, E’) distances, torsional angles *φ*(CEE’C) (= *φ*_A_) and *E*_rel_ values for **1a**–**6e** and **7**–**9**, evaluated with M06-2X/BSS-A ^1^.

Species	*r*(E, E)	*φ*_A_	*E*_rel_	Species	*r*(E, E)	*φ*_A_	*E*_rel_
	(Å)	(°)	(kJ mol^−1^)		(Å)	(°)	(kJ mol^−1^)
**1a**	2.0736	−117.4	0.0	**2a**	2.2002	−85.6	0.0
**1b**	2.0694	−116.4	8.6	**2b**	2.1963	−110.1	1.0
**1c**	2.0778	−119.3	14.1	**2c**	2.1982	−84.5	18.0
**1d**	2.0561	100.3	29.3	**2d**	2.1959	−78.4	23.1
**1e**	2.2454	117.9	97.4	**2e**	2.2079	−65.0	23.7
**4a**	2.0625	67.7	0.0	**5a**	2.1984	−83.9	0.0
**4b**	2.0471	−82.2	0.3	**5b**	2.1890	84.3	15.7
**4c**	2.0529	88.5	0.7	**5c**	2.2011	94.0	17.5
**4d**	2.0541	−75.7	3.2	**5d**	2.2070	72.9	19.6
**4e**	2.0515	95.7	8.8	**5e**	2.2067	−66.9	27.4
**7**	2.0491	85.0	^2^	**8**	2.1923	85.6	^2^
**3a**	2.3252	−85.2	0.0				
**3b**	2.3215	−82.5	13.6				
**3c**	2.3138	−92.5	34.9				
**3d**	2.3546	−144.5	47.9				
**3e**	2.3320	105.2	58.8				
**6a**	2.3275	88.5	0.0				
**6b**	2.3303	93.4	1.4				
**6c**	2.3309	90.2	3.3				
**6d**	2.3351	74.9	3.6				
**6e**	2.3286	93.1	3.7				
**9**	2.3236	86.1	^2^				

^1^ BSS-A: The 6-311+G(3d) basis sets for S and Se with the 6-311++G(d,p) basis sets for O, N, C and H. ^2^ Not applicable.

**Table 2:** QTAIM-DFA Parameters and QTAIM Functions at BCPs for the E-∗-E′ bonds in **1a**–**3e** and **7**–**9**, ^1^ together with the frequencies (*ν*) and force constants (*k*_f_) corresponding to the E-∗-E′ species in question.

Compound	*ρ*(*r*_c_)	*c*∇^2^*ρ*_b_(*r*_c_) ^2^	*H*_b_(*r*_c_)	*R* ^3^	*θ* ^4^	*k*_b_(*r*_c_) ^5^	*ν_n_* ^6^	*k*_f_^7^	*θ*_p:NIV_^8^	*κ*_p:NIV_^9^	Classification/
(Sym: E-∗-E’)	(au)	(au)	(au)	(au)	(°)		(cm^−1^)	(mdyn Å^−1^)	(°)	(au^−1^)	Characterization
**1a** (*C*_1_: S-∗-S)	0.1378	−0.0106	−0.0676	0.0684	188.9	−2.460	493.3	0.928	197.3	0.82	SS/Cov_weak_
**1b** (*C*_1_: S-∗-S)	0.1391	−0.0113	−0.0692	0.0701	189.2	−2.483	513.8	1.718	197.3	0.78	SS/Cov_weak_
**1c** (*C*_1_: S-∗-S)	0.1368	−0.0103	−0.0665	0.0673	188.8	−2.451	489.7	0.910	197.2	0.84	SS/Cov_weak_
**1d** (*C*_1_: S-∗-S)	0.1428	−0.0124	−0.0733	0.0744	189.6	−2.512	506.0	0.917	197.6	0.70	SS/Cov_weak_
**1e** (*C*_1_: S-∗-S)	0.1025	−0.0011	−0.0345	0.0345	181.9	−2.070	353.7	0.447	193.8	3.62	SS/Cov_weak_
**2a** (*C*_1_: S-∗-Se)	0.1169	−0.0043	−0.0528	0.0530	184.7	−2.195	418.7	0.545	188.1	0.23	SS/Cov_weak_
**2b** (*C*_1_: S-∗-Se)	0.1178	−0.0046	−0.0535	0.0537	184.9	−2.206	434.0	0.955	188.4	0.15	SS/Cov_weak_
**2c** (*C*_1_: S-∗-Se)	0.1174	−0.0045	−0.0532	0.0534	184.8	−2.203	421.5	0.634	188.3	0.19	SS/Cov_weak_
**2d** (*C*_1_: S-∗-Se)	0.1176	−0.0045	−0.0532	0.0534	184.8	−2.203	404.6	0.684	188.7	0.21	SS/Cov_weak_
**2e** (*C*_1_: S-∗-Se)	0.1158	−0.0043	−0.0520	0.0522	184.7	−2.198	415.9	0.835	188.2	0.20	SS/Cov_weak_
**3a** (*C*_1_: Se-∗-Se)	0.1027	−0.0046	−0.0437	0.0440	186.0	−2.265	302.0	0.346	189.0	0.83	SS/Cov_weak_
**3b** (*C*_1_: Se-∗-Se)	0.1035	−0.0048	−0.0444	0.0446	186.2	−2.275	310.2	0.485	189.2	0.78	SS/Cov_weak_
**3c** (*C*_1_: Se-∗-Se)	0.1048	−0.0050	−0.0458	0.0461	186.3	−2.282	316.0	0.423	189.2	0.84	SS/Cov_weak_
**3d** (*C*_1_: Se-∗-Se)	0.0988	−0.0046	−0.0406	0.0409	186.4	−2.291	304.2	0.684	189.8	1.31	SS/Cov_weak_
**3e** (*C*_1_: Se-∗-Se)	0.1022	−0.0045	−0.0435	0.0437	185.9	−2.259	300.6	0.835	189.4	0.99	SS/Cov_weak_
**7** (*C*_2_: S-∗-S)	0.1446	−0.0131	−0.0751	0.0763	189.9	−2.535	513.7	2.645	197.6	0.66	SS/Cov_weak_
**8** (*C*_1_: S-∗-Se)	0.1189	−0.0048	−0.0544	0.0547	185.0	−2.213	419.7	2.072	188.6	0.38	SS/Cov_weak_
**9** (*C*_2_: Se-∗-Se)	0.1036	−0.0050	−0.0445	0.0448	186.4	−2.291	307.7	2.730	189.1	0.77	SS/Cov_weak_

^1^ 6-311+G(3d) basis sets employed for S and Se with the 6-311++G(d,p) basis sets for O, N, C and H at the DFT level of M06-2X. Frequencies and force constants related to the NIV to generate the perturbed structures are also listed. ^2^ *c*∇^2^*ρ*_b_(***r***_c_) = *H*_b_(***r***_c_) − *V*_b_(***r***_c_)/2 where c = *ħ*^2^/8*m*. ^3^ *R* = [(*H*_b_(***r***_c_) − *V*_b_(***r***_c_)/2)^2^ + *H*_b_(***r***_c_)^2^]^1/2^. ^4^ *θ* = 90° − tan^−1^[*H*_b_(***r***_c_)/(*H*_b_(***r***_c_) − *V*_b_(***r***_c_)/2)]. ^5^ *k*_b_(***r***_c_) = *V*_b_(***r***_c_)/*G*_b_(***r***_c_). ^6^ Frequency corresponding to the stretching mode of the E-∗-E’ bond, where ∗ means the bond critical point in question. ^7^ Force constants correspond to *ν_n_*. ^8^ *θ*_p_ = 90° − tan^−1^(d*y*/d*x*), where (*x*, *y*) = (*H*_b_(***r***_c_) − *V*_b_(***r***_c_)/2, *H*_b_(***r***_c_)). ^9^ *κ*_p_ = |d^2^*y*/d*x*^2^|/[1 + (dy/dx)2]^3/2^.

## Supplementary Materials

The following are available online at http://www.mdpi.com/1420-3049/23/2/443/s1, Scheme S1: QTAIM-DFA: Plot of *H*_b_(***r***_c_) versus *H*_b_(***r***_c_) − *V*_b_(***r***_c_)/2 for weak to strong interactions, Scheme S2: Rough classification of interactions by *θ* and *θ*_p_, together with *k*_b_(***r***_c_) (= *V*_b_(***r***_c_)/*G*_b_(***r***_c_)), Equations (S1)–(S7), Table S1: The energies for **7**–**9** of the optimized structures and partially optimized structures with *φ*_A_, fixed suitably, with M06-2X/BSS-A, Table S2: Number of the O–H---O (Int A), O–H---N, N–H---O (Int B), N–H---N (Int C), HBs with the E(E’)---H–O(N) and E(E’)---C = O (Int D) and E(E’)---O = C and E(E’)---NH–C = O (Int E) interactions in **1**–**6**, evaluated with M06-2X/BSS-A, Table S3: The relative energies (*E*_rel_) of REE’R, (R–H + R–H) and MeE–E’Me for **4a**–**6e**, evaluated with M06-2X/BSS-A, Table S4: QTAIM-DFA parameters and QTAIM functions at BCPs for the E–E’ bonds in **4a**–**6e** and **7**–**9**, *^a^* together with the frequencies (*ν*) and force constants (*k*_f_), corresponding to the E-∗-E′ bonds in question, Table S5: NAO bond orders for **1a**–**6e** and **7**–**8**, Figure S1: Polar (*R*, *θ*) coordinate representation of *H_b_*(***r***_c_) versus *H_b_*(***r***_c_) − *V_b_*(***r***_c_)/2, with (*θ*_p_, *κ*_p_) parameters, Figure S2: Molecular graphs of **2b**–**2e**, drawn on the optimized structures, Figure S3: Molecular graphs of **3b**–**3e**, drawn on the optimized structures, Figure S4: Molecular graphs of **5b**–**5e**, drawn on the optimized structures, Figure S5: Molecular graphs of **6b**–**6e**, drawn on the optimized structures, Figure S6: Molecular graphs of **7**–**9**, drawn on the optimized structures, Figure S7: Plots of *E*_rel_ of REE’R and (2R–H + MeEE’Me) for **4a**–**e** (CysSSCys), **5a**–**e** (CysSSeCys) and **6a**–**e** (CysSeSeCys), evaluated with M06-2X/BSS-A, Figure S8: Trajectory plots of *ρ*_b_(***r***_c_) drawn on the S–S–C planes of **1a**–**1e**, similarly to the case of Figure 6 in the text. Color and marks are same as those in Figure 6, Figure S9: Plots of *H*_b_(***r***_c_) versus *H*_b_(***r***_c_) − *V*_b_(***r***_c_)/2 for **1a**–**3e** and **7**–**9**, Figure S10: Plots of *H*_b_(***r***_c_) versus *H*_b_(***r***_c_) – *V*_b_(***r***_c_)/2 for **4a**–**4e** and **7**, Figure S11: Plots of *H*_b_(***r***_c_) versus *H*_b_(***r***_c_) − *V*_b_(***r***_c_)/2 for **5a**–**6e** and **7**–**8**, Figure S12: Plots of *ρ*_b_(***r***_c_) versus NAO bond orders for **1a**–**6e** and **7**–**8** and Cartesian coordinates and energies of all the species involved in the present work.
