# Supplementary material for: Behavior of the E–E’ Bonds (E, E’ = S and Se) in Glutathione Disulfide and Derivatives Elucidated by Quantum Chemical Calculations with the Quantum Theory of Atoms-In-Molecules Approach
Source: Molecules. 2018 Feb 17;23(2):443. doi: 10.3390/molecules23020443 (PMC6017556; doi:10.3390/molecules23020443)
Supplement: Supplementary file 1 [file molecules-23-00443-s001.pdf]

Electronic Supplementary Information

Behavior of the E–E' Bonds (E, E' = S and Se) in Glutathione Disulfide and Derivatives Elucidated by QC Calculations with QTAIM Approach

Satoko Hayashi,* Yutaka Tsubomoto and Waro Nakanishi*

Faculty of Systems Engineering, Wakayama University, 930 Sakaedani, Wakayama 640-8510, Japan. Fax: +81 73 457 8253; Tel: +81 73 457 8252; E-mail: hayashi3@sys.wakayama-u.ac.jp and nakanisi@sys.wakayama-u.ac.jp.

Table of Contents **Pages**

QTAIM Dual Functional Analysis (QTAIM-DFA) 2–4
Criteria for Classification of Interactions: Behavior of Typical Interactions Elucidated by QTAIM-DFA 4
Additional figures and tables 5–16
Table S1 The energies for **7**–**9** of the optimized structures and partially optimized structures with ϕ_A, fixed suitably, with M06-2X/BSS-A 5
Table S2 Number of the O–H---O (Int A), O–H---N, N–H---O (Int B), N–H---N (Int C), HBs with the E(E')---H–O(N) and E(E')---C=O (Int D) and E(E')---O=C and E(E')---NH–C=O (Int E) interactions in **1**–**6**, evaluated with M06-2X/BSS-A 5
Table S3 The relative energies (E_{rel}) of REE'R, (R–H + R–H) and MeE–E'Me for **4a**–**6e**, evaluated with M06-2X/BSS-A 6
Table S4 QTAIM-DFA parameters and QTAIM functions at BCPs for the E–E' bonds in **4a**–**6e** and **7**–**9**, together with the frequencies (ν) and force constants (k_f), corresponding to the E-*-E′ bonds in question 7
Table S5 NAO bond orders for **1a**–**6e** and **7**–**8** 8
Fig. S2 Molecular graphs of **2b**–**2e**, drawn on the optimized structures. 9
Fig. S3 Molecular graphs of **3b**–**3e**, drawn on the optimized structures. 10
Fig. S4 Molecular graphs of **5b**–**5e**, drawn on the optimized structures. 11
Fig. S5 Molecular graphs of **6b**–**6e**, drawn on the optimized structures. 11
Fig. S6 Molecular graphs of **7**–**9**, drawn on the optimized structures. 12
Fig. S7 Plots of E_{rel} of REE'R and (2R–H + MeEE'Me) for **4a**–**4e** (CysSSCys), **5a**–**5e** (CysSSeCys) and **6a**–**6e** (CysSeSeCys), evaluated with M06-2X/BSS-A. 12
Fig. S8 Trajectory plots of $\rho_b(\boldsymbol{r}_c)$ drawn on the S–S–C planes of **1a**–**1e**, similarly to the case of Fig. 6 in the text. Color and marks are same as those in Fig. 6. 13
Fig. S9 Plots of $H_b(\boldsymbol{r}_c)$ versus $H_b(\boldsymbol{r}_c) - V_b(\boldsymbol{r}_c)/2$ for **1a**–**3e** and **7**–**9**. 14
Fig. S10 Plots of $H_b(\boldsymbol{r}_c)$ versus $H_b(\boldsymbol{r}_c) - V_b(\boldsymbol{r}_c)/2$ for **4a**–**4e** and **7**. 15
Fig. S11 Plots of $H_b(\boldsymbol{r}_c)$ versus $H_b(\boldsymbol{r}_c) - V_b(\boldsymbol{r}_c)/2$ for **5a**–**6e** and **7**–**8**. 15
Fig. S12 Plots of $\rho_b(\boldsymbol{r}_c)$ versus NAO bond orders for **1a**–**6e** and **7**–**8**. 16
References 16
Optimized structures given by Cartesian coordinates 17–50

QTAIM Dual Functional Analysis (QTAIM-DFA)

The bond critical point (BCP; ∗) is an important concept in QTAIM. The BCP of $(\omega, \sigma) = (3, -1)$[S1] is a point along the bond path (BP) at the interatomic surface, where charge density $\rho(\boldsymbol{r})$ reaches a minimum. It is donated by $\rho_b(\boldsymbol{r}_c)$. While the chemical bonds or interactions between A and B are denoted by A–B, which correspond to BPs between A and B in QTAIM, A-∗-B emphasizes the presence of BCP (∗) in A–B.

The sign of the Laplacian $\rho_b(\boldsymbol{r}_c)$ ($\nabla^2\rho_b(\boldsymbol{r}_c)$) indicates that $\rho_b(\boldsymbol{r}_c)$ is depleted or concentrated with respect to its surrounding, since $\nabla^2\rho_b(\boldsymbol{r}_c)$ is the second derivative of $\rho_b(\boldsymbol{r}_c)$. $\rho_b(\boldsymbol{r}_c)$ is locally depleted relative to the average distribution around $\boldsymbol{r}_c$ if $\nabla^2\rho_b(\boldsymbol{r}_c) > 0$, but it is concentrated when $\nabla^2\rho_b(\boldsymbol{r}_c) < 0$. Total electron energy densities at BCPs ($H_b(\boldsymbol{r}_c)$) must be a more appropriate measure for weak interactions on the energy basis.[S1–S6] $H_b(\boldsymbol{r}_c)$ are the sum of kinetic energy densities ($G_b(\boldsymbol{r}_c)$) and potential energy densities ($V_b(\boldsymbol{r}_c)$) at BCPs, as shown in eqn (S1). Electrons at BCPs are stabilized when $H_b(\boldsymbol{r}_c) < 0$, therefore, interactions exhibit the covalent nature in this region, whereas they exhibit no covalency if $H_b(\boldsymbol{r}_c) > 0$, due to the destabilization of electrons at BCPs under the conditions.[S1] Eqn (S2) represents the relation between $\nabla^2\rho_b(\boldsymbol{r}_c)$ and $H_b(\boldsymbol{r}_c)$, together with $G_b(\boldsymbol{r}_c)$ and $V_b(\boldsymbol{r}_c)$, which is closely related to the virial theorem.

$$H_b(\boldsymbol{r}_c) = G_b(\boldsymbol{r}_c) + V_b(\boldsymbol{r}_c) \tag{S1}$$
$$(\hbar^2/8m)\nabla^2\rho_b(\boldsymbol{r}_c) = H_b(\boldsymbol{r}_c) - V_b(\boldsymbol{r}_c)/2 \tag{S2}$$
$$= G_b(\boldsymbol{r}_c) + V_b(\boldsymbol{r}_c)/2 \tag{S2'}$$

Interactions are classified by the signs of $\nabla^2\rho_b(\boldsymbol{r}_c)$ and $H_b(\boldsymbol{r}_c)$. Interactions in the region of $\nabla^2\rho_b(\boldsymbol{r}_c) < 0$ are called shared-shell (SS) interactions and they are closed-shell (CS) interactions for $\nabla^2\rho_b(\boldsymbol{r}_c) > 0$. $H_b(\boldsymbol{r}_c)$ must be negative when $\nabla^2\rho_b(\boldsymbol{r}_c) < 0$, since $H_b(\boldsymbol{r}_c)$ are larger than $(\hbar^2/8m)\nabla^2\rho_b(\boldsymbol{r}_c)$ by $V_b(\boldsymbol{r}_c)/2$ with negative $V_b(\boldsymbol{r}_c)$ at all BCPs (eqn (S2)). Consequently, $\nabla^2\rho_b(\boldsymbol{r}_c) < 0$ and $H_b(\boldsymbol{r}_c) < 0$ for the SS interactions. The CS interactions are especially called *pure* CS interactions for $H_b(\boldsymbol{r}_c) > 0$ and $\nabla^2\rho_b(\boldsymbol{r}_c) > 0$, since electrons at BCPs are depleted and destabilized under the conditions.[S1] Electrons in the intermediate region between SS and *pure* CS, which belong to CS, are locally depleted but stabilized at BCPs, since $\nabla^2\rho_b(\boldsymbol{r}_c) > 0$ but $H_b(\boldsymbol{r}_c) < 0$.[S1] We call the interactions in this region *regular* CS,[S4,S5] when it is necessary to distinguish from *pure* CS. The role of $\nabla^2\rho_b(\boldsymbol{r}_c)$ in the classification can be replaced by $H_b(\boldsymbol{r}_c) - V_b(\boldsymbol{r}_c)/2$, since $(\hbar^2/8m)\nabla^2\rho_b(\boldsymbol{r}_c) = H_b(\boldsymbol{r}_c) - V_b(\boldsymbol{r}_c)/2$ (eqn (S2)).

We proposed QTAIM-DFA by plotting $H_b(\boldsymbol{r}_c)$ versus $H_b(\boldsymbol{r}_c) - V_b(\boldsymbol{r}_c)/2$ (= $(\hbar^2/8m)\nabla^2\rho_b(\boldsymbol{r}_c)$),[S4] after the proposal of $H_b(\boldsymbol{r}_c)$ versus $\nabla^2\rho_b(\boldsymbol{r}_c)$.[S4] Both axes in the plot of the former are given in energy unit, therefore, distances on the (x, y) (= $(H_b(\boldsymbol{r}_c) - V_b(\boldsymbol{r}_c)/2, H_b(\boldsymbol{r}_c))$) plane can be expressed in the energy unit, which provides an analytical development. QTAIM-DFA can incorporate the classification of interactions by the signs of $\nabla^2\rho_b(\boldsymbol{r}_c)$ and $H_b(\boldsymbol{r}_c)$. Scheme S1 summarizes the QTAIM-DFA treatment. Interactions of *pure* CS appear in the first quadrant, those of *regular* CS in the forth quadrant and SS interactions do in the third quadrant. No interactions appear in the second one.

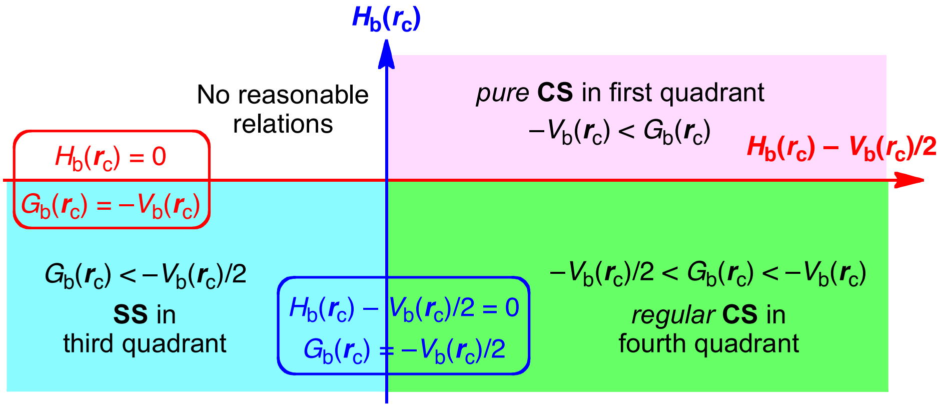

Scheme S1. QTAIM-DFA: Plot of $H_b(\boldsymbol{r}_c)$ versus $H_b(\boldsymbol{r}_c) - V_b(\boldsymbol{r}_c)/2$ for weak to strong interactions.

In our treatment, data for perturbed structures around fully optimized structures are also employed for the plots, together with the fully optimized ones (see Figure S1).[S4-S6] We proposed the concept of the "dynamic nature of interaction" originated from the perturbed structures. The behavior of interactions at the fully optimized structures corresponds to "the static nature of interactions", whereas that containing perturbed structures exhibit the "dynamic nature of interaction" as explained below. The method to generate the perturbed structures is discussed later. Plots of $H_b(\boldsymbol{r}_c)$ versus $H_b(\boldsymbol{r}_c) - V_b(\boldsymbol{r}_c)/2$ are analyzed employing the polar coordinate (R, θ) representation with (θ_p, κ_p) parameters.[S4-S6] Figure S1 explains the treatment. R in (R, θ) is defined by eqn (S3) and given in the energy unit. R corresponds to the energy for an interaction at BCP. The plots show a spiral stream, as a whole. θ in (R, θ) defined by eqn (S4), measured from the y-axis, controls the spiral stream of the plot. Each plot for an interaction shows a specific curve, which provides important information of the interaction (see Figure S1). The curve is expressed by θ_p and κ_p. While θ_p, defined by eqn (S5) and measured from the y-direction, corresponds to the tangent line of a plot, where θ_p is calculated employing data of the perturbed structures with a fully-optimized structure and κ_p is the curvature of the plot (eqn (S6)). While (R, θ) correspond to the static nature, (θ_p, κ_p) represent the dynamic nature of interactions. We call (R, θ) and (θ_p, κ_p) QTAIM-DFA parameters, whereas $\rho_b(\boldsymbol{r}_c)$, $\nabla^2\rho_b(\boldsymbol{r}_c)$, $G_b(\boldsymbol{r}_c)$, $V_b(\boldsymbol{r}_c)$, $H_b(\boldsymbol{r}_c)$ and $H_b(\boldsymbol{r}_c) - V_b(\boldsymbol{r}_c)/2$ belong to QTAIM functions. $k_b(\boldsymbol{r}_c)$, defined by eqn (S7), is a QTAIM function but it will be treated as if it were a QTAIM-DFA parameter, if suitable.

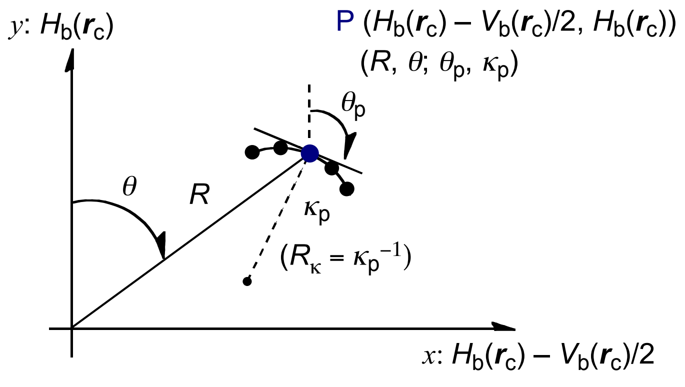

Fig. S1 Polar (R, θ) coordinate representation of $H_b(\boldsymbol{r}_c)$ versus $H_b(\boldsymbol{r}_c) - V_b(\boldsymbol{r}_c)/2$, with (θ_p, κ_p) parameters.

$R = (x^2 + y^2)^{1/2}$ (S3)

$\theta = 90° - \tan^{-1}(y/x)$ (S4)

$\theta_p = 90° - \tan^{-1}(dy/dx)$ (S5)

$\kappa_p = |d^2y/dx^2| / [1 + (dy/dx)^2]^{3/2}$ (S6)

$k_b(\boldsymbol{r}_c) = V_b(\boldsymbol{r}_c)/G_b(\boldsymbol{r}_c)$ (S7)

where $(x, y) = (H_b(\boldsymbol{r}_c) - V_b(\boldsymbol{r}_c)/2, H_b(\boldsymbol{r}_c))$

Criteria for Classification of Interactions: Behavior of Typical Interactions Elucidated by QTAIM-DFA

$H_b(\boldsymbol{r}_c)$ are plotted versus $H_b(\boldsymbol{r}_c) - V_b(\boldsymbol{r}_c)/2$ for typical interactions in vdW (van der Waals interactions), HB (hydrogen bonds), CT-MC (molecular complexes through charge transfer), X_3^- (trihalide ions), CT-TBP (trigonal bipyramidal adducts through charge-transfer), Cov_{weak} (weak covalent bonds) and Cov_{strong} (strong covalent bonds).[S4-S6] Rough criteria are obtained, after the analysis of the plots for the typical interactions according to eqns (S3)–(S7), by applying QTAIM-DFA. Scheme S2 shows the rough criteria, which are accomplished by the θ and θ_p values, together with the values of $k_b(\boldsymbol{r}_c)$. The criteria will be employed to discuss the nature of interactions in question, as a reference.

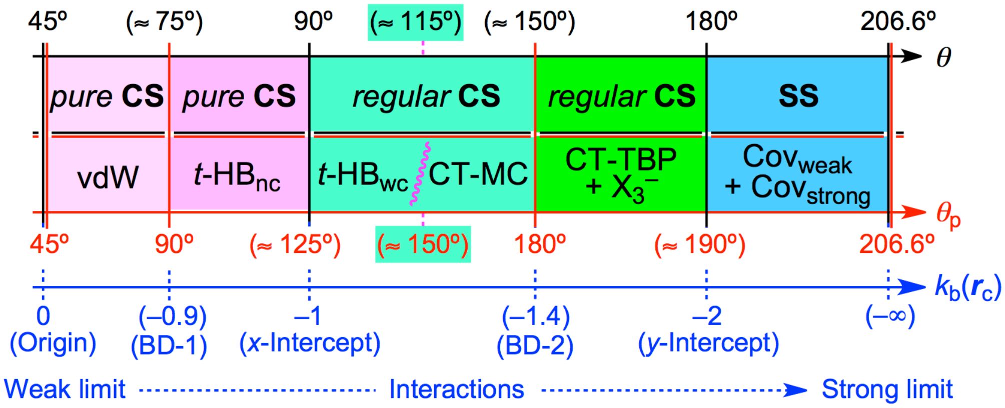

Scheme S2. Rough classification of interactions by θ and θ_p, together with $k_b(\boldsymbol{r}_c)$ (= $V_b(\boldsymbol{r}_c)/G_b(\boldsymbol{r}_c)$).

Table S1. The energies for **7**–**9** of the optimized structures and partially optimized structures with ϕ_A, fixed suitably, with M06-2X/BSS-A.

dihedral (°)	ΔE (kJ mol^{-1})	dihedral (°)	ΔE (kJ mol^{-1})
**7**		**9**	
0.00	44.72	0.00	35.96
84.96	0.00[a]	15.00	31.97
180.00	25.71	30.00	22.99
**8**		45.00	13.07
0.00	38.95	60.00	5.57
85.59	0.00[a]	75.00	0.62
180.00	23.62	86.08	0.00[a]
		90.00	0.10
		105.00	2.51
		120.00	7.86
		135.00	13.73
		150.00	18.80
		165.00	22.17
		180.00	23.11

[a] Taken as the reference (0.0 kJ mol^{-1}).

Table S2. Number of the O–H---O (Int A), O–H---N, N–H---O (Int B), N–H---N (Int C), HBs with the E(E')---H–O(N) and E(E')---C=O (Int D) and E(E')---O=C and E(E')---NH–C=O (Int E) interactions in **1**–**6**, evaluated with M06-2X/BSS-A.[a]

Species	Int A	Int B	Int C	Int D	Int E	Species	Int A	Int B	Int C	Int D	Int E
**1a**	2	10	0	0	1	**2a**	2	9	0	1	1
**1b**	3	6	1	0	1	**2b**	2	9	0	0	1
**1c**	2	7	0	0	1	**2c**	2	7	0	0	1
**1d**	3	6	1	1	3	**2d**	2	6	0	1	1
**1e**	2	8	0	0	2	**2e**	2	8	0	0	1
**3a**	3	6	1	0	2	**4a**	1	2	0	1	0
**3b**	3	6	1	0	2	**4b**	0	2	0	0	0
**3c**	4	4	1	1	3	**4c**	0	1	1	0	0
**3d**	1	9	0	1	2	**4d**	1	1	0	0	0
**3e**	2	9	0	1	4	**4e**	1	0	0	0	0
**5a**	1	1	0	0	0	**6a**	0	1	0	0	1
**5b**	2	0	0	1	0	**6b**	1	1	0	1	0
**5c**	1	1	0	1	0	**6c**	0	2	0	1	1
**5d**	0	1	0	1	0	**6d**	0	1	0	1	1
**5e**	0	4	0	0	0	**6e**	1	0	0	0	1

[a] BSS-A: The 6-311+G(3d) basis sets for S and Se with the 6-311++G(d,p) basis sets for O, N, C and H. [b] Not applicable.

Table S3 The relative energies (E_{rel}) of REE'R, (R–H + R–H) and MeE–E'Me for **1a**–**6e**, evaluated with M06-2X/BSS-A.[a,b]

Conformer	a	b	c	d	e
**1** (E, E') = (S, S)					
E_{rel}(GSSG)$_{opt}$	0.0	8.6	14.1	29.3	97.4
E_{rel}(2GH)$_{p-opt}$	0.0	11.8	14.4	59.2	83.2
E_{rel}(MeSSMe)$_{p-opt}$	0.0[c]	–2.4	1.5	–9.9	27.1[d]
E_{rel}(2GH+MeSSMe)$_{p-opt}$	0.0	9.4	15.9	49.3	110.4[d]
**2** (E, E') = (S, Se)					
E_{rel}(GSSeG)$_{opt}$	0.0	1.0	18.0	23.1	23.7
E_{rel}(2GH)$_{p-opt}$	0.0	–22.7	16.9	4.1	16.1
E_{rel}(MeSSeMe)$_{p-opt}$	0.0[e]	4.3	-0.5	4.3	2.2
E_{rel}(2GH+MeSSeMe)$_{p-opt}$	0.0	–18.4	16.4	8.4	18.3
**3** (E, E') = (Se, Se)					
E_{rel}(GSeSeG)$_{opt}$	0.0	13.6	34.9	47.9	58.8
E_{rel}(2GH)$_{p-opt}$	0.0	11.5	32.8	27.3	54.7
E_{rel}(MeSeSeMe)$_{p-opt}$	0.0[f]	–2.1	-2.4	15.2	3.5
E_{rel}(2GH+MeSeSeMe)$_{p-opt}$	0.0	9.3	30.4	42.4	58.1
**4** (E, E') = (S, S)					
E_{rel}(RSSR)$_{opt}$	0.0	0.3	0.7	3.2	8.8
E_{rel}(2RH)$_{p-opt}$	0.0	18.6	6.0	18.2	9.8
E_{rel}(MeSSMe)$_{p-opt}$	0.0[g]	–0.9	–2.0	–2.3	–0.6
E_{rel}(2RH+MeSSMe)$_{p-opt}$	0.0	17.7	4.0	15.8	9.2
**5** (E, E') = (S, Se)					
E_{rel}(RSSeR)$_{opt}$	0.0	15.7	17.5	19.6	27.4
E_{rel}(2RH)$_{p-opt}$	0.0	–3.3	11.7	24.4	12.5
E_{rel}(MeSSeMe)$_{p-opt}$	0.0[h]	0.7	0.8	0.7	3.2
E_{rel}(2RH+MeSSeMe)$_{p-opt}$	0.0	–2.5	12.6	25.1	15.7
**6** (E, E') = (Se, Se)					
E_{rel}(RSeSeR)$_{opt}$	0.0	1.4	3.3	3.6	3.7
E_{rel}(2RH)$_{p-opt}$	0.0	25.5	20.6	22.6	3.7
E_{rel}(MeSeSeMe)$_{p-opt}$	0.0[i]	–2.9	–4.1	–3.3	–4.0
E_{rel}(2RH+MeSeSeMe)$_{p-opt}$	0.0	22.6	16.5	19.3	–0.3

[a] BSS-A: The 6-311+G(3d) basis sets for S and Se with the 6-311++G(d,p) basis sets for O, N, C and H. [b] In kJ mol^{-1}. [c] Less stable than the fully optimized MeSSMe by 13.7 kJ mol^{-1}. [d] The n(O)→σ*(S–S) 3c–4e interaction is predicted to stabilize the system by 20.5 kJ mol^{-1}. [e] Less stable than the fully optimized MeSSeMe by 2.5 kJ mol^{-1}. [f] Less stable than the fully optimized MeSeSeMe by 4.2 kJ mol^{-1}. [g] Less stable than the fully optimized MeSSMe by 4.2 kJ mol^{-1}. [h] Less stable than the fully optimized MeSSeMe by 1.6 kJ mol^{-1}. [i] Less stable than the fully optimized MeSSMe by 5.5 kJ mol^{-1}.

Table S4 QTAIM-DFA parameters and QTAIM functions at BCPs for the E–E' bonds in **4a**–**6e** and **7**–**9**,[a] together with the frequencies (ν) and force constants (k_f), corresponding to the E-∗-E′ bonds in question.

Compound (symm: E-∗-E')	$\rho_b(\boldsymbol{r}_c)$ (au)	$c\nabla^2\rho_b(\boldsymbol{r}_c)$[b] (au)	$H_b(\boldsymbol{r}_c)$ (au)	R[c] (au)	θ[d] (°)	$k_b(\boldsymbol{r}_c)$[e]	ν_n[f] (cm^{-1})	k_f[g] (unit[h])	$\theta_{p:NIV}$[i] (°)	$\kappa_{p:NIV}$[j] (au^{-1})	Classific/ Charac[k]
**4a** (C_1: S-∗-S)	0.1409	–0.0117	–0.0710	0.0719	189.4	–2.495	511.3	2.060	197.5	0.75	SS/Cov_{weak}
**4b** (C_1: S-∗-S)	0.1443	–0.0126	–0.0749	0.0759	189.6	–2.509	522.5	1.118	197.4	0.67	SS/Cov_{weak}
**4c** (C_1: S-∗-S)	0.1432	–0.0124	–0.0737	0.0747	189.6	–2.508	506.8	1.947	197.5	0.69	SS/Cov_{weak}
**4d** (C_1: S-∗-S)	0.1431	–0.0124	–0.0735	0.0746	189.5	–2.506	514.1	2.089	197.4	0.71	SS/Cov_{weak}
**4e** (C_1: S-∗-S)	0.1430	–0.0122	–0.0734	0.0744	189.4	–2.495	519.5	1.508	197.4	0.70	SS/Cov_{weak}
**5a** (C_1: S-∗-Se)	0.1171	–0.0041	–0.0529	0.0531	184.4	–2.183	414.4	0.551	188.0	0.34	SS/Cov_{weak}
**5b** (C_1: S-∗-Se)	0.1188	–0.0045	–0.0547	0.0548	184.7	–2.195	423.6	1.896	188.4	0.33	SS/Cov_{weak}
**5c** (C_1: S-∗-Se)	0.1166	–0.0040	–0.0525	0.0527	184.4	–2.180	414.7	1.440	188.2	0.40	SS/Cov_{weak}
**5d** (C_1: S-∗-Se)	0.1157	–0.0036	–0.0524	0.0525	184.0	–2.161	413.8	1.996	187.5	0.34	SS/Cov_{weak}
**5e** (C_1: S-∗-Se)	0.1163	–0.0048	–0.0517	0.0519	185.3	–2.225	413.1	1.278	189.5	0.03	SS/Cov_{weak}
**6a** (C_1: Se-∗-Se)	0.1020	–0.0042	–0.0431	0.0433	185.6	–2.242	301.9	1.875	188.9	0.71	SS/Cov_{weak}
**6b** (C_1: Se-∗-Se)	0.1021	–0.0046	–0.0432	0.0434	186.0	–2.268	308.0	0.174	189.3	0.77	SS/Cov_{weak}
**6c** (C_1: Se-∗-Se)	0.1023	–0.0046	–0.0435	0.0437	186.1	–2.269	298.7	0.938	189.4	0.68	SS/Cov_{weak}
**6d** (C_1: Se-∗-Se)	0.1017	–0.0044	–0.0431	0.0433	185.9	–2.259	308.7	0.582	189.1	0.80	SS/Cov_{weak}
**6e** (C_1: Se-∗-Se)	0.1027	–0.0047	–0.0437	0.0439	186.1	–2.271	306.2	0.172	188.9	0.93	SS/Cov_{weak}
**7** (C_2: S-∗-S)	0.1446	–0.0131	–0.0751	0.0763	189.9	–2.535	513.7	2.645	197.6	0.66	SS/Cov_{weak}
**8** (C_2: S-∗-Se)	0.1189	–0.0048	–0.0544	0.0547	185.0	–2.213	419.7	2.072	188.6	0.38	SS/Cov_{weak}
**9** (C_2: Se-∗-Se)	0.1036	–0.0050	–0.0445	0.0448	186.4	–2.291	307.7	2.730	189.1	0.77	SS/Cov_{weak}

[a] The 6-311+G(3d) basis sets being employed for S and Se with the 6-311++G(d,p) basis sets for O, N, C and H at the DFT level of M06-2X. The frequencies and force constant related to NIV to generate the perturbed structures are also contained. [b] $c\nabla^2\rho_b(\boldsymbol{r}_c) = H_b(\boldsymbol{r}_c) - V_b(\boldsymbol{r}_c)/2$ where $c = \hbar^2/8m$. [c] $R = [(H_b(\boldsymbol{r}_c) - V_b(\boldsymbol{r}_c)/2)^2 + H_b(\boldsymbol{r}_c)^2]^{1/2}$. [d] $\theta = 90° - \tan^{-1}[H_b(\boldsymbol{r}_c)/(H_b(\boldsymbol{r}_c) - V_b(\boldsymbol{r}_c)/2)]$. [e] $k_b(\boldsymbol{r}_c) = V_b(\boldsymbol{r}_c)/G_b(\boldsymbol{r}_c)$. [f] Frequency corresponding to the stretching mode of the E-∗-E' bond, where ∗ means the bond critical point in question. [g] Force constants correspond to ν_n. [h] mdyn $Å^{-1}$. [i] $\theta_p = 90° - \tan^{-1}(dy/dx)$ where $(x, y) = (H_b(\boldsymbol{r}_c) - V_b(\boldsymbol{r}_c)/2, H_b(\boldsymbol{r}_c))$. [j] $\kappa_p = |d^2y/dx^2|/[1 + (dy/dx)^2]^{3/2}$. [k] Classification/Characterization.

Table S5 NAO bond orders for **1a**–**6e** and **7**–**8**.

Compound	NAO bond order[a,b]
**1a**	0.8177
**1b**	0.8101
**1c**	0.8134
**1d**	0.8233
**1e**	0.6928
**2a**	0.7641
**2b**	0.7709
**2c**	0.7650
**2d**	0.7792
**2e**	0.7526
**3a**	0.7493
**3b**	0.7465
**3c**	0.7383
**3d**	0.6762
**3e**	0.7219
**4a**	0.8275
**4b**	0.8565
**4c**	0.8402
**4d**	0.8395
**4e**	0.8484
**5a**	0.7701
**5b**	0.7863
**5c**	0.7642
**5d**	0.7543
**5e**	0.7618
**6a**	0.745
**6b**	0.7333
**6c**	0.7296
**6d**	0.7275
**6e**	0.7391
**7**	0.8481
**8**	0.7799
**9**	0.7442

[a] Atom-atom overlap-weighted NAO bond order. [b] The orders evaluated based on the natural atomic orbitals using NBO 6.0 program.

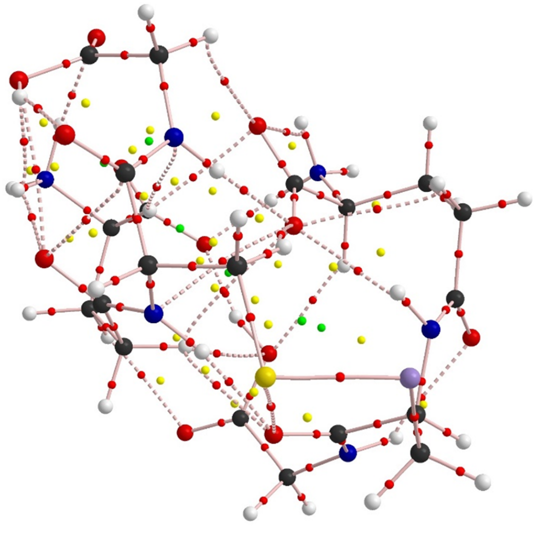

2b (E_{rel} = 1.0 kJ mol^{-1})

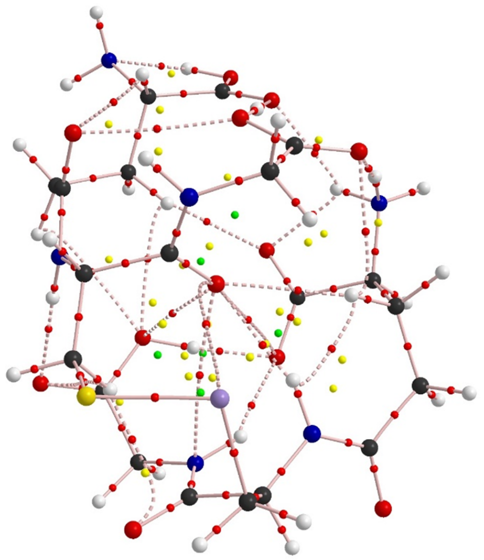

2c (E_{rel} = 18.0 kJ mol^{-1})

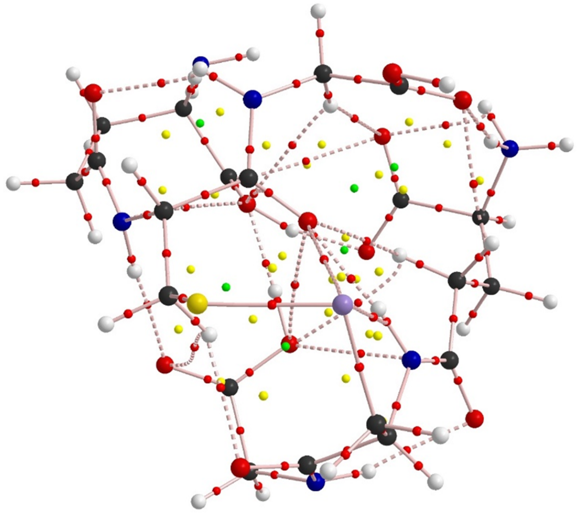

2d (E_{rel} = 23.1 kJ mol^{-1})

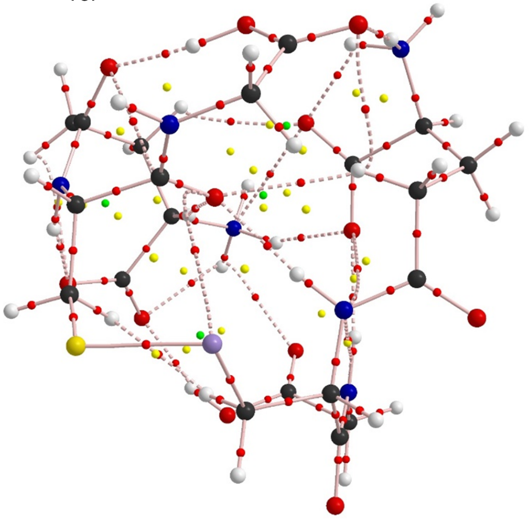

2e (E_{rel} = 23.7 kJ mol^{-1})

Fig. S2 Molecular graphs of **2b**–**2e**, drawn on the optimized structures.

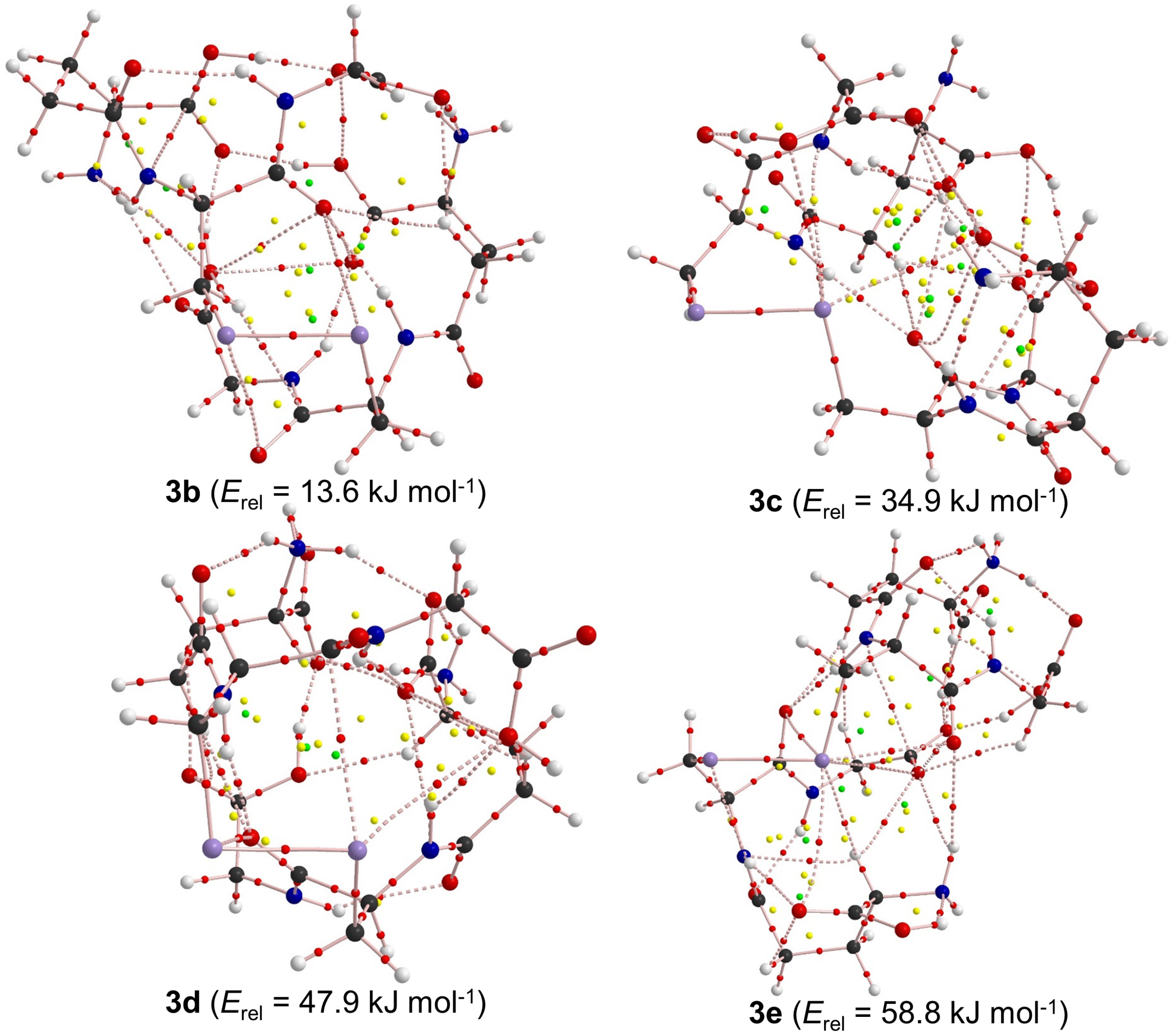

Fig. S3 Molecular graphs of **3b**–**3e**, drawn on the optimized structures.

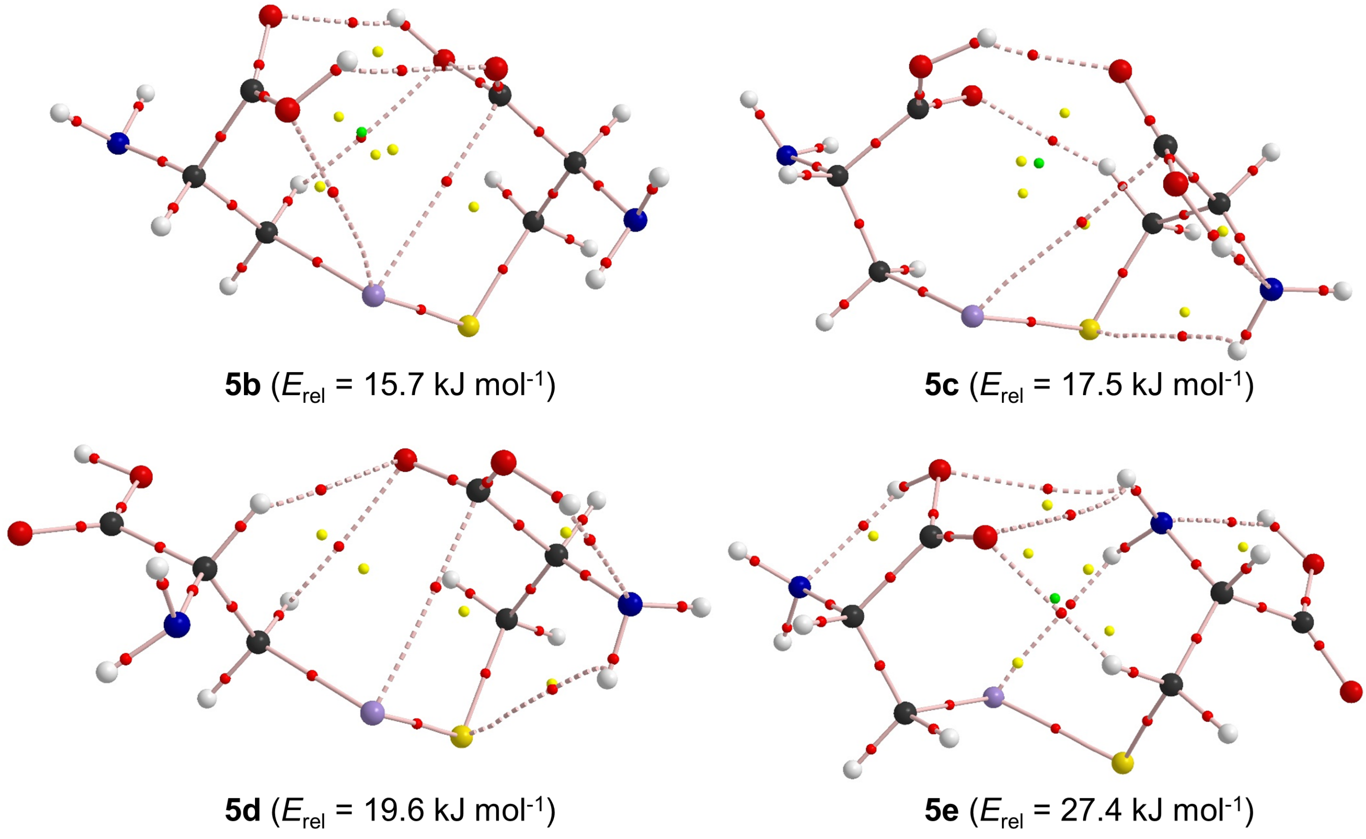

Fig. S4 Molecular graphs of **5b**–**5e**, drawn on the optimized structures.

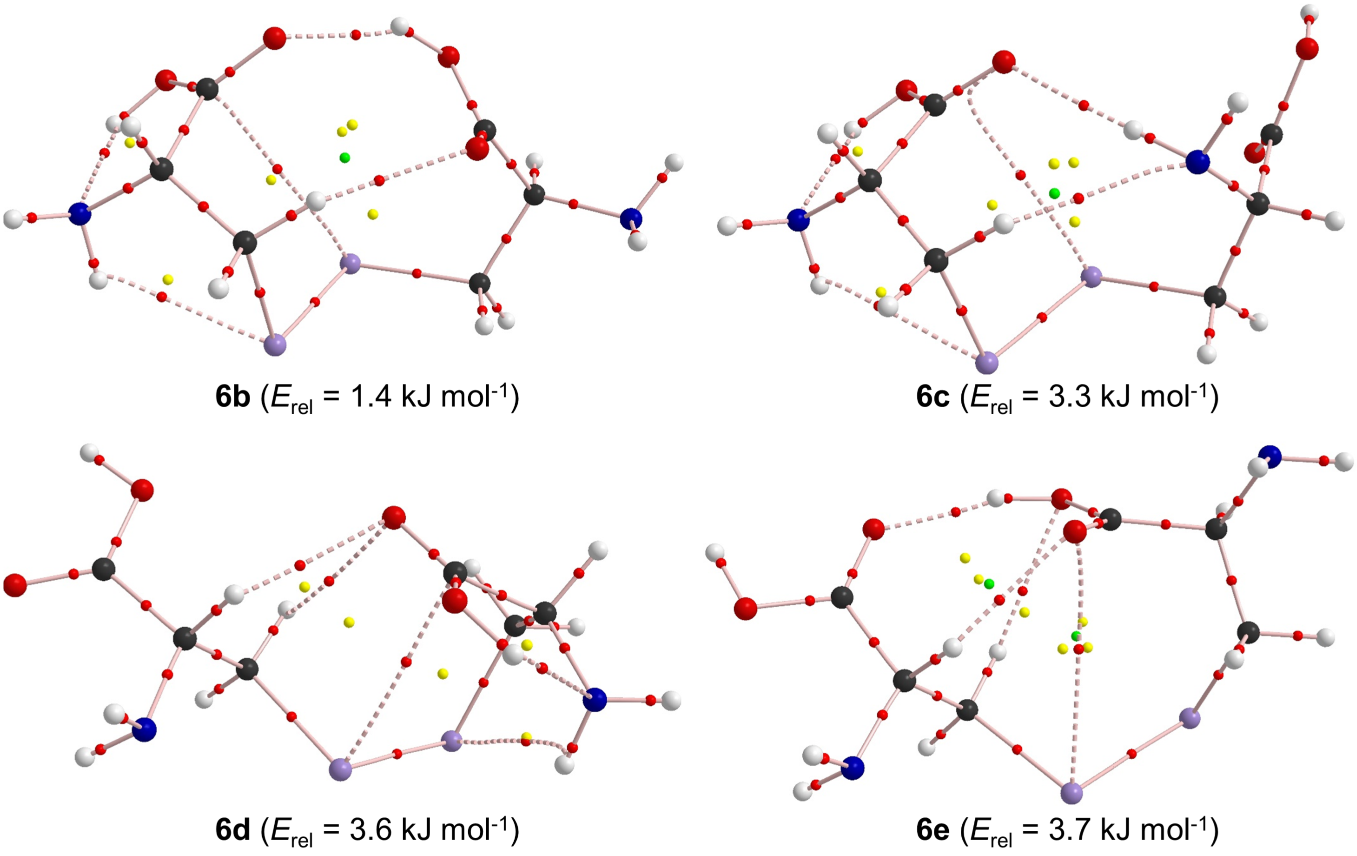

Fig. S5 Molecular graphs of **6b**–**6e**, drawn on the optimized structures.

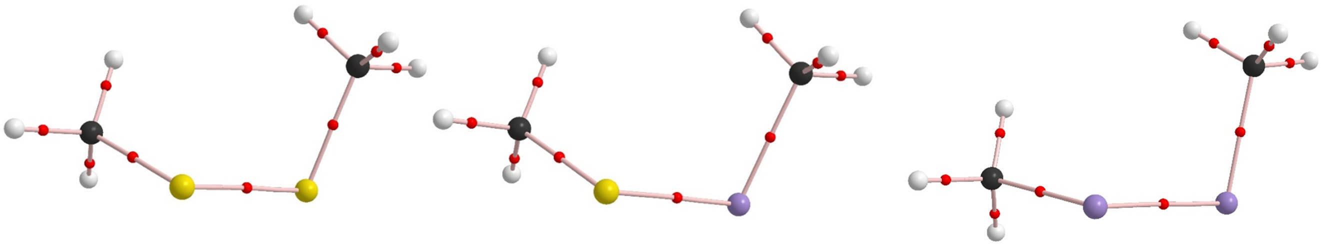

7 **8** **9**

Fig. S6 Molecular graphs of **7**–**9**, drawn on the optimized structures.

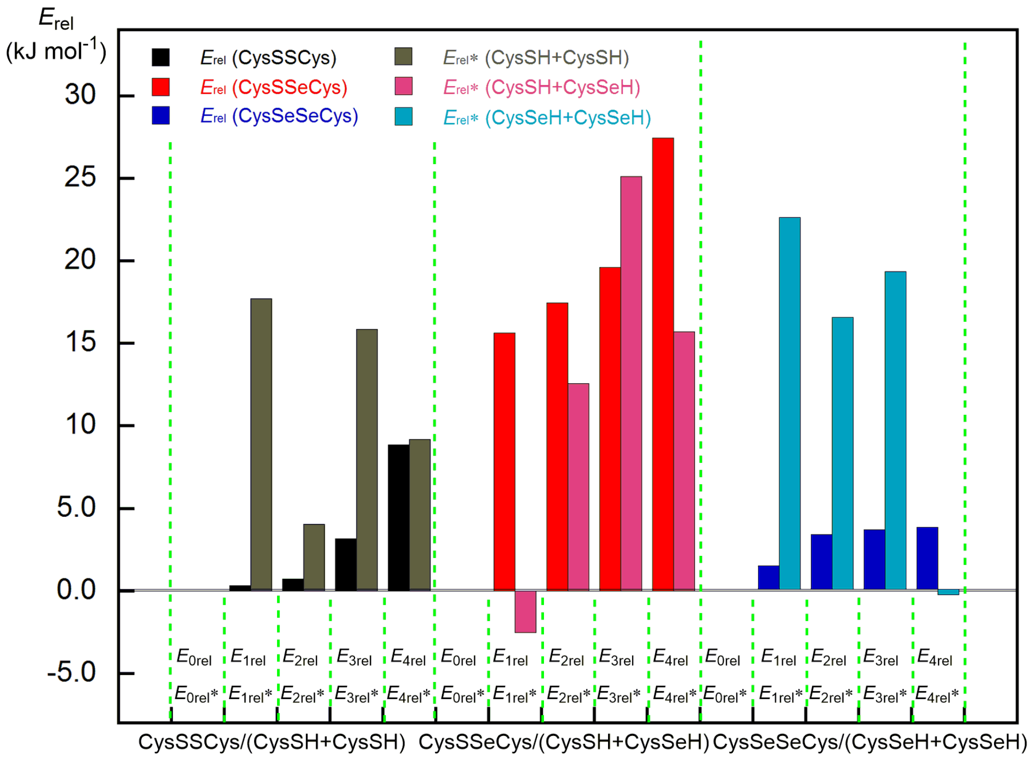

Fig. S7 Plots of E_{rel} of REE'R and (2R–H + MeEE'Me) for **4a**–**4e** (CysSSCys), **5a**–**5e** (CysSSeCys) and **6a**–**6e** (CysSeSeCys), evaluated with M06-2X/BSS-A.

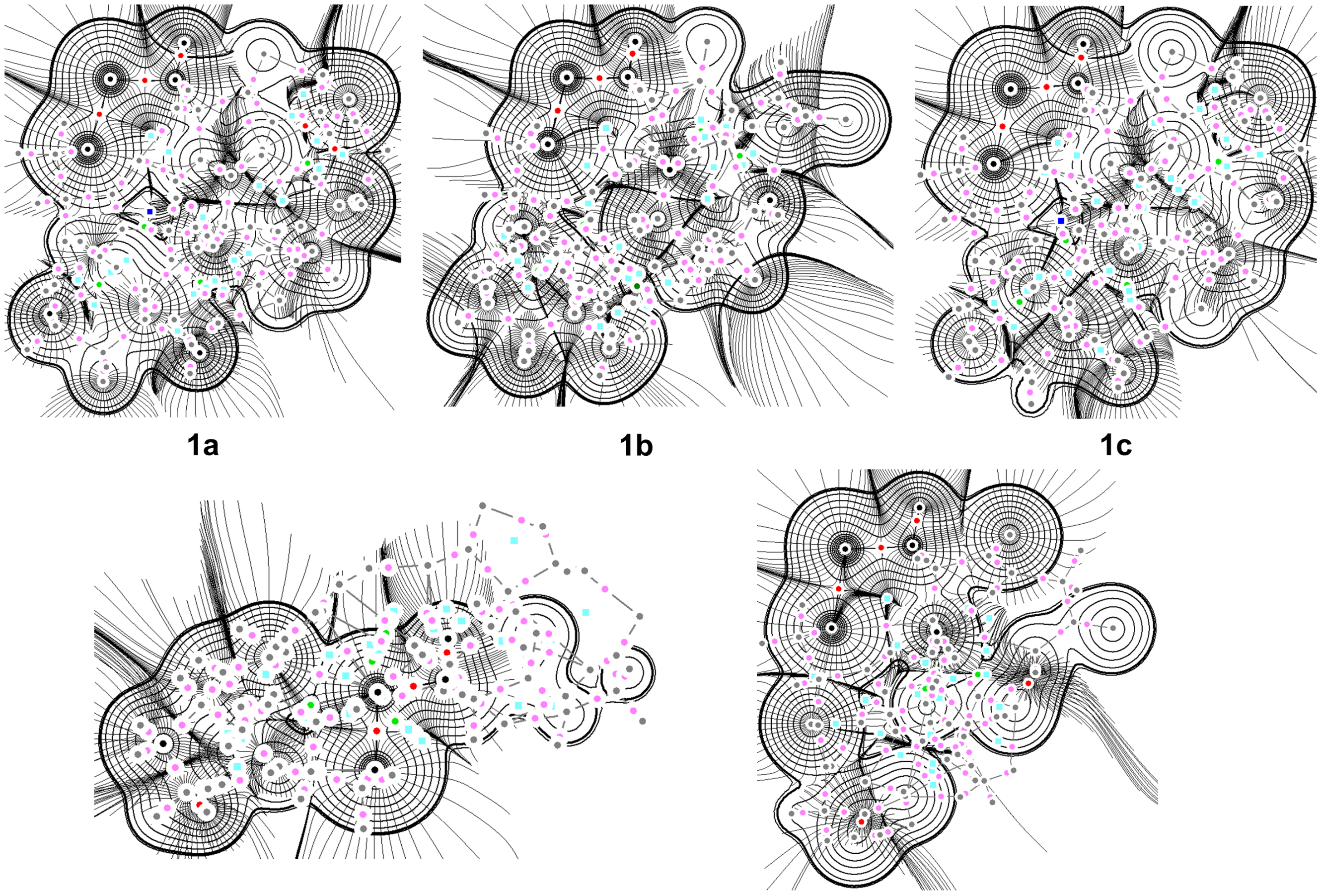

1a **1b** **1c** **1d** **1e**

Fig. S8 Trajectory plots of $\rho_b(\boldsymbol{r}_c)$ drawn on the S–S–C planes of **1a**–**1e**, similarly to the case of Fig. 6 in the text. Color and marks are same as those in Fig. 6.

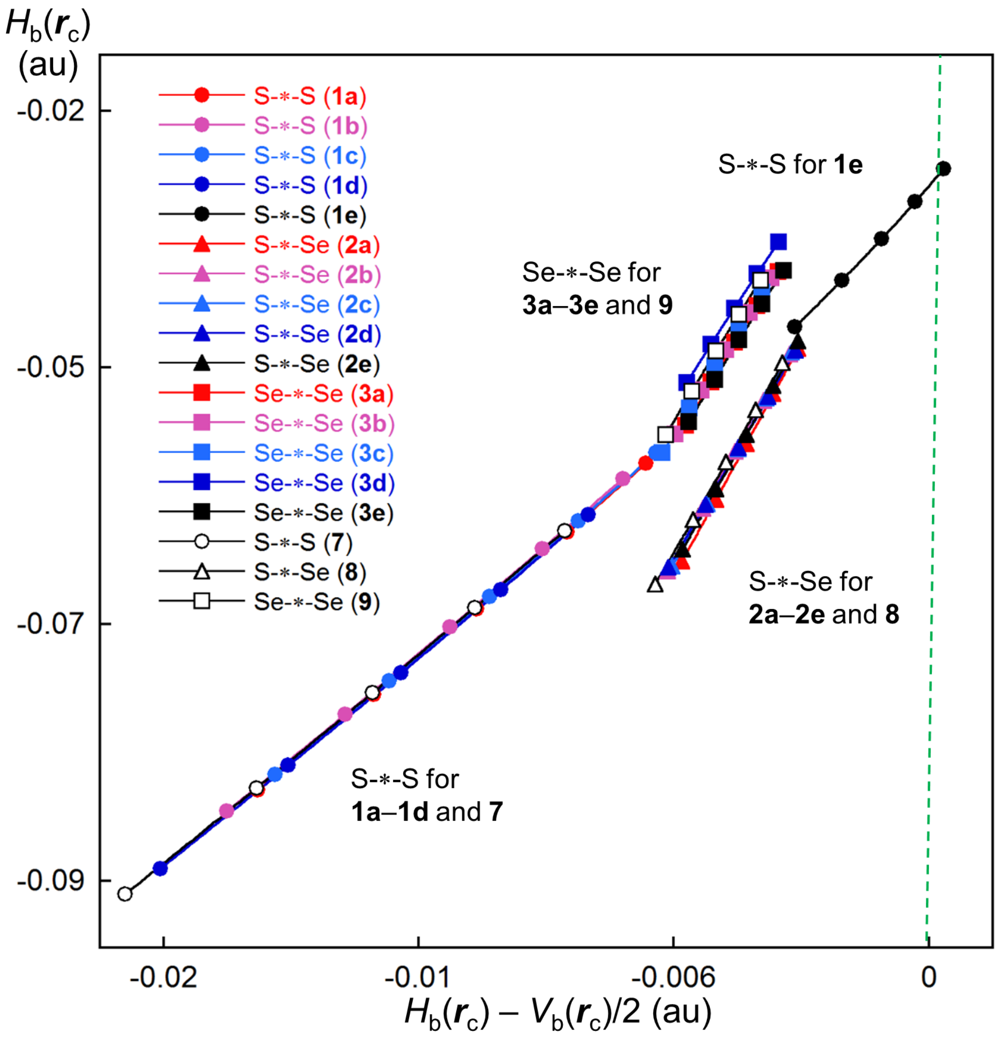

Fig. S9 Plots of $H_b(\boldsymbol{r}_c)$ versus $H_b(\boldsymbol{r}_c) - V_b(\boldsymbol{r}_c)/2$ for **1a**–**3e** and **7**–**9**.

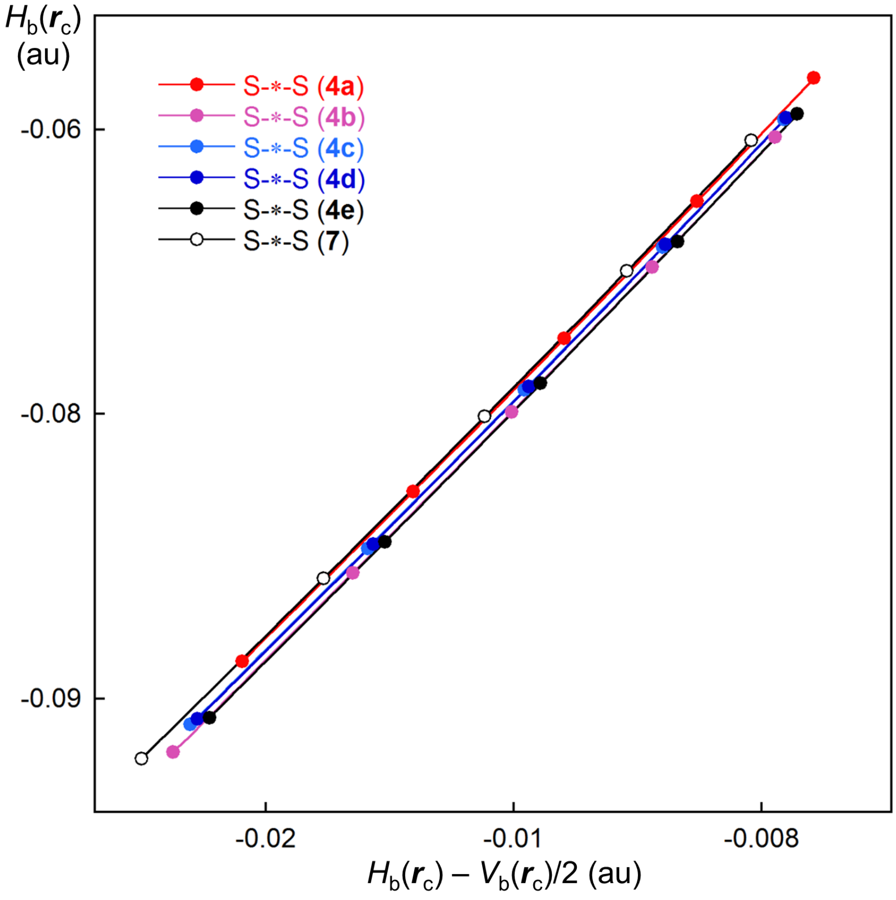

Fig. S10 Plots of $H_b(\boldsymbol{r}_c)$ versus $H_b(\boldsymbol{r}_c) - V_b(\boldsymbol{r}_c)/2$ for **4a**–**4e** and **7**.

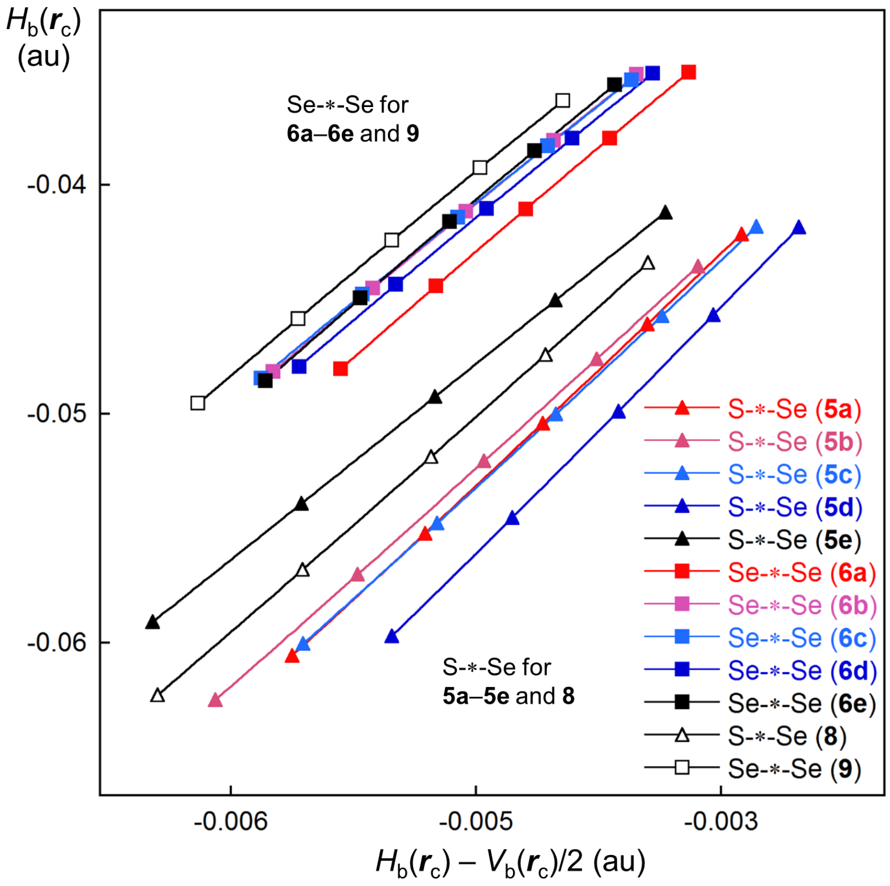

Fig. S11 Plots of $H_b(\boldsymbol{r}_c)$ versus $H_b(\boldsymbol{r}_c) - V_b(\boldsymbol{r}_c)/2$ for **5a**–**6e** and **7**–**8**.

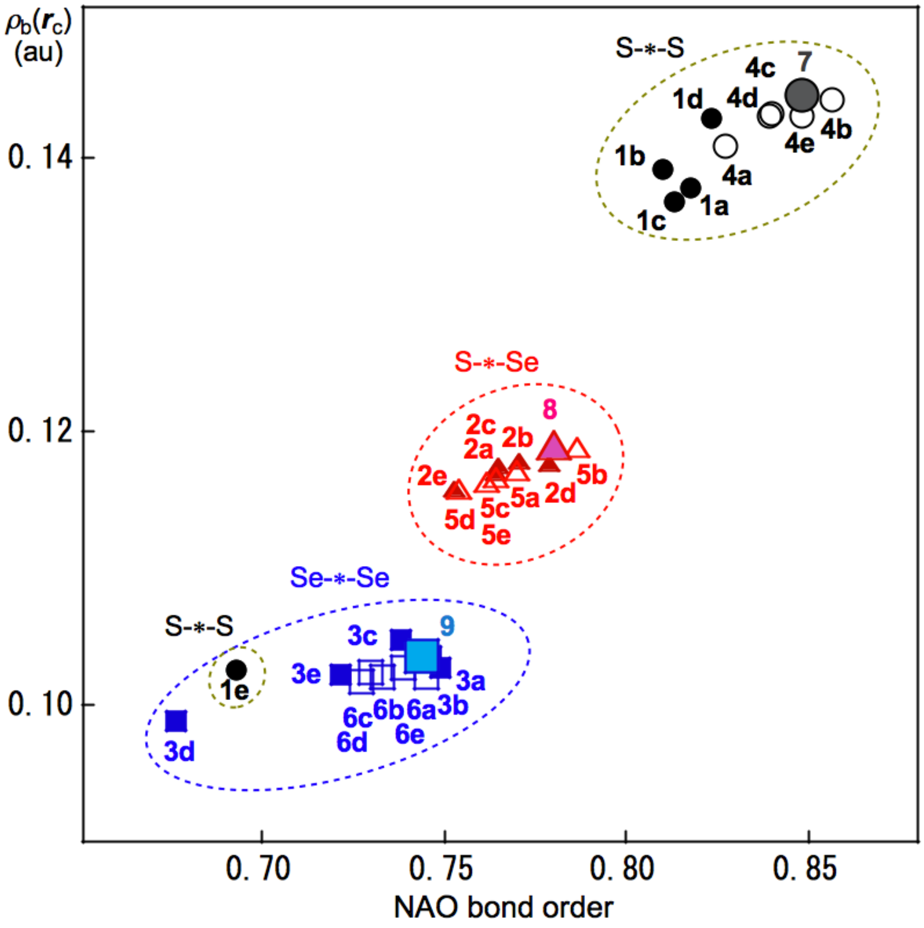

Fig. S12 Plots of $\rho_b(\mathbf{r}_c)$ versus NAO bond orders for **1a**–**6e** and **7**–**8**.

References

[S1] *Atoms in Molecules. A Quantum Theory*: eds. R. F. W. Bader, Oxford University Press, Oxford, UK, 1990; C. F. Matta, R. J. Boyd, *An Introduction to the Quantum Theory of Atoms in Molecules* in *The Quantum Theory of Atoms in Molecules: From Solid State to DNA and Drug Design*: eds. C. F. Matta, R. J. Boyd, WILEY-VCH, Weinheim, Germany, 2007, Chapter 1.

[S2] R. F. W. Bader, T. S. Slee. D. Cremer, E. Kraka, *J. Am. Chem. Soc.*, 1983, **105**, 5061–5068; R. F. W. Bader, *Chem. Rev.*, 1991, **91**, 893–926; R. F. W. Bader, *J. Phys. Chem. A*, 1998, **102**, 7314–7323; F. Biegler-König, R. F. W. Bader, T. H. Tang, *J. Comput. Chem.* 1982, **3**, 317–328; (e) R. F. W. Bader, *Acc. Chem. Res.* 1985, **18**, 9–15; T. H. Tang, R. F. W. Bader, P. MacDougall, *Inorg. Chem.*, 1985, **24**, 2047–2053; F. Biegler-König, J. Schönbohm, D. Bayles, *J. Comput. Chem.*, 2001, **22**, 545–559; F. Biegler-König, J. Schönbohm, *J. Comput. Chem.*, 2002, **23**, 1489–1494.

[S3] W. Nakanishi, T. Nakamoto, S. Hayashi, T. Sasamori, N. Tokitoh, *Chem. Eur. J.*, 2007, **13**, 255–268.

[S4] W. Nakanishi, S. Hayashi, K. Narahara, *J. Phys. Chem. A*, 2009, **113**, 10050–10057; W. Nakanishi, S. Hayashi, K. Narahara, *J. Phys. Chem. A*, 2008, **112**, 13593–13599.

[S5] W. Nakanishi, S. Hayashi, *Curr. Org. Chem.*, 2010, **14**, 181–197.

[S6] W. Nakanishi, S. Hayashi, *J. Phys. Chem. A*, 2010, **114**, 7423–7430; W. Nakanishi, S. Hayashi, K. Matsuiwa, M. Kitamoto, *Bull. Chem. Soc. Jpn*, 2012, **85**, 1293–1305.

Optimized structures given by Cartesian coordinates

Compound **1a**

Level M06-2X/BSS-A

Energy HF = -2809.0236828 au

Standard orientation

16	0	1.740613	2.724376	-1.490107
16	0	0.224532	4.137765	-1.429609
6	0	-1.351875	3.232693	-1.255750
6	0	2.733283	3.119727	0.009364
1	0	3.624797	3.641037	-0.338091
1	0	2.134628	3.791352	0.622367
1	0	-1.610125	2.750811	-2.198610
1	0	-2.074117	4.034460	-1.080596
6	0	3.163985	1.910492	0.841740
6	0	-1.410132	2.231514	-0.111255
1	0	-0.835012	2.609964	0.734632
7	0	-0.811531	0.958108	-0.475946
1	0	0.142910	0.784828	-0.185459
6	0	-2.836302	1.997077	0.397331
8	0	-3.785183	2.704485	0.083251
7	0	-2.956880	0.971667	1.269268
1	0	-2.208889	0.290918	1.323824
6	0	-4.244724	0.588951	1.814618
1	0	-4.790163	1.490575	2.103864
1	0	-4.090536	-0.047499	2.682591
6	0	-5.062882	-0.198843	0.796853
8	0	-5.436776	-1.329495	0.979972
8	0	-5.299946	0.407309	-0.364773
1	0	-4.920467	1.310433	-0.382831
6	0	-1.416638	0.108421	-1.316108
8	0	-2.583863	0.301541	-1.691637
6	0	-0.598844	-1.075125	-1.763683
1	0	0.078803	-0.731544	-2.552560
1	0	0.039025	-1.373756	-0.930669
6	0	-1.407270	-2.270057	-2.268797
1	0	-0.684736	-3.036731	-2.552628
1	0	-1.967936	-2.003125	-3.169816
6	0	-2.367085	-2.870310	-1.228694
1	0	-2.592233	-3.909553	-1.469462
7	0	-3.668282	-2.118015	-1.216862
1	0	-3.469747	-1.100433	-1.394986
1	0	-4.301119	-2.441296	-1.946907
6	0	-1.731084	-2.819325	0.175283
8	0	-2.250255	-1.996679	0.978892
1	0	-4.143619	-2.191989	-0.306006
8	0	-0.755614	-3.556840	0.346855
1	0	3.980103	2.227557	1.500832
7	0	3.698182	0.839642	0.010058
1	0	3.239075	0.587397	-0.876872
6	0	4.591468	-0.054242	0.496194

8	0	4.984268	-0.038704	1.660031
6	0	5.052592	-1.126640	-0.475505
1	0	4.940501	-0.789813	-1.505263
1	0	6.106605	-1.309920	-0.264745
6	0	4.261734	-2.427259	-0.254236
1	0	4.608964	-3.183525	-0.965601
1	0	4.468836	-2.791451	0.758745
6	0	2.760277	-2.201248	-0.416520
1	0	2.408566	-1.446457	0.279951
7	0	1.983255	-3.451536	-0.147507
1	0	1.042539	-3.263720	0.257521
1	0	2.471019	-4.127037	0.437388
6	0	2.374365	-1.791540	-1.873620
8	0	2.600699	-0.608966	-2.177543
8	0	1.866883	-2.709757	-2.548892
1	0	1.808546	-3.819392	-1.110932
6	0	2.024057	1.427200	1.752949
8	0	0.892590	1.881504	1.670063
7	0	2.338920	0.475838	2.657836
1	0	3.284332	0.104279	2.661713
6	0	1.260104	-0.162729	3.372309
1	0	0.679319	0.564174	3.943098
1	0	1.664355	-0.896298	4.071371
6	0	0.313952	-0.873582	2.408658
8	0	0.615565	-1.084415	1.251931
8	0	-0.825374	-1.226301	2.945144
1	0	-1.400040	-1.668924	2.218540

Compound **1b**
Level M06-2X/BSS-A
Energy HF = -2809.0204116 au
Standard orientation

16	0	0.749832	3.006024	-0.874248
16	0	-0.886999	4.225978	-0.535408
6	0	-2.341963	3.138915	-0.522123
6	0	1.655842	3.250187	0.702181
1	0	2.185344	4.198916	0.622818
1	0	0.917471	3.324454	1.499729
1	0	-2.501986	2.732464	-1.519686
1	0	-3.155640	3.837540	-0.310127
6	0	2.662630	2.146447	1.041459
6	0	-2.379409	2.016157	0.515421
1	0	-2.029811	2.398854	1.479070
7	0	-1.516712	0.900746	0.167582
1	0	-0.622037	0.839159	0.646088
6	0	-3.842775	1.581992	0.732396
8	0	-4.739485	2.399707	0.696043
7	0	-4.031932	0.264792	0.997451
1	0	-3.255245	-0.380381	0.893711

6	0	-5.357400	-0.294643	1.079251
1	0	-6.064452	0.522834	1.236392
1	0	-5.443050	-0.985926	1.917662
6	0	-5.813564	-1.053088	-0.169321
8	0	-6.694543	-1.863188	-0.104957
8	0	-5.210285	-0.777111	-1.327246
1	0	-4.449223	-0.168914	-1.251689
6	0	-1.687411	0.211144	-0.979764
8	0	-2.689646	0.324205	-1.680336
6	0	-0.544771	-0.698220	-1.372924
1	0	-0.049013	-0.190688	-2.206782
1	0	0.187412	-0.764767	-0.559412
6	0	-1.011114	-2.079151	-1.833966
1	0	-0.168661	-2.611898	-2.279932
1	0	-1.766688	-1.955218	-2.613912
6	0	-1.608324	-2.951992	-0.719722
1	0	-1.709818	-3.972393	-1.121599
7	0	-2.869743	-2.404072	-0.233821
1	0	-3.286231	-3.028314	0.450798
1	0	-3.522489	-2.322942	-1.009944
6	0	-0.611128	-3.082152	0.413755
8	0	-1.123967	-2.968492	1.611118
1	0	-0.409226	-2.963732	2.293185
8	0	0.578788	-3.285668	0.207210
1	0	3.458796	2.598273	1.639417
7	0	3.306900	1.566139	-0.126358
1	0	2.794024	1.451047	-1.000280
6	0	4.509120	0.947297	-0.013835
8	0	5.118435	0.880748	1.047189
6	0	5.043689	0.308253	-1.279366
1	0	4.699281	0.847571	-2.161816
1	0	6.130352	0.379598	-1.225325
6	0	4.657049	-1.175102	-1.357191
1	0	5.150876	-1.624274	-2.225103
1	0	5.031804	-1.684432	-0.463693
6	0	3.149829	-1.474748	-1.451347
1	0	2.650415	-1.087321	-0.555997
7	0	2.940893	-2.911649	-1.630928
1	0	2.101990	-3.232362	-1.154003
1	0	3.727913	-3.453051	-1.293825
6	0	2.534759	-0.736640	-2.646053
8	0	2.356736	0.457527	-2.656179
8	0	2.221354	-1.508708	-3.672222
1	0	2.441835	-2.422810	-3.384012
6	0	2.036352	1.040298	1.902486
8	0	0.860470	0.703159	1.829847
7	0	2.891677	0.465721	2.773929
1	0	3.884497	0.610844	2.611328
6	0	2.430044	-0.609229	3.618008
1	0	1.668413	-0.260396	4.316212

1	0	3.274251	-0.997687	4.190359
6	0	1.812470	-1.755636	2.842920
8	0	0.896791	-2.415206	3.283559
8	0	2.383802	-1.978053	1.672819
1	0	1.844842	-2.649722	1.191541

Compound **1c**
Level M06-2X/BSS-A
Energy HF = -2809.018305 au
Standard orientation

16	0	1.625831	2.593329	-1.735974
16	0	0.112027	4.014651	-1.662259
6	0	-1.464109	3.139087	-1.372548
6	0	2.710593	3.099141	-0.339770
1	0	3.590449	3.564261	-0.784050
1	0	2.160618	3.842039	0.235565
1	0	-1.770303	2.614438	-2.277397
1	0	-2.167042	3.961534	-1.214568
6	0	3.170449	1.973556	0.584389
6	0	-1.498311	2.195901	-0.178998
1	0	-0.936597	2.623004	0.652521
7	0	-0.865130	0.922259	-0.495349
1	0	0.118996	0.836223	-0.278281
6	0	-2.927435	1.935679	0.315467
8	0	-3.910647	2.506675	-0.146126
7	0	-3.012104	1.034280	1.307917
1	0	-2.185037	0.553452	1.648026
6	0	-4.286015	0.608797	1.852164
1	0	-4.931369	1.478270	2.003834
1	0	-4.110058	0.099192	2.796126
6	0	-4.954940	-0.370432	0.895458
8	0	-5.176587	-1.525018	1.162489
8	0	-5.237425	0.099883	-0.319416
1	0	-4.938636	1.029809	-0.425157
6	0	-1.440384	0.039061	-1.323492
8	0	-2.613505	0.177816	-1.696463
6	0	-0.581556	-1.114404	-1.779170
1	0	0.086577	-0.749966	-2.566956
1	0	0.078791	-1.395671	-0.956373
6	0	-1.356821	-2.333765	-2.288327
1	0	-0.612590	-3.073146	-2.588843
1	0	-1.940738	-2.068041	-3.174408
6	0	-2.279519	-2.982280	-1.237698
1	0	-2.472136	-4.026859	-1.478526
7	0	-3.597851	-2.264808	-1.178242
1	0	-4.251499	-2.616485	-1.876066
1	0	-3.436526	-1.244081	-1.379216
6	0	-1.569943	-2.871466	0.117171
8	0	-1.927845	-1.916968	0.847093

1	0	-4.043558	-2.327898	-0.249916
8	0	-0.621486	-3.654458	0.291615
1	0	3.981881	2.366715	1.208023
7	0	3.722532	0.844862	-0.146873
1	0	3.235669	0.475055	-0.977752
6	0	4.625504	0.017825	0.426782
8	0	5.054244	0.178131	1.566914
6	0	5.040588	-1.179199	-0.406049
1	0	4.907128	-0.982339	-1.469072
1	0	6.095731	-1.359468	-0.198661
6	0	4.234364	-2.418149	0.012478
1	0	4.574428	-3.273663	-0.581345
1	0	4.445745	-2.634069	1.066090
6	0	2.722985	-2.258005	-0.180761
1	0	2.311984	-1.483095	0.470163
7	0	2.043071	-3.547875	0.148206
1	0	1.043656	-3.430830	0.444408
1	0	2.542347	-4.109764	0.834560
6	0	2.361847	-1.969409	-1.677925
8	0	2.547880	-0.802140	-2.063128
8	0	1.924959	-2.953884	-2.300703
1	0	1.977948	-4.020735	-0.777769
6	0	2.055033	1.545089	1.547517
8	0	0.926319	2.009146	1.483003
7	0	2.384352	0.629607	2.482614
1	0	3.353412	0.336622	2.563219
6	0	1.386917	0.266497	3.467190
1	0	1.103650	1.123512	4.080560
1	0	1.799470	-0.512672	4.110336
6	0	0.101459	-0.261711	2.843315
8	0	-0.989592	0.058491	3.250071
8	0	0.294972	-1.122750	1.858580
1	0	-0.616726	-1.461588	1.550153

Compound **1d**
Level M06-2X/BSS-A
Energy HF = -2809.0125414 au
Standard orientation

16	0	-1.635509	-2.780722	-0.052882
16	0	-0.954823	-1.382144	-1.397594
6	0	0.786888	-1.845415	-1.618527
6	0	-1.642120	-1.942478	1.567207
1	0	-1.947623	-2.750809	2.236860
1	0	-0.634663	-1.623537	1.832772
1	0	1.168029	-1.137091	-2.361276
1	0	0.849231	-2.850860	-2.036319
6	0	-2.604802	-0.755544	1.730660
6	0	1.636092	-1.756120	-0.360431
1	0	1.317063	-2.509349	0.367616

7	0	1.536858	-0.435838	0.231451
1	0	1.023111	0.300374	-0.246662
6	0	3.110570	-1.963518	-0.746688
8	0	3.627101	-1.297298	-1.622262
7	0	3.829688	-2.871582	-0.028811
1	0	3.436524	-3.169449	0.853203
6	0	5.271041	-2.833505	-0.185212
1	0	5.550734	-3.072817	-1.209688
1	0	5.722523	-3.562557	0.489786
6	0	5.859692	-1.453253	0.120131
8	0	6.739719	-0.958015	-0.533612
8	0	5.381373	-0.801392	1.180394
1	0	4.520897	-1.122722	1.537622
6	0	2.189450	-0.147552	1.361725
8	0	2.828242	-1.023274	1.970637
6	0	2.249356	1.300709	1.772296
1	0	1.477014	1.880902	1.274617
1	0	2.089599	1.350726	2.849977
6	0	3.657272	1.828200	1.438428
1	0	4.379418	1.310681	2.076888
1	0	3.701284	2.896869	1.672428
6	0	4.054324	1.612261	-0.026396
1	0	3.848581	0.600340	-0.365402
7	0	5.522636	1.860594	-0.224077
1	0	6.024528	1.034202	-0.573229
1	0	5.983213	2.158401	0.634635
6	0	3.341568	2.602832	-0.989602
8	0	4.092114	3.417846	-1.548079
1	0	5.483999	2.656787	-0.925911
8	0	2.106425	2.448519	-1.063259
1	0	-2.870077	-0.694845	2.793938
7	0	-3.841536	-0.953157	1.003210
1	0	-3.840165	-1.517182	0.154292
6	0	-5.036306	-0.475227	1.451844
8	0	-5.114253	0.288485	2.399742
6	0	-6.258650	-0.965216	0.693180
1	0	-6.122632	-2.018184	0.440348
1	0	-7.094105	-0.879322	1.388251
6	0	-6.594166	-0.166134	-0.575881
1	0	-7.507289	-0.593552	-0.999856
1	0	-6.812192	0.872754	-0.322194
6	0	-5.503946	-0.175752	-1.655432
1	0	-5.967479	0.172037	-2.591979
7	0	-4.894298	-1.500121	-1.788699
1	0	-4.123183	-1.472741	-2.448248
1	0	-5.576215	-2.168124	-2.132440
6	0	-4.435592	0.878654	-1.375860
8	0	-4.682029	1.936433	-0.845199
8	0	-3.248369	0.519421	-1.822491
1	0	-2.528426	1.159697	-1.569077

6	0	-1.885762	0.580567	1.465384
8	0	-0.678347	0.697039	1.627772
7	0	-2.659322	1.618892	1.113487
1	0	-3.664099	1.528745	1.075002
6	0	-2.053335	2.895613	0.827369
1	0	-1.587647	3.332103	1.712435
1	0	-2.830970	3.564297	0.454062
6	0	-0.991274	2.744821	-0.254204
8	0	-1.106656	1.966238	-1.182655
8	0	0.032385	3.536259	-0.080927
1	0	0.794708	3.272828	-0.681198

Compound **1e**
Level M06-2X/BSS-A
Energy HF = -2808.9865998 au
Standard orientation

16	0	0.508245	-1.309048	-2.597885
16	0	-1.074871	-2.811381	-3.125491
6	0	-2.237547	-2.732581	-1.649099
6	0	2.063352	-2.377257	-2.503896
1	0	1.753408	-3.418655	-2.501204
1	0	2.647673	-2.156604	-3.390634
1	0	-1.721534	-3.056795	-0.749005
1	0	-3.006427	-3.464516	-1.883363
6	0	2.873253	-2.072949	-1.251222
6	0	-2.860094	-1.359766	-1.436867
1	0	-3.218573	-0.931104	-2.373760
7	0	-1.831101	-0.483089	-0.880243
1	0	-1.185100	-0.928196	-0.222782
6	0	-3.992067	-1.425636	-0.407791
8	0	-4.207766	-2.445063	0.268369
7	0	-4.653366	-0.251854	-0.216597
1	0	-4.325410	0.555890	-0.733403
6	0	-5.284802	-0.036123	1.071493
1	0	-5.933532	-0.871376	1.320852
1	0	-5.887306	0.869789	1.041050
6	0	-4.255251	0.080323	2.188066
8	0	-4.523899	-0.085647	3.371544
8	0	-3.033286	0.414141	1.718645
1	0	-2.229731	0.273314	2.308769
6	0	-1.778982	0.832169	-0.987381
8	0	-2.703355	1.517131	-1.558931
6	0	-0.572181	1.519430	-0.406564
1	0	0.045392	0.805548	0.129947
1	0	0.056631	1.852538	-1.242444
6	0	-0.969591	2.695112	0.507663
1	0	-0.223424	2.800476	1.292473
1	0	-1.931496	2.487919	0.977025
6	0	-0.991737	4.039741	-0.225919

1	0	-1.320505	4.831740	0.443886
7	0	-1.882122	4.009988	-1.445022
1	0	-2.304832	3.027930	-1.582488
1	0	-2.627026	4.698106	-1.401719
6	0	0.423610	4.360070	-0.737515
8	0	0.572205	4.502930	-1.973511
1	0	-1.240446	4.229363	-2.234807
8	0	1.317330	4.386803	0.191008
1	0	3.805797	-2.641555	-1.292750
7	0	2.171457	-2.498551	-0.042396
1	0	1.167130	-2.339182	0.020917
6	0	2.845982	-2.812508	1.086567
8	0	4.098610	-2.728822	1.164931
6	0	2.013755	-3.159846	2.298870
1	0	1.000497	-3.429712	2.014145
1	0	2.482325	-4.005473	2.798401
6	0	2.001661	-1.950516	3.256545
1	0	1.349404	-2.156154	4.107298
1	0	3.020111	-1.801273	3.624369
6	0	1.549371	-0.680896	2.527710
1	0	2.155733	-0.508636	1.639996
7	0	1.704226	0.562136	3.358876
1	0	2.043578	1.291198	2.688525
1	0	2.354766	0.451980	4.131546
6	0	0.079177	-0.721620	2.102579
8	0	-0.219688	-1.500003	1.146496
8	0	-0.699854	0.063914	2.747732
1	0	0.749978	0.801547	3.682339
6	0	3.230184	-0.584236	-1.168953
8	0	2.676796	0.266495	-1.893994
7	0	4.168113	-0.229958	-0.256665
1	0	4.590280	-0.944767	0.323998
6	0	4.544630	1.170419	-0.171880
1	0	4.848110	1.546850	-1.146320
1	0	5.380261	1.271688	0.519130
6	0	3.403247	2.053338	0.308254
8	0	2.566250	1.621441	1.148397
8	0	3.409986	3.252523	-0.176132
1	0	2.487373	3.877539	-0.002022

Compound **2b**
Level M06-2X/BSS-A
Energy HF = -4812.3944946 au
Standard orientation

16	0	-3.699706	-2.214938	0.130917
34	0	-3.740474	-0.425577	-1.148680
6	0	-4.293938	1.008574	0.079345
6	0	-2.058745	-2.231388	0.912154
1	0	-1.842290	-1.253626	1.337734

1	0	-2.153843	-2.940184	1.739621
1	0	-4.999895	0.581197	0.788875
1	0	-4.810694	1.711993	-0.575697
6	0	-0.929636	-2.664508	-0.016304
6	0	-3.169399	1.729714	0.818246
1	0	-3.586365	2.658985	1.222539
7	0	-2.077197	2.134382	-0.046139
1	0	-1.499977	1.399530	-0.441266
6	0	-2.716111	0.930278	2.050586
8	0	-3.469286	0.162125	2.618521
7	0	-1.450327	1.188809	2.451551
1	0	-0.876956	1.818701	1.901737
6	0	-0.854265	0.519463	3.587223
1	0	-1.639267	0.031915	4.161541
1	0	-0.332479	1.247192	4.215247
6	0	0.143136	-0.544790	3.138192
8	0	0.059603	-1.704776	3.476413
8	0	1.117593	-0.151551	2.338811
1	0	1.010692	0.784640	1.979694
6	0	-1.620997	3.424995	-0.027003
8	0	-2.137683	4.282753	0.661522
6	0	-0.517949	3.825457	-0.997787
1	0	-1.057783	4.342684	-1.797089
1	0	0.044104	4.600030	-0.472540
6	0	0.422318	2.784190	-1.621508
1	0	0.551756	3.022169	-2.680609
1	0	0.008152	1.773706	-1.594906
6	0	1.805228	2.757214	-0.972041
1	0	2.175708	3.776143	-0.829461
7	0	2.802071	2.061230	-1.862855
1	0	3.319075	2.715370	-2.448305
1	0	2.336675	1.396064	-2.507626
6	0	1.875061	2.064399	0.410139
8	0	2.943807	1.511056	0.689545
1	0	3.493888	1.541996	-1.285002
8	0	0.843977	2.133159	1.122301
1	0	-1.185570	-3.606179	-0.509807
7	0	0.302489	-2.822481	0.741856
1	0	0.325609	-2.529864	1.717334
6	0	1.456287	-3.001503	0.078012
8	0	1.455378	-3.263109	-1.131573
6	0	2.738970	-2.739337	0.825051
1	0	2.528554	-2.548320	1.879889
1	0	3.394153	-3.609386	0.731799
6	0	3.359020	-1.507145	0.151550
1	0	3.635224	-1.752778	-0.874914
1	0	2.605908	-0.715989	0.109461
6	0	4.584649	-0.958906	0.873778
1	0	5.375594	-1.721486	0.875115
7	0	4.394234	-0.522134	2.255553

1	0	3.515318	-0.010954	2.327406
1	0	4.367559	-1.304171	2.897272
6	0	5.183010	0.220378	0.093314
8	0	5.048856	0.375394	-1.098694
8	0	5.899833	1.046979	0.832797
1	0	5.710688	0.794307	1.761660
6	0	-0.687248	-1.572670	-1.066473
8	0	-0.325294	-0.449664	-0.749764
7	0	-0.868372	-1.923136	-2.359724
1	0	-0.985804	-2.898621	-2.585691
6	0	-0.549912	-0.965107	-3.386775
1	0	-1.207509	-0.095857	-3.316758
1	0	-0.686082	-1.423263	-4.367662
6	0	0.879986	-0.433242	-3.304075
8	0	1.143887	0.670009	-3.731654
8	0	1.828698	-1.183182	-2.789906
1	0	1.545248	-2.003936	-2.321115

Compound **2b**
Level M06-2X/BSS-A
Energy HF = -4812.3941144 au
Standard orientation

16	0	-2.985627	2.752252	-0.988517
34	0	-4.341853	1.663326	0.352597
6	0	-4.470722	-0.118197	-0.474761
6	0	-1.482505	2.926578	0.038053
1	0	-1.554267	3.850167	0.612992
1	0	-1.421471	2.077224	0.720630
1	0	-4.427006	0.043002	-1.550197
1	0	-5.489588	-0.408223	-0.213475
6	0	-0.224046	2.961080	-0.830159
6	0	-3.503156	-1.242205	-0.057434
1	0	-4.091804	-2.152144	0.076610
7	0	-2.846165	-1.007162	1.210065
1	0	-2.372767	-0.119010	1.325531
6	0	-2.474714	-1.516313	-1.166920
8	0	-1.969045	-0.620869	-1.826259
7	0	-2.234207	-2.828532	-1.385837
1	0	-2.488660	-3.472831	-0.646333
6	0	-1.384063	-3.263726	-2.468126
1	0	-1.569961	-2.637090	-3.341208
1	0	-1.642690	-4.290466	-2.734588
6	0	0.117196	-3.226246	-2.218035
8	0	0.902462	-3.317960	-3.126677
8	0	0.438906	-3.097323	-0.945051
1	0	1.430328	-2.976132	-0.805279
6	0	-2.350240	-2.051745	1.924078
8	0	-2.527138	-3.218799	1.605628
6	0	-1.602748	-1.670429	3.188384

1	0	-1.436517	-0.593845	3.222404
1	0	-2.247271	-1.938798	4.029705
6	0	-0.280298	-2.428721	3.309328
1	0	-0.495413	-3.494293	3.435010
1	0	0.250337	-2.078570	4.200894
6	0	0.609446	-2.250422	2.078912
1	0	0.153994	-2.721248	1.208256
7	0	1.944348	-2.881649	2.309277
1	0	2.436319	-2.979175	1.351894
1	0	1.892372	-3.772729	2.798028
6	0	0.895190	-0.763179	1.763932
8	0	1.987656	-0.323614	2.167072
1	0	2.502032	-2.192722	2.833397
8	0	-0.001992	-0.141580	1.149411
1	0	-0.280177	3.754029	-1.581619
7	0	-0.038330	1.661464	-1.444352
1	0	-0.815918	1.002330	-1.444565
6	0	1.151530	1.249766	-1.916376
8	0	2.148115	1.973447	-1.924943
6	0	1.199638	-0.195969	-2.363735
1	0	0.553604	-0.767057	-1.693770
1	0	0.778275	-0.285357	-3.368815
6	0	2.626459	-0.727267	-2.315166
1	0	2.623050	-1.782456	-2.591123
1	0	3.236857	-0.200731	-3.054339
6	0	3.247969	-0.543845	-0.919593
1	0	2.527248	-0.112262	-0.217505
7	0	4.380940	0.430109	-0.972966
1	0	4.032048	1.307873	-1.387954
1	0	5.154051	0.037535	-1.511893
6	0	3.754560	-1.839879	-0.277262
8	0	2.834337	-2.723700	-0.107950
8	0	4.925913	-1.918610	0.044224
1	0	4.738293	0.629511	-0.023393
6	0	0.957251	3.340830	0.073946
8	0	1.521199	4.424804	-0.044558
7	0	1.288319	2.442285	1.014387
1	0	0.839715	1.516679	1.037134
6	0	2.385426	2.700188	1.931527
1	0	2.325128	1.996474	2.755818
1	0	2.329173	3.733516	2.284558
6	0	3.720284	2.471430	1.235604
8	0	4.516975	1.636002	1.584683
8	0	3.959548	3.208060	0.151066
1	0	3.209162	3.812852	-0.043175

Compound **2c**
Level M06-2X/BSS-A
Energy HF = -4812.3876424 au

Standard orientation

16	0	-3.128068	-2.710605	-0.901983
34	0	-3.392965	-0.712375	-1.778937
6	0	-4.447341	0.226848	-0.407623
6	0	-1.689105	-2.587271	0.196667
1	0	-1.785566	-1.714470	0.839743
1	0	-1.756342	-3.474459	0.832988
1	0	-5.107549	-0.505351	0.054226
1	0	-5.038976	0.939186	-0.985117
6	0	-0.337404	-2.570381	-0.508636
6	0	-3.655394	0.974017	0.663190
1	0	-4.347528	1.670087	1.150230
7	0	-2.592397	1.797121	0.120096
1	0	-1.820530	1.317435	-0.332956
6	0	-3.197521	0.040195	1.793815
8	0	-3.835641	-0.953246	2.088490
7	0	-2.086609	0.453285	2.444171
1	0	-1.583159	1.264978	2.104674
6	0	-1.535658	-0.275839	3.564975
1	0	-2.284684	-0.971427	3.937207
1	0	-1.252777	0.426193	4.354411
6	0	-0.305020	-1.078620	3.153652
8	0	-0.233382	-2.279151	3.296995
8	0	0.694747	-0.414695	2.606164
1	0	0.494506	0.545681	2.401802
6	0	-2.474353	3.104246	0.501453
8	0	-3.256932	3.630047	1.269342
6	0	-1.414192	3.963268	-0.171083
1	0	-1.971373	4.484531	-0.956151
1	0	-1.158176	4.724462	0.568046
6	0	-0.153560	3.345076	-0.786416
1	0	0.073845	3.884214	-1.710467
1	0	-0.295912	2.302868	-1.082546
6	0	1.075653	3.438652	0.121553
1	0	1.081727	4.394159	0.652078
7	0	2.344632	3.366872	-0.678256
1	0	2.748272	4.277518	-0.882633
1	0	2.192769	2.854988	-1.572450
6	0	1.198196	2.316401	1.185049
8	0	2.322216	1.810785	1.307926
1	0	2.999465	2.788504	-0.108821
8	0	0.152621	2.046952	1.818810
1	0	-0.275259	-3.393757	-1.225222
7	0	0.706744	-2.710055	0.484196
1	0	0.476879	-2.564376	1.462178
6	0	2.007804	-2.750916	0.119009
8	0	2.362106	-2.883500	-1.044815
6	0	2.986877	-2.520822	1.251101
1	0	2.487059	-2.628318	2.215159
1	0	3.778695	-3.271430	1.181119

6	0	3.537150	-1.087829	1.128571
1	0	2.723003	-0.400982	0.886989
1	0	3.930112	-0.750406	2.093411
6	0	4.655279	-0.976817	0.079442
1	0	4.306205	-1.423487	-0.857015
7	0	5.892690	-1.583336	0.585405
1	0	5.701339	-2.254711	1.319547
1	0	6.408019	-2.063797	-0.143106
6	0	4.938331	0.503622	-0.173623
8	0	4.258543	1.205797	-0.903286
8	0	5.970906	0.992749	0.467350
1	0	6.396340	0.214129	0.896059
6	0	-0.155765	-1.234753	-1.244107
8	0	-0.225930	-0.157778	-0.670326
7	0	0.054984	-1.331160	-2.578849
1	0	0.445671	-2.201651	-2.910779
6	0	0.315449	-0.120888	-3.316665
1	0	-0.553963	0.538180	-3.283420
1	0	0.519551	-0.368255	-4.359881
6	0	1.495480	0.662831	-2.767633
8	0	1.559368	1.876864	-2.863622
8	0	2.412046	-0.085802	-2.219641
1	0	3.123157	0.462821	-1.784333

Compound **2d**
Level M06-2X/BSS-A
Energy HF = -4812.3856922 au
Standard orientation

16	0	-3.245012	-2.562652	-0.708363
34	0	-3.700142	-0.546310	-1.449314
6	0	-4.475402	0.423916	0.088661
6	0	-1.703571	-2.497981	0.252232
1	0	-1.700729	-1.600596	0.864209
1	0	-1.801327	-3.347661	0.933557
1	0	-5.053171	-0.291391	0.670822
1	0	-5.154516	1.126245	-0.397319
6	0	-0.387714	-2.631563	-0.500210
6	0	-3.497123	1.191887	0.989622
1	0	-3.990116	2.085897	1.383937
7	0	-2.323425	1.636446	0.253684
1	0	-1.863094	0.930665	-0.318963
6	0	-3.032800	0.329278	2.177112
8	0	-3.271662	-0.859698	2.246853
7	0	-2.267982	0.982841	3.096572
1	0	-2.027550	1.946508	2.888316
6	0	-1.302413	0.198445	3.839940
1	0	-1.779912	-0.674866	4.278330
1	0	-0.875213	0.810860	4.636023
6	0	-0.164452	-0.284760	2.943232

8	0	0.339097	-1.381830	3.045775
8	0	0.198568	0.622774	2.057043
1	0	0.965485	0.296452	1.508209
6	0	-1.570122	2.697780	0.628221
8	0	-1.881473	3.465769	1.527531
6	0	-0.300550	2.859142	-0.189799
1	0	0.240924	1.906618	-0.151752
1	0	-0.605390	2.999462	-1.232120
6	0	0.566799	4.003384	0.315100
1	0	0.678528	3.921829	1.397378
1	0	0.090613	4.967678	0.116198
6	0	1.973394	3.958587	-0.260015
1	0	2.536838	4.848083	0.031945
7	0	1.985285	3.917815	-1.765018
1	0	1.766785	4.813082	-2.197662
1	0	1.352458	3.184997	-2.146100
6	0	2.800698	2.717294	0.178592
8	0	3.419037	2.133524	-0.728780
1	0	2.924186	3.578923	-2.022183
8	0	2.756208	2.469160	1.401225
1	0	-0.414116	-3.445499	-1.227825
7	0	0.651859	-2.920262	0.483444
1	0	0.579220	-2.432980	1.374771
6	0	1.886267	-3.340769	0.118008
8	0	2.151344	-3.711036	-1.020433
6	0	2.925501	-3.315667	1.223133
1	0	2.555873	-2.722242	2.063642
1	0	3.048508	-4.341674	1.581242
6	0	4.276550	-2.795161	0.722681
1	0	4.952738	-2.695635	1.577477
1	0	4.721090	-3.510635	0.029596
6	0	4.214790	-1.449085	-0.015350
1	0	3.683657	-1.612571	-0.957210
7	0	5.562083	-0.993636	-0.315157
1	0	6.052947	-0.813794	0.556172
1	0	5.514885	-0.097665	-0.792212
6	0	3.368560	-0.438619	0.747656
8	0	2.159635	-0.359671	0.541741
8	0	4.011397	0.311322	1.593573
1	0	3.488642	1.197917	1.694991
6	0	-0.002175	-1.321330	-1.198336
8	0	-0.465134	-0.236177	-0.874694
7	0	0.933852	-1.413401	-2.166176
1	0	1.436034	-2.291303	-2.258781
6	0	1.520511	-0.180476	-2.632256
1	0	2.249975	-0.403954	-3.414681
1	0	2.019716	0.365296	-1.823810
6	0	0.488034	0.750107	-3.230335
8	0	0.562462	1.962539	-3.183408
8	0	-0.485831	0.129768	-3.875012

1	0	-1.118522	0.792302	-4.185794

Compound **2e**

Level M06-2X/BSS-A

Energy HF = -4812.3854497 au

Standard orientation

16	0	1.763735	2.937089	-2.605816
34	0	1.270526	3.360012	-0.495716
6	0	-0.689923	3.128026	-0.592235
6	0	1.314200	1.197417	-2.839981
1	0	0.304839	1.006243	-2.470128
1	0	1.273966	1.080278	-3.926823
1	0	-0.876460	2.301724	-1.281069
1	0	-1.146505	4.029868	-0.990633
6	0	2.262573	0.135718	-2.276392
6	0	-1.284414	2.797968	0.781118
1	0	-1.273231	3.674782	1.433410
7	0	-0.534137	1.738826	1.412660
1	0	0.016499	1.113005	0.834026
6	0	-2.768657	2.465096	0.533802
8	0	-3.505852	3.319031	0.084666
7	0	-3.132718	1.187894	0.791339
1	0	-2.472680	0.543453	1.213333
6	0	-4.457967	0.695587	0.477213
1	0	-5.034798	1.529618	0.075459
1	0	-4.943081	0.285792	1.362704
6	0	-4.329522	-0.410789	-0.556182
8	0	-4.496712	-1.581002	-0.294412
8	0	-3.950117	0.041322	-1.738405
1	0	-3.703898	-0.716599	-2.324554
6	0	-0.592523	1.511478	2.749012
8	0	-1.312527	2.135453	3.503602
6	0	0.355203	0.430593	3.235446
1	0	0.780613	-0.082662	2.369994
1	0	1.176343	0.937400	3.748382
6	0	-0.345390	-0.564353	4.175508
1	0	-1.385128	-0.260379	4.307193
1	0	0.105824	-0.557506	5.171049
6	0	-0.340685	-1.979467	3.625404
1	0	-1.010965	-2.620020	4.203943
7	0	1.020949	-2.623818	3.686563
1	0	1.165265	-3.171866	4.531605
1	0	1.805444	-1.941715	3.604427
6	0	-0.777218	-2.111415	2.135992
8	0	-0.224014	-3.046762	1.517791
1	0	1.041842	-3.231904	2.839927
8	0	-1.641971	-1.316899	1.724466
1	0	3.278276	0.304264	-2.649327
7	0	1.758134	-1.145917	-2.731026

1	0	0.854488	-1.160263	-3.251815
6	0	2.074231	-2.303335	-2.134074
8	0	2.934317	-2.401379	-1.250858
6	0	1.256816	-3.488569	-2.604055
1	0	0.936723	-3.330762	-3.634017
1	0	1.896785	-4.370133	-2.557704
6	0	0.034467	-3.715554	-1.699277
1	0	-0.553271	-4.536964	-2.123359
1	0	0.377696	-4.016153	-0.705131
6	0	-0.875709	-2.494702	-1.508263
1	0	-0.317630	-1.693771	-1.015991
7	0	-1.999569	-2.866375	-0.593954
1	0	-2.099238	-2.172720	0.173997
1	0	-1.824088	-3.755718	-0.128238
6	0	-1.519164	-1.940550	-2.819762
8	0	-0.741185	-1.432411	-3.645756
8	0	-2.760323	-2.056366	-2.895717
1	0	-2.869909	-2.872404	-1.154797
6	0	2.258646	0.162577	-0.749107
8	0	1.246651	-0.075472	-0.105686
7	0	3.438433	0.425670	-0.148439
1	0	4.267890	0.527018	-0.710818
6	0	3.523438	0.464886	1.286693
1	0	2.798083	1.181011	1.685717
1	0	4.516963	0.797172	1.588605
6	0	3.249687	-0.854220	2.003846
8	0	3.149448	-0.844298	3.216852
8	0	3.109691	-1.976456	1.346695
1	0	3.090268	-1.944310	0.353888

Compound **3a**
Level M06-2X/BSS-A
Energy HF = -6815.7815101 au
Standard orientation

34	0	-2.226316	-3.356874	-0.164871
34	0	-3.271483	-1.499631	-1.094878
6	0	-4.113785	-0.612664	0.454196
6	0	-0.473314	-2.728472	0.463088
1	0	-0.628354	-1.932398	1.185246
1	0	-0.097690	-3.605055	0.997010
1	0	-4.357326	-1.386833	1.179255
1	0	-5.032834	-0.195229	0.040862
6	0	0.520607	-2.369642	-0.634464
6	0	-3.301183	0.501779	1.110798
1	0	-3.989581	1.117961	1.701274
7	0	-2.689241	1.389715	0.145772
1	0	-1.992522	0.965264	-0.458793
6	0	-2.289793	-0.046301	2.135124
8	0	-2.428392	-1.134919	2.659075

7	0	-1.280279	0.806689	2.444814
1	0	-1.208413	1.695738	1.969877
6	0	-0.332386	0.532442	3.491643
1	0	-0.737098	-0.244532	4.143637
1	0	-0.154674	1.425170	4.092704
6	0	1.028951	0.036615	3.017211
8	0	1.927464	-0.152641	3.794037
8	0	1.172658	-0.223388	1.720821
1	0	0.423613	0.097299	1.178546
6	0	-2.643093	2.740151	0.327597
8	0	-3.215115	3.308624	1.239018
6	0	-1.833927	3.480859	-0.725254
1	0	-1.101666	2.791075	-1.143714
1	0	-2.525029	3.732096	-1.534387
6	0	-1.164602	4.743782	-0.160703
1	0	-1.488891	4.879475	0.873137
1	0	-1.502391	5.631522	-0.702162
6	0	0.358429	4.734501	-0.203133
1	0	0.726623	5.604302	0.357209
7	0	0.871236	4.782041	-1.593997
1	0	0.654779	5.691007	-1.994465
1	0	0.450863	3.573135	-2.629457
6	0	0.978254	3.525322	0.495405
8	0	2.289499	3.536279	0.390419
1	0	1.886548	4.698146	-1.566824
8	0	0.337983	2.692385	1.094204
1	0	0.400198	-3.067716	-1.466460
7	0	1.887533	-2.533134	-0.166041
1	0	2.150795	-2.270337	0.784584
6	0	2.885936	-2.748571	-1.058725
8	0	2.685060	-2.780083	-2.268213
6	0	4.253587	-3.039591	-0.466566
1	0	4.121071	-3.637264	0.436622
1	0	4.765347	-3.652333	-1.209801
6	0	5.148173	-1.828754	-0.151791
1	0	6.103902	-2.232780	0.195488
1	0	5.357638	-1.273242	-1.067937
6	0	4.634253	-0.860129	0.926574
1	0	5.495900	-0.331319	1.354448
7	0	3.857798	-1.569886	1.948958
1	0	3.459461	-0.921530	2.628871
1	0	4.469160	-2.199670	2.459703
6	0	3.754420	0.255974	0.374072
8	0	3.228819	-0.012188	-0.796484
8	0	3.593665	1.295741	0.983508
1	0	2.697976	2.680231	0.691776
6	0	0.260550	-0.959969	-1.200763
8	0	-0.296959	-0.068588	-0.561017
7	0	0.651995	-0.779396	-2.474645
1	0	1.266023	-1.486410	-2.874598

6	0	0.256655	0.394799	-3.208944
1	0	-0.833530	0.486982	-3.214782
1	0	0.582059	0.297480	-4.246073
6	0	0.802301	1.705032	-2.670247
8	0	0.180256	2.741724	-3.169400
8	0	1.706934	1.805354	-1.863202
1	0	2.634761	0.709338	-1.136209

Compound **3b**
Level M06-2X/BSS-A
Energy HF = -6815.7763165 au
Standard orientation

34	0	-1.911491	-3.340061	-0.354829
34	0	-3.192682	-1.687598	-1.363456
6	0	-4.211744	-0.939388	0.147473
6	0	-0.380404	-2.343771	0.372436
1	0	-0.737185	-1.414498	0.804142
1	0	-0.022918	-2.983948	1.181730
1	0	-4.500288	-1.759963	0.801467
1	0	-5.098986	-0.526680	-0.335773
6	0	0.742530	-2.089958	-0.627502
6	0	-3.510168	0.160279	0.947263
1	0	-4.284583	0.756146	1.444358
7	0	-2.773663	1.081487	0.112851
1	0	-2.002882	0.710401	-0.436748
6	0	-2.669848	-0.413558	2.104481
8	0	-2.936818	-1.484053	2.618131
7	0	-1.679187	0.402748	2.530949
1	0	-1.460705	1.234690	1.996285
6	0	-0.812839	0.058204	3.630881
1	0	-1.013860	-0.983097	3.901018
1	0	-0.989588	0.682711	4.508294
6	0	0.650557	0.197311	3.257420
8	0	1.505756	0.611595	3.996355
8	0	0.891126	-0.203891	2.009803
1	0	1.766832	0.136973	1.740689
6	0	-2.949599	2.428216	0.177552
8	0	-3.734586	2.964517	0.938210
6	0	-2.118795	3.223832	-0.818151
1	0	-1.296365	2.609253	-1.189808
1	0	-2.775734	3.412118	-1.671825
6	0	-1.632480	4.550461	-0.211735
1	0	-2.095893	4.674872	0.768929
1	0	-1.967216	5.398830	-0.814794
6	0	-0.120745	4.662787	-0.061604
1	0	0.107540	5.538146	0.561690
7	0	0.553782	4.788376	-1.375132
1	0	0.245044	5.644330	-1.827794
1	0	0.483175	3.525745	-2.396066

6	0	0.489834	3.473881	0.672435
8	0	1.807929	3.471155	0.542695
1	0	1.558426	4.861929	-1.226251
8	0	-0.139697	2.664120	1.303661
1	0	0.758455	-2.884842	-1.378431
7	0	2.056809	-2.127811	0.018529
1	0	2.126131	-1.975577	1.020035
6	0	3.142843	-2.586088	-0.657630
8	0	3.128687	-2.754865	-1.869941
6	0	4.372957	-2.893981	0.172377
1	0	4.074943	-3.254805	1.157634
1	0	4.890416	-3.697982	-0.351647
6	0	5.330275	-1.704425	0.315041
1	0	6.221573	-2.054003	0.843741
1	0	5.654785	-1.363544	-0.669375
6	0	4.758870	-0.514372	1.092487
1	0	5.603761	0.156970	1.321230
7	0	4.036816	-0.948464	2.286049
1	0	3.700323	-0.156162	2.829692
1	0	4.649408	-1.488125	2.888435
6	0	3.826826	0.377138	0.289431
8	0	4.046224	0.433922	-0.989361
8	0	2.967749	1.036029	0.863533
1	0	2.186186	2.598402	0.784246
6	0	0.544548	-0.766902	-1.391536
8	0	-0.225759	0.114951	-1.046037
7	0	1.293099	-0.651876	-2.512165
1	0	1.973327	-1.377385	-2.722291
6	0	1.046584	0.460132	-3.397638
1	0	0.017008	0.447135	-3.759468
1	0	1.725408	0.392728	-4.249504
6	0	1.285147	1.795241	-2.709449
8	0	0.384966	2.691247	-2.991474
8	0	2.250467	2.009071	-1.991440
1	0	3.357323	1.027813	-1.427002

Compound **3c**
Level M06-2X/BSS-A
Energy HF = -6815.7682001 au
Standard orientation

34	0	0.192446	-2.084012	-0.473451
34	0	-1.403780	-3.532564	-1.314570
6	0	-2.926777	-2.326759	-1.693705
6	0	1.147785	-1.653810	-2.153410
1	0	1.601044	-2.577373	-2.508881
1	0	0.410972	-1.299407	-2.871074
1	0	-3.769958	-3.016627	-1.636611
1	0	-2.839795	-1.956187	-2.712963
6	0	2.218825	-0.591441	-1.949226

6	0	-3.185881	-1.134408	-0.761812
1	0	-4.260384	-0.928236	-0.818208
7	0	-2.551366	0.098190	-1.177259
1	0	-1.542820	0.154219	-1.299470
6	0	-2.950901	-1.479582	0.707985
8	0	-3.382909	-2.531189	1.174001
7	0	-2.272909	-0.584944	1.447930
1	0	-1.825417	0.209548	1.000259
6	0	-1.957406	-0.843585	2.840224
1	0	-2.849871	-1.220390	3.347901
1	0	-1.632001	0.084009	3.305489
6	0	-0.824940	-1.862150	2.991781
8	0	0.214415	-1.600498	3.538731
8	0	-1.048727	-3.063889	2.470457
1	0	-1.941726	-3.115933	2.065451
6	0	-3.296378	1.249582	-1.204432
8	0	-4.493156	1.248701	-0.981739
6	0	-2.544814	2.534355	-1.490443
1	0	-1.467195	2.376537	-1.460036
1	0	-2.802114	2.840760	-2.508320
6	0	-2.999461	3.630669	-0.524116
1	0	-4.064304	3.823261	-0.660132
1	0	-2.444774	4.549473	-0.736917
6	0	-2.828440	3.280106	0.968991
1	0	-3.356724	2.343088	1.169450
7	0	-3.412286	4.334714	1.775189
1	0	-2.919749	5.208150	1.618346
1	0	-3.345249	4.125257	2.764656
6	0	-1.351330	3.027611	1.202780
8	0	-0.684037	4.091278	1.597319
1	0	0.266804	3.960965	1.405163
8	0	-0.820021	1.945468	0.988505
1	0	2.839849	-0.558333	-2.852839
7	0	3.105624	-0.928534	-0.846649
1	0	2.789469	-1.551588	-0.096892
6	0	4.382093	-0.474318	-0.776422
8	0	4.841051	0.324649	-1.583695
6	0	5.193478	-1.033014	0.376873
1	0	5.022351	-2.109786	0.438153
1	0	6.241059	-0.864023	0.129519
6	0	4.889423	-0.377433	1.736178
1	0	5.497372	-0.884015	2.490449
1	0	5.196769	0.669012	1.722273
6	0	3.423114	-0.443306	2.179136
1	0	3.383311	-0.148660	3.240376
7	0	2.843192	-1.763209	1.945470
1	0	3.412388	-2.467347	2.405138
1	0	1.916055	-1.823856	2.366272
6	0	2.596168	0.636919	1.495922
8	0	3.084145	1.693976	1.142223

8	0	1.319043	0.336691	1.394872
1	0	0.748854	1.082521	1.093556
6	0	1.591804	0.813643	-1.834146
8	0	0.386702	1.001115	-1.754199
7	0	2.469757	1.839411	-1.908717
1	0	3.460731	1.615143	-1.979801
6	0	1.978066	3.173925	-2.168796
1	0	1.343895	3.192855	-3.056805
1	0	2.835703	3.829444	-2.333779
6	0	1.149007	3.786444	-1.049176
8	0	0.189772	4.469888	-1.264858
8	0	1.584315	3.594708	0.203908
1	0	2.261373	2.883831	0.341938

Compound **3d**
Level M06-2X/BSS-A
Energy HF = -6815.7632498 au
Standard orientation

34	0	-3.232067	0.504491	0.837901
34	0	-3.900608	-1.705029	0.373788
6	0	-3.543598	-1.741597	-1.562642
6	0	-2.447905	0.247258	2.631537
1	0	-2.934165	1.010699	3.238358
1	0	-2.768655	-0.729705	2.991481
1	0	-4.335924	-1.193401	-2.066703
1	0	-3.632575	-2.804219	-1.796094
6	0	-0.934489	0.394812	2.786905
6	0	-2.201095	-1.212722	-2.051168
1	0	-2.080209	-1.579146	-3.077611
7	0	-1.075050	-1.735437	-1.307815
1	0	-1.129427	-1.782657	-0.288927
6	0	-2.221028	0.313507	-2.258810
8	0	-3.231231	0.859779	-2.663272
7	0	-1.039375	0.953968	-2.128317
1	0	-0.299138	0.594400	-1.535113
6	0	-0.918860	2.325419	-2.548101
1	0	-1.306181	2.451150	-3.559437
1	0	0.143740	2.584967	-2.536039
6	0	-1.629792	3.361685	-1.698919
8	0	-2.064919	4.393648	-2.119350
8	0	-1.659949	3.050014	-0.382037
1	0	-2.149708	3.760434	0.057040
6	0	0.021131	-2.196464	-1.940957
8	0	0.178149	-2.067442	-3.158854
6	0	1.056968	-2.899549	-1.085673
1	0	0.962206	-2.563900	-0.051181
1	0	0.809537	-3.965674	-1.095568
6	0	2.486884	-2.689954	-1.592571
1	0	2.634575	-3.162556	-2.567400

1	0	3.167964	-3.166509	-0.884245
6	0	2.830656	-1.199470	-1.672060
1	0	2.219207	-0.669778	-0.943357
7	0	2.513601	-0.611882	-3.014071
1	0	1.648612	-1.026648	-3.399866
1	0	3.333876	-0.787375	-3.607755
6	0	4.313840	-0.859166	-1.424071
8	0	5.039999	-0.720127	-2.393765
1	0	2.440039	0.415496	-2.888314
8	0	4.630227	-0.700351	-0.189042
1	0	-0.748582	0.607565	3.843344
7	0	-0.407597	1.532575	2.055178
1	0	-0.662608	1.638012	1.075214
6	0	0.748210	2.118948	2.461407
8	0	1.357760	1.764879	3.462460
6	0	1.217072	3.300786	1.628279
1	0	0.639358	3.365116	0.707275
1	0	1.015072	4.197694	2.220780
6	0	2.720010	3.230758	1.337264
1	0	3.001821	4.079155	0.705124
1	0	3.265810	3.308101	2.282369
6	0	3.104715	1.918228	0.656035
1	0	2.931253	1.084233	1.337377
7	0	4.552242	1.894943	0.271867
1	0	4.827268	0.873043	0.126377
1	0	5.158165	2.348111	0.953266
6	0	2.305281	1.657316	-0.637447
8	0	1.140953	1.257732	-0.473163
8	0	2.913432	1.837913	-1.717825
1	0	4.624392	2.340444	-0.652891
6	0	-0.185934	-0.904954	2.459857
8	0	-0.512214	-1.653219	1.550317
7	0	0.825453	-1.187149	3.310285
1	0	1.201931	-0.409562	3.841319
6	0	1.609236	-2.386523	3.132474
1	0	0.946408	-3.230891	2.944217
1	0	2.161720	-2.590184	4.051700
6	0	2.621032	-2.344901	1.994542
8	0	3.087901	-3.353762	1.527923
8	0	2.930179	-1.120142	1.618036
1	0	3.643944	-1.099134	0.881242

Compound **3e**
Level M06-2X/BSS-A
Energy HF = -6815.7591179 au
Standard orientation

34	0	1.991669	-1.579326	0.544120
34	0	3.393469	-2.132433	-1.235594
6	0	2.908546	-0.885368	-2.697121

6	0	0.662275	-3.043681	0.561907
1	0	0.789005	-3.620497	-0.352693
1	0	0.912256	-3.661501	1.422740
1	0	2.073906	-1.300606	-3.255066
1	0	3.808925	-0.914701	-3.313676
6	0	-0.769313	-2.551110	0.687380
6	0	2.588822	0.565210	-2.341291
1	0	2.755902	1.165637	-3.245222
7	0	3.460139	1.113450	-1.312491
1	0	3.855507	0.488215	-0.610507
6	0	1.079774	0.744233	-2.041735
8	0	0.269261	-0.161231	-2.222462
7	0	0.706240	1.979771	-1.676543
1	0	1.401036	2.719643	-1.720840
6	0	-0.675257	2.305150	-1.435238
1	0	-1.309834	1.893245	-2.225846
1	0	-0.791731	3.389689	-1.461668
6	0	-1.213794	1.817351	-0.084646
8	0	-0.498989	1.270661	0.736301
8	0	-2.473696	2.067354	0.066103
1	0	-3.693149	2.363364	-0.936149
6	0	3.644418	2.444873	-1.149998
8	0	3.164070	3.274099	-1.919618
6	0	4.370496	2.878459	0.105550
1	0	5.164859	2.182052	0.368985
1	0	4.802820	3.856470	-0.105603
6	0	3.359951	3.022695	1.262717
1	0	2.587639	3.739953	0.964450
1	0	3.875914	3.443672	2.132056
6	0	2.649844	1.723528	1.684309
1	0	2.123305	1.300199	0.825728
7	0	1.715413	1.946585	2.789452
1	0	0.870934	1.401747	2.621670
1	0	1.437662	2.919588	2.846614
6	0	3.707907	0.705375	2.128388
8	0	3.723025	0.454531	3.423908
1	0	2.954511	0.951685	3.786831
8	0	4.488348	0.202282	1.354314
1	0	-1.431565	-3.406329	0.858042
7	0	-1.207334	-1.905831	-0.542015
1	0	-0.527276	-1.328530	-1.045396
6	0	-2.483959	-1.876676	-0.933300
8	0	-3.402255	-2.427542	-0.304545
6	0	-2.768209	-1.088722	-2.197749
1	0	-2.061687	-0.258388	-2.259950
1	0	-2.560422	-1.729611	-3.059472
6	0	-4.212391	-0.592362	-2.240978
1	0	-4.322311	0.103018	-3.075348
1	0	-4.898789	-1.420274	-2.438115
6	0	-4.634985	0.104896	-0.928367

1	0	-3.770034	0.270840	-0.291530
7	0	-5.595634	-0.747460	-0.167457
1	0	-5.649834	-0.457513	0.831824
1	0	-5.232016	-1.711479	-0.186491
6	0	-5.353112	1.420070	-1.229980
8	0	-4.590028	2.474595	-1.374487
8	0	-6.544691	1.409304	-1.406876
1	0	-6.520850	-0.656004	-0.600141
6	0	-0.936408	-1.564591	1.866120
8	0	0.005637	-1.110034	2.483573
7	0	-2.225146	-1.234570	2.099742
1	0	-2.950427	-1.760770	1.626967
6	0	-2.600709	-0.090152	2.910224
1	0	-1.852263	0.690341	2.771457
1	0	-2.682512	-0.345332	3.970900
6	0	-3.974114	0.326449	2.404069
8	0	-4.911551	-0.448505	2.499534
8	0	-4.111608	1.475032	1.798393
1	0	-3.268789	1.790234	1.276086

Compound **4a**
Level M06-2X/BSS-A
Energy HF = -1442.6293163 au
Standard orientation

16	0	1.363940	1.695487	0.706084
16	0	0.097611	2.387510	-0.767498
6	0	-0.953463	0.954780	-1.118903
6	0	2.394671	0.447429	-0.147429
6	0	-2.075941	0.631712	-0.110830
6	0	2.278614	-0.971458	0.466733
6	0	-2.443208	-0.857209	-0.218126
8	0	-2.148384	-1.545518	-1.164911
8	0	-3.145197	-1.313903	0.802339
7	0	-1.897745	0.945312	1.299746
1	0	-2.977462	1.166183	-0.432788
1	0	-3.105650	-0.622281	1.492118
1	0	-1.005947	0.588572	1.635817
1	0	-1.914379	1.946554	1.451630
1	0	-1.396436	1.179788	-2.092768
1	0	-0.304479	0.099272	-1.289109
1	0	2.439459	-0.903352	1.544398
1	0	3.435732	0.768880	-0.071289
1	0	2.119011	0.435960	-1.204443
7	0	3.219450	-1.925259	-0.093795
6	0	0.858205	-1.472666	0.288117
8	0	-0.021754	-1.295221	1.091666
8	0	0.665254	-2.047943	-0.899337
1	0	3.036921	-2.081085	-1.079500
1	0	4.173443	-1.600913	0.013061

1	0	-0.289578	-2.220750	-1.021583

Compound **4b**
Level M06-2X/BSS-A
Energy HF = -1442.6291965 au
Standard orientation

16	0	0.410376	1.942817	-0.634174
16	0	-0.900673	2.028908	0.935711
6	0	-1.708839	0.391278	1.002547
6	0	1.918486	1.131454	-0.017772
6	0	-2.055992	-0.180599	-0.366810
6	0	1.915600	-0.397455	-0.204914
6	0	-2.515044	-1.627590	-0.156442
8	0	-1.744778	-2.515710	0.086332
8	0	-3.830315	-1.807431	-0.235620
7	0	-3.110470	0.600251	-1.010356
1	0	-1.134936	-0.233686	-0.954717
1	0	-4.206826	-0.940201	-0.485036
1	0	-3.011535	0.592541	-2.019574
1	0	-3.062170	1.570726	-0.714854
1	0	-1.051666	-0.295134	1.527602
1	0	-2.621093	0.569572	1.582512
1	0	1.599977	-0.610879	-1.229985
1	0	2.042877	1.384441	1.036743
1	0	2.727629	1.589611	-0.592624
7	0	1.025903	-1.037538	0.738100
6	0	3.354538	-0.885734	-0.067269
8	0	3.784697	-1.499724	0.867755
8	0	4.112929	-0.513111	-1.113521
1	0	0.560889	-1.852623	0.349984
1	0	1.546624	-1.336507	1.557725
1	0	5.014214	-0.824777	-0.952203

Compound **4c**
Level M06-2X/BSS-A
Energy HF = -1442.6290467 au
Standard orientation

16	0	-0.622652	1.784461	-1.006404
16	0	0.419155	1.923396	0.756981
6	0	1.406191	0.402299	0.907313
6	0	-2.150572	0.932225	-0.519915
6	0	2.455139	0.168941	-0.175901
6	0	-1.937841	-0.464263	0.076047
6	0	3.493922	-0.841235	0.364981
8	0	3.958290	-0.759536	1.467501
8	0	3.827398	-1.783170	-0.506879
7	0	1.923345	-0.316785	-1.450429
1	0	3.012774	1.096822	-0.336124

1	0	3.264150	-1.610386	-1.294381
1	0	0.969000	-0.658998	-1.329480
1	0	1.878822	0.425355	-2.136707
1	0	1.913110	0.534246	1.867097
1	0	0.754601	-0.469677	0.985148
1	0	-1.441994	-0.336781	1.044557
1	0	-2.728434	0.864668	-1.449143
1	0	-2.703714	1.552479	0.184381
7	0	-1.120767	-1.304547	-0.780466
6	0	-3.309013	-1.070456	0.361643
8	0	-3.757480	-2.037811	-0.185746
8	0	-3.970390	-0.380934	1.305024
1	0	-1.092045	-2.247596	-0.402499
1	0	-1.568341	-1.395352	-1.689876
1	0	-4.829519	-0.804628	1.439848

Compound **4d**
Level M06-2X/BSS-A
Energy HF = -1442.6281165 au
Standard orientation

16	0	0.955281	1.761880	-0.743882
16	0	-0.574851	2.396816	0.470558
6	0	-1.463832	0.862648	0.902118
6	0	2.148300	1.018624	0.411578
6	0	-1.818612	-0.013359	-0.298502
6	0	1.907831	-0.467189	0.672710
6	0	-2.186356	-1.389862	0.253218
8	0	-1.346403	-2.140327	0.690914
8	0	-3.473916	-1.672448	0.257389
7	0	-2.938257	0.548821	-1.046577
1	0	-0.918415	-0.155144	-0.906424
1	0	-3.919134	-0.911941	-0.170794
1	0	-2.865106	0.330331	-2.034552
1	0	-2.945546	1.561312	-0.970050
1	0	-0.881578	0.297704	1.627648
1	0	-2.378624	1.202144	1.400117
1	0	0.948879	-0.612573	1.175199
1	0	2.142903	1.578591	1.345836
1	0	3.121527	1.135883	-0.073159
7	0	2.957332	-0.945768	1.567654
6	0	1.923004	-1.225335	-0.664858
8	0	2.799384	-1.049332	-1.465000
8	0	0.960176	-2.125297	-0.874014
1	0	2.762795	-1.886821	1.892253
1	0	3.843673	-0.970045	1.070860
1	0	0.292291	-2.143886	-0.163190

Compound **4e**
Level M06-2X/BSS-A
Energy HF = -1442.6259512 au
Standard orientation

16	0	-1.758146	1.729896	-0.785701
16	0	0.003183	2.186558	0.161874
6	0	1.239550	1.050464	-0.560528
6	0	-2.631525	0.561214	0.312089
6	0	1.893658	0.166667	0.523344
6	0	-2.606471	-0.912820	-0.131119
6	0	2.692706	-0.890953	-0.209600
8	0	2.189656	-1.832315	-0.779347
8	0	4.002916	-0.659597	-0.242337
7	0	2.701471	0.871385	1.498545
1	0	1.109032	-0.375481	1.054948
1	0	4.416756	-1.364139	-0.760947
1	0	3.407954	1.449564	1.055958
1	0	2.115614	1.459303	2.080575
1	0	0.731808	0.461368	-1.324623
1	0	2.006220	1.665652	-1.040854
1	0	-2.891192	-0.991023	-1.181737
1	0	-3.669773	0.904154	0.322507
1	0	-2.224019	0.662101	1.320055
7	0	-3.476275	-1.761793	0.671046
6	0	-1.197855	-1.451639	0.038923
8	0	-0.715054	-1.634117	1.129569
8	0	-0.559270	-1.660740	-1.103295
1	0	-3.128381	-1.807417	1.624245
1	0	-4.423286	-1.401292	0.689008
1	0	0.365938	-1.936633	-0.920410

Compound **5a**
Level M06-2X/BSS-A
Energy HF = -3446.0085859 au
Standard orientation

16	0	0.077699	-1.921684	1.013412
34	0	-1.406712	-1.307633	-0.487350
6	0	-2.551033	-0.078165	0.556851
6	0	1.338813	-0.605176	1.006336
6	0	-2.404257	1.382594	0.122108
6	0	1.938997	-0.320484	-0.371016
6	0	-1.009327	1.860800	0.525118
8	0	-0.644171	1.885262	1.673544
8	0	-0.239508	2.184760	-0.508576
7	0	-2.768899	1.534978	-1.273855
1	0	-3.106425	1.958736	0.733713
1	0	0.685478	2.342301	-0.209073
1	0	-2.013105	1.195159	-1.861883
1	0	-2.886707	2.514837	-1.505107

1	0	-3.575259	-0.403592	0.379718
1	0	-2.281916	-0.218625	1.602549
1	0	1.130868	-0.012111	-1.042357
1	0	2.116085	-0.987527	1.678162
1	0	0.926338	0.302925	1.443704
7	0	2.700752	-1.466025	-0.862635
6	0	2.867626	0.888568	-0.211865
8	0	2.450400	2.010043	-0.054038
8	0	4.157223	0.605306	-0.224798
1	0	2.656941	-1.531509	-1.873724
1	0	2.314810	-2.330455	-0.494188
1	0	4.218470	-0.358136	-0.395018

Compound **5b**
Level M06-2X/BSS-A
Energy HF = -3446.0026241 au
Standard orientation

16	0	-1.875670	-1.702064	0.502745
34	0	-0.133458	-1.220709	-0.732024
6	0	1.344298	-1.259398	0.579574
6	0	-2.416830	-0.122677	1.252144
6	0	2.614597	-0.598805	0.011327
6	0	-2.395275	1.129560	0.371333
6	0	2.278458	0.862455	-0.262880
8	0	2.286703	1.706931	0.603018
8	0	1.908414	1.094174	-1.514898
7	0	3.684136	-0.777422	0.975207
1	0	2.890547	-1.080018	-0.930139
1	0	1.412165	1.932180	-1.542862
1	0	4.573483	-0.484407	0.585516
1	0	3.507642	-0.195556	1.789323
1	0	1.012799	-0.721342	1.469693
1	0	1.570780	-2.293302	0.829620
1	0	-2.938145	1.888436	0.953946
1	0	-1.851217	0.055870	2.163931
1	0	-3.455601	-0.336992	1.515890
7	0	-3.094922	0.937894	-0.876175
6	0	-0.999031	1.742580	0.236550
8	0	-0.576068	2.272099	-0.761575
8	0	-0.334988	1.720834	1.395141
1	0	-2.950530	1.739985	-1.480392
1	0	-2.727785	0.127511	-1.364751
1	0	0.549851	2.106883	1.260116

Compound **5c**
Level M06-2X/BSS-A
Energy HF = -3446.0019381 au
Standard orientation

16	0	-0.845057	-1.746305	0.884283
34	0	0.334495	-1.213319	-0.895960
6	0	2.136125	-1.118611	-0.085791
6	0	-1.462291	-0.178086	1.573075
6	0	2.814937	0.248421	-0.218952
6	0	-2.579915	0.502854	0.763731
6	0	1.831845	1.301934	0.275776
8	0	1.464177	1.349147	1.423708
8	0	1.413129	2.110802	-0.687653
7	0	4.049825	0.197407	0.551428
1	0	3.054453	0.454071	-1.263590
1	0	0.603778	2.568939	-0.388171
1	0	4.619871	1.016874	0.372049
1	0	3.824208	0.196106	1.542707
1	0	1.985424	-1.349878	0.969388
1	0	2.758724	-1.886464	-0.541451
1	0	-3.093686	1.182121	1.451298
1	0	-0.636140	0.514020	1.742327
1	0	-1.860688	-0.489816	2.543217
7	0	-3.551697	-0.396491	0.148843
6	0	-1.992779	1.446891	-0.299612
8	0	-1.196800	2.304770	-0.012900
8	0	-2.470234	1.279325	-1.518042
1	0	-4.442108	-0.395803	0.627690
1	0	-3.196545	-1.348008	0.111073
1	0	-3.125826	0.552362	-1.444595

Compound **5d**
Level M06-2X/BSS-A
Energy HF = -3446.0011215 au
Standard orientation

16	0	-1.871372	-1.452508	1.079180
34	0	-0.344268	-1.304894	-0.507384
6	0	1.181629	-0.686515	0.570802
6	0	-2.288743	0.279350	1.473724
6	0	2.090906	0.106092	-0.370591
6	0	-2.537861	1.206381	0.279447
6	0	3.370301	0.477720	0.369899
8	0	4.472231	0.117627	0.058752
8	0	3.125027	1.246768	1.441721
7	0	2.333039	-0.658334	-1.579946
1	0	1.572005	1.035456	-0.623418
1	0	3.972148	1.439964	1.865798
1	0	3.039775	-1.368542	-1.409515
1	0	2.693506	-0.063329	-2.316607
1	0	0.803492	-0.052680	1.370044
1	0	1.687103	-1.561053	0.983875
1	0	-3.053010	2.091109	0.672209
1	0	-1.509807	0.714990	2.099689

1	0	-3.200390	0.188273	2.070517
7	0	-3.337790	0.674859	-0.817348
6	0	-1.218183	1.790820	-0.259291
8	0	-0.298724	2.082112	0.457822
8	0	-1.217944	2.038192	-1.563328
1	0	-3.185834	-0.326260	-0.912504
1	0	-4.327568	0.822384	-0.665403
1	0	-2.044121	1.649889	-1.913121

Compound **5e**
Level M06-2X/BSS-A
Energy HF = -3445.9981322 au
Standard orientation

16	0	0.996972	-1.644674	0.522176
34	0	-0.835997	-1.265049	-0.646502
6	0	-2.117509	-0.914689	0.821609
6	0	1.410301	-0.052528	1.304582
6	0	-2.908087	0.368545	0.576923
6	0	2.108413	0.980565	0.423840
6	0	-1.917539	1.538966	0.463700
8	0	-1.241363	1.895485	1.389454
8	0	-1.841038	2.106482	-0.735190
7	0	-3.709427	0.277607	-0.644446
1	0	-3.513069	0.565403	1.469286
1	0	-2.432189	1.596609	-1.323853
1	0	-3.938759	-0.680769	-0.880895
1	0	-4.580344	0.790194	-0.565628
1	0	-2.790961	-1.766346	0.923853
1	0	-1.523448	-0.820151	1.729177
1	0	2.415699	1.799202	1.086323
1	0	2.097197	-0.358457	2.097884
1	0	0.518611	0.376828	1.762218
7	0	1.335237	1.575800	-0.661373
6	0	3.429107	0.411269	-0.121959
8	0	4.109864	-0.352299	0.501655
8	0	3.764169	0.877589	-1.321536
1	0	0.719016	2.302741	-0.313115
1	0	0.735448	0.885352	-1.103319
1	0	3.023309	1.451181	-1.602194

Compound **6a**
Level M06-2X/BSS-A
Energy HF = -5449.3744678 au
Standard orientation

34	0	-0.221890	-0.949864	-1.289465
34	0	-1.423724	-1.470442	0.634531
6	0	-1.805643	0.227218	1.580206
6	0	1.638208	-0.982265	-0.649037

6	0	-2.303671	1.413553	0.754190
6	0	2.038039	0.308142	0.079900
6	0	-1.151319	2.092627	0.009552
8	0	-1.164635	2.377180	-1.157662
8	0	-0.131519	2.357704	0.832257
7	0	-3.438101	1.065617	-0.068534
1	0	-2.629282	2.156522	1.495300
1	0	0.676252	2.529770	0.293038
1	0	-3.189462	0.312648	-0.703230
1	0	-3.697572	1.856873	-0.648284
1	0	-2.605316	-0.093296	2.251644
1	0	-0.933278	0.507431	2.163646
1	0	1.412608	0.386788	0.976826
1	0	2.242257	-1.116015	-1.551573
1	0	1.752758	-1.851066	-0.002590
7	0	1.929087	1.525908	-0.714707
6	0	3.473865	0.155340	0.547951
8	0	4.416438	0.723300	0.070941
8	0	3.574822	-0.732757	1.547074
1	0	2.830620	1.764503	-1.117544
1	0	1.246539	1.421034	-1.462204
1	0	4.510122	-0.816580	1.777949

Compound **6b**
Level M06-2X/BSS-A
Energy HF = -5449.373934 au
Standard orientation

34	0	-0.921922	-1.653792	0.548847
34	0	0.480949	-0.903805	-1.154050
6	0	2.235228	-1.040778	-0.246524
6	0	-1.492389	-0.008089	1.470660
6	0	2.981359	0.292570	-0.138839
6	0	-2.511105	0.853740	0.712270
6	0	2.016677	1.316719	0.446257
8	0	1.581038	1.223516	1.567447
8	0	1.696425	2.271715	-0.415378
7	0	4.162553	0.066583	0.682417
1	0	3.294533	0.631917	-1.127557
1	0	0.885558	2.721698	-0.106874
1	0	3.877911	-0.060222	1.650028
1	0	4.781667	0.869275	0.651365
1	0	2.022252	-1.422002	0.752379
1	0	2.843526	-1.762273	-0.788812
1	0	-2.968630	1.519311	1.451781
1	0	-0.611333	0.575698	1.737098
1	0	-1.949103	-0.400140	2.382854
7	0	-3.562623	0.132718	0.000613
6	0	-1.798872	1.821930	-0.248057
8	0	-0.924995	2.559157	0.131914

8	0	-2.254722	1.817359	-1.485687
1	0	-3.303830	-0.837273	-0.157775
1	0	-4.451589	0.163789	0.481118
1	0	-2.994768	1.172887	-1.490915

Compound **6c**
Level M06-2X/BSS-A
Energy HF = -5449.3732093 au
Standard orientation

34	0	0.348849	-0.889881	-0.928490
34	0	-1.549536	-1.475135	0.290809
6	0	-1.906565	0.142971	1.354755
6	0	1.756864	-1.499767	0.315673
6	0	-2.415360	1.344359	0.551845
6	0	2.514586	-0.353022	0.965297
6	0	-1.254675	2.094813	-0.131893
8	0	-0.253222	2.399555	0.459528
8	0	-1.480568	2.427154	-1.391881
7	0	-3.469038	1.063685	-0.419938
1	0	-2.796879	2.073292	1.275414
1	0	-2.375450	2.086798	-1.600354
1	0	-4.392536	1.254727	-0.054675
1	0	-3.431880	0.094763	-0.726887
1	0	-2.678447	-0.185196	2.055226
1	0	-1.006101	0.394283	1.914474
1	0	3.281239	-0.792558	1.616010
1	0	2.424547	-2.123886	-0.276590
1	0	1.260572	-2.095760	1.078101
7	0	1.646618	0.466842	1.796623
6	0	3.270770	0.436242	-0.101578
8	0	3.296168	0.173085	-1.273443
8	0	3.924217	1.485155	0.424116
1	0	1.060206	1.068597	1.219623
1	0	2.204161	1.084819	2.376168
1	0	4.344370	1.963804	-0.303312

Compound **6d**
Level M06-2X/BSS-A
Energy HF = -5449.3731001 au
Standard orientation

34	0	1.926883	-1.315289	-0.639795
34	0	0.177391	-0.994477	0.873165
6	0	-1.317652	-0.751545	-0.388735
6	0	2.235481	0.515774	-1.315721
6	0	-2.361126	0.108587	0.327809
6	0	2.186524	1.636097	-0.277548
6	0	-3.611839	0.196272	-0.539463
8	0	-4.695165	-0.213326	-0.223847

8	0	-3.362241	0.771934	-1.725225
7	0	-2.620956	-0.428879	1.650050
1	0	-1.950040	1.118179	0.417872
1	0	-4.191109	0.791682	-2.222587
1	0	-3.239928	-1.232219	1.585180
1	0	-3.093695	0.254199	2.230163
1	0	-0.942083	-0.249040	-1.276732
1	0	-1.711201	-1.737134	-0.643691
1	0	2.622485	2.521975	-0.756527
1	0	1.512050	0.724202	-2.101796
1	0	3.232546	0.452364	-1.756821
7	0	2.898737	1.426696	0.977575
6	0	0.739565	2.083227	0.002392
8	0	-0.115491	2.083242	-0.841628
8	0	0.542142	2.565925	1.223868
1	0	2.854341	0.449302	1.255482
1	0	3.875722	1.679714	0.899554
1	0	1.361438	2.381422	1.724218

Compound **6e**
Level M06-2X/BSS-A
Energy HF = -5449.3730444 au
Standard orientation

34	0	2.007617	-1.015749	-0.644446
34	0	0.078002	-1.850515	0.356679
6	0	-1.309522	-0.827762	-0.599911
6	0	2.504211	0.453935	0.578454
6	0	-2.275867	-0.197585	0.402572
6	0	2.124017	1.859988	0.097651
6	0	-3.106203	0.861248	-0.319076
8	0	-2.636756	1.756278	-0.982820
8	0	-4.424114	0.732000	-0.148288
7	0	-3.003701	-1.239741	1.095521
1	0	-1.685828	0.369960	1.129436
1	0	-4.853086	1.459406	-0.621708
1	0	-3.525877	-0.872616	1.881598
1	0	-3.663627	-1.701819	0.478829
1	0	-0.799239	-0.087963	-1.211045
1	0	-1.844220	-1.519706	-1.254341
1	0	2.512531	2.036192	-0.906668
1	0	3.589283	0.388549	0.684528
1	0	2.039007	0.242914	1.542440
7	0	2.602314	2.915068	0.985596
6	0	0.610763	1.990287	0.062171
8	0	-0.060740	1.906989	1.062308
8	0	0.113001	2.191604	-1.149455
1	0	2.172718	2.811330	1.900518
1	0	3.608305	2.860729	1.099344
1	0	-0.866847	2.203085	-1.091813

Compound **7**
Level M06-2X/BSS-A
Energy HF = -876.1780226 au
Standard orientation

1	0	-0.268681	2.360564	0.567134
1	0	-1.987697	1.976908	0.839386
1	0	-0.738540	1.123144	1.771252
6	0	-0.975900	1.568678	0.806193
16	0	-0.975900	0.312015	-0.500933
16	0	0.975900	-0.312015	-0.500933
6	0	0.975900	-1.568678	0.806193
1	0	0.268681	-2.360564	0.567134
1	0	1.987697	-1.976908	0.839386
1	0	0.738540	-1.123144	1.771252

Compound **8**
Level M06-2X/BSS-A
Energy HF = -2879.5481642 au
Standard orientation

1	0	-2.148588	-0.029279	-1.563347
1	0	-3.184576	0.690641	-0.306100
1	0	-1.748779	1.571709	-0.872671
6	0	-2.158880	0.584685	-0.664883
16	0	-1.239734	-0.227720	0.671789
34	0	0.763085	-0.437699	-0.194718
6	0	1.498186	1.318143	0.263994
1	0	1.454188	1.455744	1.341276
1	0	2.535741	1.317693	-0.071451
1	0	0.947044	2.101825	-0.250577

Compound **9**
Level M06-2X/BSS-A
Energy HF = -4882.9205598 au
Standard orientation

1	0	1.569891	1.926018	0.829660
1	0	0.143184	2.945255	1.161478
1	0	0.360602	1.415840	2.047846
6	0	0.516762	1.922679	1.098734
34	0	-0.516762	1.040553	-0.312688
34	0	0.516762	-1.040553	-0.312688
6	0	-0.516762	-1.922679	1.098734
1	0	-1.569891	-1.926018	0.829660
1	0	-0.143184	-2.945255	1.161478
1	0	-0.360602	-1.415840	2.047846
